# *In situ* Rb–Sr dating by collision cell, multicollection inductively-coupled plasma mass-spectrometry with pre-cell mass-filter, (CC-MC-ICPMS/MS)†

**DOI:** 10.1039/d1ja00006c

**Published:** 2021-04-01

**Authors:** Dan Bevan, Christopher D. Coath, Jamie Lewis, Johannes Schwieters, Nicholas Lloyd, Grant Craig, Henning Wehrs, Tim Elliott

**Affiliations:** School of Earth Sciences, University of Bristol Wills Memorial Building, Queens Road Bristol BS8 1RJ UK dan.bevan@bristol.ac.uk; Thermo Fisher Scientific (Bremen) GmbH Hanna-Kunath-Str. 11 28199 Bremen Germany

## Abstract

We document the utility for *in situ* Rb–Sr dating of a one-of-a-kind tribrid mass spectrometer, ‘Proteus’, coupled to a UV laser ablation system. Proteus combines quadrupole mass-filter, collision cell and sector magnet with a multicollection inductively-coupled plasma mass spectrometer (CC-MC-ICPMS/MS). Compared to commercial, single collector, tribrid inductively-coupled plasma mass spectrometers (CC-ICPMS/MS) Proteus has enhanced ion transmission and offers simultaneous collection of all Sr isotopes using an array of Faraday cups. These features yield improved precision in measured ^87^Sr/^86^Sr ratios, for a given mass of Sr analysed, approximately a factor of 25 in comparison to the Thermo Scientific™ iCAP TQ™ operated under similar conditions. Using SF_6_ as a reaction gas on Proteus, measurements of Rb-doped NIST SRM (standard reference material) 987 solutions, with Rb/Sr ratios from 0.01–100, yield ^87^Sr/^86^Sr that are indistinguishable from un-doped NIST SRM 987, demonstrating quantitative ‘chemical resolution’ of Rb from Sr. We highlight the importance of mass-filtering before the collision cell for laser ablation ^87^Sr/^86^Sr analysis, using an in-house feldspar standard and a range of glass reference materials. By transmitting only those ions with mass-to-charge ratios 82–92 u/e into the collision cell, we achieve accurate ^87^Sr/^86^Sr measurements without any corrections for atomic or polyatomic isobaric interferences. Without the pre-cell mass-filtering, measured *in situ*^87^Sr/^86^Sr ratios are inaccurate. Combining *in situ* measurements of Rb/Sr and radiogenic Sr isotope ratios we obtain mineral isochrons. We utilise a sample from the well-dated Dartmoor granite (285 ± 1 Ma) as a calibrant for our *in situ* ages and, using the same conditions, produce accurate Rb–Sr isochron ages for samples of the Fish Canyon tuff (28 ± 2 Ma) and Shap granite pluton (397 ± 1 Ma). Analysing the same Dartmoor granite sample using identical laser conditions and number of spot analyses using the Thermo Scientific™ iCAP TQ™ yielded an isochron slope 5× less precise than Proteus. We use an uncertainty model to illustrate the advantage of using Proteus over single collector CC-ICPMS/MS for *in situ* Rb–Sr dating. The results of this model show that the improvement is most marked for samples that have low Rb/Sr (<10) or are young (<100 Ma). We also report the first example of an *in situ*, internal Rb–Sr isochron from a single potassium-feldspar grain. Using a sample from the Shap granite, we obtained accurate age and initial ^87^Sr/^86^Sr with 95% confidence intervals of ±1.5% and ±0.03% respectively. Such capabilities offer new opportunities in geochronological studies.

## Abstract 2

This paper highlights the capabilities of a novel tribrid mass spectrometer for rapid, *in situ* geological age determinations of typical crustal rocks and single, K-feldspars.

## Graphical abstract

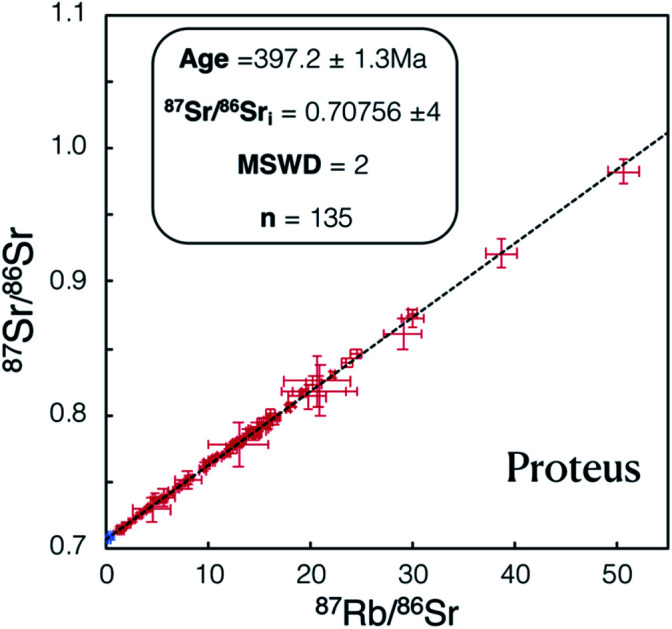

## 1. Introduction

Radiometric dating of geological materials using the ^87^Rb–^87^Sr beta decay system is a well-established geochronological technique which has been exploited for over 80 years.^1^ By its nature, beta decay produces a daughter nuclide that isobarically interferes with its parent, and *vice versa*, during mass-spectrometric analysis. At present, no mass spectrometer designed to measure isotope ratios to high precision possesses sufficient mass resolution to resolve ^87^Rb from ^87^Sr (required *M*/Δ*M* ∼ 300 000). For this reason, *in situ*^87^Sr/^86^Sr analysis has previously been limited to low Rb/Sr phases such as plagioclase or apatite.^2–5^ Therefore, most Rb–Sr dating has required analyses of bulk samples where Rb has been chemically separated from Sr after dissolution. However, the development and coupling of collision cells to inductively-coupled plasma mass spectrometers now permits *in situ* radiogenic Sr isotopic analysis of high Rb/Sr minerals^6^ by using a suitable reaction gas within a collision cell to react with the Sr^+^ ions but not the interfering Rb^+^ ions.^7–9^ There are a number of suitable reaction gases which are capable of providing such ‘chemical resolution’ of Rb^+^ from Sr^+^, which include N_2_O, O_2_, CH_3_F and SF_6_.^6,10–12^ All of these gases display reactivity with Sr^+^ and, crucially, little to no reactivity with Rb^+^.^13^ Such an approach has been successful in laser ablation Rb–Sr dating using single collector inductively coupled plasma mass spectrometers equipped with collision cells and pre-cell quadrupole mass-filters (CC-ICPMS/MS).^6,11,14,15^ Analytical precision is an important limit in such work and, as a result, many of the previous studies have focused on samples containing minerals with very high Rb/Sr (*e.g.*^87^Rb/^86^Sr ratios >500) or samples that are particularly old (>1 Ga).^6,11,15–17^ Here, we illustrate the potential of collision cell mass spectrometry for a wider range of geological targets using a novel multicollection system.

## 2. Methods and materials

### 2.1 Instrumentation

**Fig. 1:**
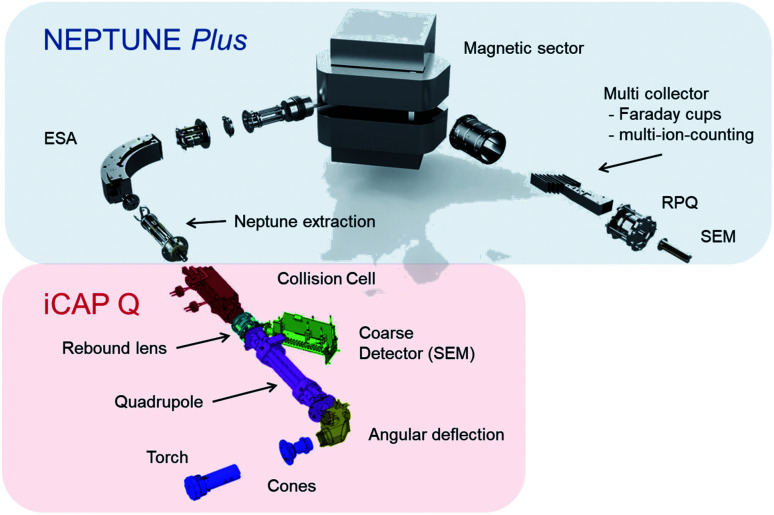
Diagram showing constituent components of Proteus. The red shaded rectangle highlights the ‘front-end’, comprised of parts (or equivalents) dominantly sourced from the Thermo Scientific™ iCAP Q™ instrument, while the blue area highlights the analyser section constructed from a Thermo Scientific™ Neptune Plus™.

In this study we document the performance of a unique, tribrid mass-spectrometer (collision cell, multicollector, inductively-coupled plasma mass spectrometer with pre-cell mass-filter, CC-MC-ICPMS/MS) developed by Thermo Fisher Scientific™ Bremen, in collaboration with the Bristol Isotope Group, which we dub ‘Proteus’. The instrument couples a new, low energy ‘front end’, containing components from the Thermo Scientific™ iCAP Q™ mass spectrometer with an analyser formed from a Thermo Scientific™ Neptune Plus™ (Fig. 1). A full description of the instrument is provided elsewhere, but here we briefly highlight some key features for this study.

Most importantly, Proteus has a magnetic sector and multicollection system, comprising of several movable Faraday cups, in contrast to the quadrupole mass-analyser and single electron multiplier detector in commercial CC-ICPMS/MS. Simultaneous ion collection all-but eliminates the noise in an isotope ratio resulting from ion-source instability. Moreover, Faraday cups are linear, stable, readily inter-calibrated detectors enabling measurements of sufficiently intense ion beams to higher accuracy than is possible with secondary-electron multipliers. In common with CC-ICPMS/MS, Proteus has a quadrupole mass-filter followed by a collision cell, a quadrupole ion guide into which different gases can be introduced to interact with the ions passing through. The collision cell is designed to remove isobaric interferences through ion–molecule reactions^9,10^ and the quadrupole mass-filter can be operated in band-pass mode to limit transmission into the collision cell of ions with a restricted range of mass/charge ratio. Proteus' collision cell has an axial electrostatic field (a ‘drag cell’) to accelerate ions through the collision cell, and a −2 kV post collision cell extraction potential. These features should enhance ion sensitivity relative to currently available, lower energy CC-ICPMS/MS with consequent higher precision Sr isotope ratio measurements for a given sample size.

Solution analyses were conducted using Proteus in an initial evaluation of SF_6_ as a reaction gas for measuring ^87^Sr/^86^Sr. Sample introduction was *via* a Teledyne™ CETAC Aridus II™ desolvator. A nebuliser, nominal uptake rate 50 μl min^−1^, was used to introduce solution into the Aridus II™. For *in situ* analysis, Proteus was coupled to a Teledyne™ Photon Machines 193 nm Analyte G2™ ArF excimer laser ablation system equipped with an ARIS (aerosol rapid introduction system)^18^ supplied with 0.9 l min^−1^ He carrier gas. A cold plasma Ni skimmer cone^19^ (P/N 1341420) was used for all analyses using Proteus.

In order to contrast Proteus with a commercial, single collector CC-ICPMS/MS, we used a Thermo Scientific™ iCAP TQ™ installed in the Research and Development laboratories of Thermo Fisher Scientific™ in Bremen, Germany. This instrument is comprised of a collision cell positioned between post- and pre-cell quadrupole mass analysers, and a single secondary-electron multiplier for ion beam collection. See Kutscher *et al.*^20^ for further information regarding the iCAP TQ™. From torch to quadrupole (Fig. 1), the fixed components in Proteus and the iCAP TQ™ are identical. Proteus further uses the terminal quadrupole from the iCAP TQ™ as its initial mass-filter. One notable difference in the source setup of iCAP TQ™ and Proteus in this study is that for the iCAP TQ™ we used a standard skimmer cone with “high sensitivity” insert, rather than the cold plasma cone used on Proteus, as experimentation with the cold plasma cone did not start until the iCAP TQ™ measurements were completed. For *in situ* analysis, the iCAP TQ™ was coupled to a Teledyne™ Photon Machines 193 nm Analyte G2™ ArF excimer laser ablation system equipped with an ARIS. We did not specifically test the performance of the laser ablation system in Bremen relative to the one in Bristol, but given common instrument models and set-up procedures, we have assumed equal ablation rates in later sections.

### 2.2 Standards and samples

External normalisation to reference materials was required to achieve accuracy when using both Proteus and the iCAP TQ™. An assortment of reference materials and in-house standards were used for normalisation in this study. For the solution analyses on Proteus, National Institute of Standards and Technology (NIST) Standard Reference Material® (SRM) 987 was used as a standard. Rubidium-doped NIST SRM 987 solutions (Rb/Sr of 0.01–100), with a Sr concentration of 0.05 μg g^−1^ were also prepared to assess the chemical resolution of Sr from Rb. The range of Rb/Sr in the doped NIST SRM 987 solutions were designed to be greater than the entire Rb/Sr range explored by *in situ* analysis in this study. The source of the Rb used to make the Rb-doped NIST SRM 987 was a CPI single element solution, with a Rb concentration of 1000 μg g^−1^ (99.975% purity) in a matrix of 0.3 M HNO_3_. All of the solutions analysed in this study were prepared and diluted in a 0.3 M HNO_3_ matrix.

For *in situ* analysis a variety of widely used reference materials (NIST SRM 610 glass and USGS glass standards BHVO-2G, BCR-2G and BIR-G) were employed. Literature values of Rb–Sr data for these reference materials are shown in Table 1. In addition, an in-house plagioclase standard was prepared to provide a more appropriate matrix match for feldspar analyses, which are an important focus of this study. Electron microprobe analysis revealed that large plagioclase phenocrysts (∼1 cm) from a recent eruption of Telica (Nicaragua) displayed low inter-grain heterogeneity in their major element chemistry (A*n*_91–92_, *n* = 5, where A*n* expresses, as a percentage, the proportion of the anorthite component in a feldspar, *i.e.* [Ca]/([Ca] + [Na] + [K]) × 100, for molar concentrations) and might therefore make a good standard. We prepared a mount of 5 such plagioclase crystals picked from a volcanic bomb and mounted them in epoxy resin. We confirmed they displayed little Sr concentration heterogeneity between and within grains from electron microprobe analysis. The Sr concentration (μg g^−1^) of the plagioclase grains ranged from 859 ± 153 to 818 ± 132 (2SD, *n* = 30–38 spot analyses). The inter-grain Sr concentration varies by ±3% (2 relative standard deviations (2RSD), *n* = 5), unresolvable within analytical uncertainty for the 5 grains analysed. The ^87^Sr/^86^Sr of the Telica plagioclase (Te-1) was characterised by thermal ionisation mass-spectrometry (TIMS) using a Thermo Finnigan™ Triton™. Three plagioclase feldspars were separated, ultrasonicated in acetone and 18.2 MΩ cm H_2_O before dissolution in 7 M HNO_3_ and 29 M HF. The Sr fraction was purified using Sr Spec resin,^21^ dried and loaded onto a standard purity, single Re filament with TaCl_5_ as an activator to improve ionisation efficiency.^22^ Measured ^87^Sr/^86^Sr ratios were corrected for mass-dependent fractionation by internal normalisation to ^86^Sr/^88^Sr = 0.1194 using the exponential law.^23^ A multi-dynamic method was used to eliminate the effect of Faraday cup efficiency factors on the accuracy of the ^87^Sr/^86^Sr ratio.^24^ The mean ^87^Sr/^86^Sr for the 3 grains was 0.704000_−2_^+3^ (total range), with an uncertainty for each analysis of 5 × 10^−6^ (2SE uncertainty, *n* = 200 cycles). Subsequent Proteus measurements of a single Te-1 plagioclase crystal, sampled by laser ablation, yielded ^87^Sr/^86^Sr = 0.704005 ± 23 (2SE, *n* = 54 spot analyses) after internal normalisation *via* the exponential law^23^ and external normalisation to BCR-2G, in agreement with the value determined by TIMS. At this level of precision, we cannot resolve possible heterogeneity from analytical uncertainty, but this measurement provides an upper limit on the Sr isotopic heterogeneity within a single plagioclase.

**Table 1:** Reference values used for external normalisation of ^87^Rb/^86^Sr and ^87^Sr/^86^Sr with associated uncertainties. For the relative isotopic abundances of ^86^Sr and ^87^Rb used to calculate the ^87^Rb/^86^Sr of NIST SRM 610 see ref. 33

Reference material	^87^Rb/^86^Sr	Rb (μg g^−1^)	2SD	Sr (μg g^−1^)	2SD	^87^Sr/^86^Sr	2SD
BIR-G						0.703105 (ref. 34)	0.000011
BCR-2G						0.705003 (ref. 34)	0.000008
BHVO-2G						0.703469 (ref. 34)	0.000014
SRM 610	2.3894	425.7 (ref. 35)	0.8	515.5 (ref. 35)	0.5	0.709699 (ref. 36)	0.000018
SRM 987						0.710251 (ref. 24)	0.000011

To test the capability of our *in situ* Rb–Sr dating method in producing precise ages for common rock types, we used three samples with relatively simple magmatic histories, from localities previously well characterised geochronologically spanning much of the Phanerozoic eon. Samples DG-1 and SG-1 are from two classic, English granite intrusions, Dartmoor and Shap respectively.^25,26^ Both granite samples contain euhedral K-feldspar megacrysts (>1 cm) in a matrix of plagioclase, biotite and quartz. The coarse grain size of plagioclase and K-feldspar in both samples permits the use of large (>100 μm) laser spot sizes. Although our samples were taken from collections that had not undertaken geochronological work, previous geochronological study of both intrusions provide well-constrained reference ages for these two bodies. Bulk TIMS Rb–Sr analyses for the Dartmoor granite yield a whole rock isochron age of 284.6 ± 1 Ma (ref. 27) (2*σ*), which is within error of a less precise mineral separate Rb–Sr isochron (282.5 ± 2 Ma).^27^ The Shap granite has also been dated yielding a whole rock Rb–Sr isochron age of 400.3 ± 3 Ma (ref. 28) (2*σ*). We also analysed a third sample from the Fish Canyon Tuff (FCT-1), which has been a focus for extensive chronological work^29–31^ including a Rb–Sr age of 27.9 ± 0.2 Ma (2*σ*) from the solution analysis of feldspars and mica.^32^ The FCT-1 sample contains sizeable phenocrysts (>5 mm) of biotite, plagioclase and K-feldspar set in a matrix of silicic glass.

**Fig. 2:**
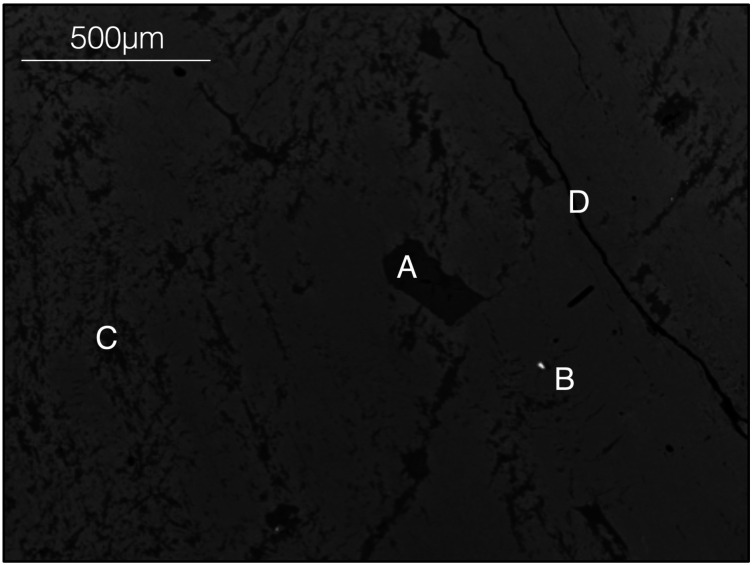
Back scattered electron image of the DG-1 Dartmoor granite sample used for *in situ* Rb–Sr dating using Proteus and iCAP TQ™. Such images were used as references during laser ablation to identify various features in the K-feldspars such as plagioclase inclusions (A), apatite inclusions (B), perthite exsolution (C) and fractures (D).

All of the samples in this study were prepared as polished fragments embedded in epoxy resin. Prior to the analysis on Proteus, the mounted samples were also characterised at the University of Bristol using the Hitachi S-3500N™ scanning electron microscope at a beam energy of 20 keV. The back-scattered electron images produced were used to avoid inclusions or fractures within K-feldspars (Fig. 2).

### 2.3 Analytical procedure

A prime consideration in the choice of reaction gas for this study is the chemical separation of Rb^+^ from Sr^+^. Sulfur hexafluoride, which has no observable reaction with Rb^+^ and exhibits an efficient exothermic reaction with Sr^+^ to form SrF^+^, was preferred over alternatives, such as O_2_ and N_2_O, because, (a) there is a lack of higher order product formation^11,13^ and (b) monoisotopic F ensures that SrF^+^ isotopologues do not isobarically interfere with each other.

#### 2.3.1 Proteus

The reaction gas mixture used was 5% SF_6_ (99.99% purity) in He (99.9999% purity) supplied by BOC. The 5% SF_6_ cylinder was connected to a temperature-controlled mass flow controller *via* electropolished stainless steel pipework and high-purity fittings. Other reaction gases share the mass flow controller and some of the gas delivery pipework necessitating a thorough purge of the gas line, before analysis, by filling with Ar gas (99.9999% purity) followed by venting, typically repeated a total of three times. The gas line was further purged overnight with the reaction gas mix at a low (0.2 ml min^−1^) gas flow into the collision cell with the instrument in standby. This purging regimen was sufficient to remove any memory of previously used reaction gases such as O_2_. A separate He gas cylinder (99.999% purity) was connected into the reaction gas stream *via* its own mass flow controller to further dilute the SF_6_, allowing for independently adjustable SF_6_ and He partial pressures in the collision cell. The gas delivery system was equipped with SAES gas purifiers (MC1 filter filled with 902 filter medium) to further purify both the He and SF_6_/He mix before entry to the collision cell.

To quantify the maximum SF_6_ + Sr^+^ → SF_5_ + SrF^+^ reaction efficiency, the Sr^+^ and SrF^+^ beam intensities were monitored as the SF_6_/He mix flow rate was progressively increased and the He flow rate decreased to maintain a constant total flow rate into the collision cell of 5 ml min^−1^. The optimum conditions for maximum SrF^+^ beam intensity were found at a SF_6_/He mix flow rate of 0.9 ml min^−1^ (*i.e.* 0.045 ml min^−1^ SF_6_), giving a conversion efficiency of ∼85%. Note that this efficiency does not account for the loss of ions to collisions in the cell, likely increasing with the partial pressure of SF_6_, so represents a lower-bound. For *in situ* analysis, the flow rate of SF_6_/He gas and drag cell voltages were further optimised in each analytical session to remove any detectable influence of cell-derived isobaric interferences. To achieve this, the SF_6_ flow rate and drag cell exit voltages were reduced until ^87^Sr^+^/^86^Sr^+^ of NIST 610 normalised with Te-1 was accurate. High SF_6_/He mix flow rates and extremely negative drag cell exit voltages were observed to decrease accuracy and, therefore, interpreted to result in transmission of significant intensities of cell derived interferences.

Unless stated otherwise, the pre-cell mass-filter was operated in ‘band-pass mode’, where ions with a restricted range of mass/charge are transmitted to the collision cell. In general, a narrow band-pass is preferred in order to remove potential atomic and molecular isobars at the masses of SrF and ^85^Rb. A band-pass allowing transmission of ions with mass/charge of 87 ± 5 u/e (unified atomic mass units per electronic charge) was found to be optimal. A narrower width adversely affected the transmission of the isotopes of interest. The band-pass window is set by ablating NIST SRM 610, which has peaks at almost every integer mass/charge, and adjusting the DC component of the quadrupole field.

For both solution and those *in situ* analyses where no Rb/Sr determination was required, static measurements were made, the cup configuration being that in the second line of Table 2 but with zero idle time between cycles. For *in situ* dating, a magnet jump is required to measure both the SrF^+^ isotopologues and the monatomic ions ^85^Rb^+^ and ^88^Sr^+^, see Table 2 for details. All Faraday cup amplifiers used in this study were equipped with 10^11^ Ω feedback resistors.

**Table 2:** Acquisition times, cup configuration and iCAP TQ™ quadrupole mass settings used for *in situ* Rb–Sr dating. Acquisition steps are repeated cyclically for the duration of the measurement, approx. 100 s or 60 s (see main text). (a) Proteus requires two magnet steps to measure all the required species. Pre-cell mass-filter transmits a fixed mass/charge range of 82–92 u/e. Note: although possible, we did not collect ^84^Sr, as the limited precision that can be obtained for this unabundant isotope does not yield useful additional information for this study. (b) iCAP TQ™ requires four mass steps, the mass/charge transmitted through third quadrupole, *u*_Q3_, at each step, is indicated by the ion species. The first quadrupole transmits ions with mass/charge in the range *u*_Q1_ – 9 u/e to *u*_Q1_

	Idle time (s)	Integration time^a^ (s)	*u*_Q1_	*u*_Q3_	L3	L2	L1	C	H1	H2	H3	H4
(a)	0.5	0.13			^85^Rb^+^		^88^Sr^+^					
	0.5	1.05						^86^SrF^+^	^87^SrF^+^	^88^SrF^+^		

(b)		0.1	^85^Rb^+^	^85^Rb^+^								
		0.1	^86^Sr^+^	^86^SrF^+^								
		0.1	^87^Sr^+^	^87^SrF^+^								
		0.1	^88^Sr^+^	^88^SrF^+^								

a iCAP TQ™ software uses the term dwell time which is analogous to the integration time.

Solution measurement tune conditions were optimised for maximum ^88^SrF^+^ sensitivity using a 0.2 μg g^−1^ solution of NIST SRM 987 (Table 3). The analysis of each solution was carried out, using a total integration time of 252 seconds, and was repeated 5 times for each Rb-doped solution. Measured ^87^SrF^+^/^86^SrF^+^ ratios were internally normalised to a ^86^SrF^+^/^88^SrF^+^ ratio of 0.1194 using the exponential mass fractionation law^23^ and the atomic Sr masses. No correction for isobaric ^87^RbF^+^ was made. The internally-normalised ^87^Sr/^86^Sr ratios for the Rb-doped NIST SRM 987 solutions were externally normalised to internally-normalised measurements of un-doped NIST SRM 987, assuming a true ^87^Sr/^86^Sr of 0.710251,^24^ to correct for any residual instrumental artefacts.

**Table 3:** Tune parameters used for solution analysis on Proteus and for *in situ* analysis using both Proteus and iCAP TQ™. Laser – MS refers to the tune conditions used to achieve maximum ^88^SrF^+^ sensitivity. Laser – REF refers to the tune conditions required to reduce elemental fractionation for *in situ* dating. The sole difference in plasma tune conditions between REF 1 and 2 for Proteus is a sample gas flow rates of 0.96 l min^−1^ and 0.88 l min^−1^ respectively. Entry and exit orifices to the Proteus collision cell are 2 mm in diameter

		Proteus (solution)	Proteus (laser – MS)	Proteus (laser – REF)	iCAP TQ™ (laser – REF)
**Laser**					
Spot diameter	μm		110	110	110
Fluence	J cm^−2^		6	6	6
Repetition rate	Hz		10	10	10
He flow rate	l min^−1^		0.90	0.90	0.90

**Desolvator (Aridus II™)**					
Ar sweep gas	l min^−1^	3.8			

**Plasma**					
RF power	W	1550	1550	1550	1550
Sampling depth	mm	6	7	15	5
Cool gas flow rate (Ar)	l min^−1^	15	15	15	14
Auxillary gas flow rate (Ar)	l min^−1^	0.85	0.85	0.85	0.80
Sample gas flow rate (Ar)	l min^−1^	0.96	0.96	0.96/0.88	0.78
N_2_ flow rate	ml min^−1^	3	4	10	4

**Extraction/pre-cell quadrupole**					
Extraction lens 2	V	−228.7	−287	−287	−202
Deflection entry	V	−35	−35	−35	−30
Angular deflection	V	−371	−397	−397	−250
Quadrupole entry	V	−150.4	−150	−150	−115
Quadrupole focus	V	−6.62	−6	−6	−19
Quadrupole exit	V	−60.32	−60	−60	−39
Quadrupole pole bias	V	−1	−1	−1	−2

**Collision cell**					
He flow rate	ml min^−1^	4.96	3.00	4.96–2.50	3.94
SF_6_ flow rate	ml min^−1^	0.045	0.025	0.045–0.015	0.075
Drag cell entry top	V	−23	−23	−23	
Drag cell entry bottom	V	−21	−21	−21	
Drag voltage exit	V	−85	−85	−(85–30)	
CCT bias	V	−2	−2	−2	−2
CCT exit 1	V	−7.3	−7.3	−7.3	−39.3
CCT exit 2	V	−40	−40	−40	

**Zoom optics**					
Focus	V	−2.2	−2.2	−2.2	
Dispersion	V	0	0	0	

**Source lenses (Neptune Plus™)**					
Focus	V	−729	−729	−729	
*X* Deflection	V	−0.6	−0.6	−0.6	
*Y* Deflection	V	0.4	0.4	0.4	
Source offset	V	−5	−5	−5	

Standard laser ablation conditions given in Table 3 were used for all *in situ* measurements unless otherwise stated. All analyses were made with the laser spot in fixed position on the sample or reference material. Each spot was ablated for 1000 or 600 laser shots. See ESI data Tables S2–S5, S7 and S10† for details of the laser conditions used in each experiment. To exclude periods of washout and signal instability after ablation was initiated, two cycles of data at the beginning and one at the end of the ablation were rejected from every analysis. Internal normalisation followed the same procedure as for solution measurements and no correction for isobaric ^87^RbF^+^ was made. The Rb/Sr ratio was determined from the measured ^85^Rb^+^ and ^88^Sr^+^ beams and externally-normalised to NIST SRM 610, whereas ^87^Sr/^86^Sr was externally-normalised to either NIST SRM 610 (ref. 36) or Te-1. During early method development, it was found that normalisation of ^87^Sr/^86^Sr to Te-1 always gave accurate results, whereas NIST SRM 610 did not reliably do so. The inaccuracies were eventually found to be associated with incomplete purging of the collision cell of oxygen used in prior, unrelated experiments, the presence of traces of oxygen giving rise to YO^+^, which interferes with ^86^SrF^+^. The presence of oxygen contamination in the collision gas was monitored by Sr isotopic analysis of NIST SRM 610 normalised to Te-1. Because NIST SRM 610 has a relatively high Y/Sr ratio (∼1) compared to Te-1, the inaccuracy in ^87^Sr/^86^Sr should be a sensitive indicator for the presence of unresolved oxygen-based interferences, such as ^89^Y^16^O^+^.

Tuning for *in situ* analyses maximised SrF^+^ sensitivity during line scan sampling of NIST SRM 610 under standard laser conditions. Additionally, for *in situ* dating, the Rb–Sr fractionation was reduced by decreasing the sample gas flow to the plasma and increasing the torch *Z* position from the sample cone until the measured ^85^Rb^+^/^88^Sr^+^ in NIST SRM 610, without SF_6_ in the collision cell, was ∼0.7. Further tuning of plasma conditions was required to reduce the differential Rb–Sr fractionation between NIST SRM 610 glass and sample minerals by increasing sampling depth to 15 mm (tune conditions using 15 mm *Z* torch depth and 0.96 l min^−1^ referred to as REF 1, see Table 3). Differential Rb–Sr fractionation between NIST SRM 610 glass and sample minerals was further reduced by decreasing the sample gas Ar flow to 0.88 l min^−1^ (tune conditions using 15 mm *Z* torch depth and 0.88 l min^−1^ referred to as REF 2, see Table 3). These changes are assumed to increase the plasma residence time of ablated aerosols resulting in more complete ionisation.^37^ Analyses using focus conditions REF 1 are referred to as dataset 1 and whereas datasets 2 and 3 were made using REF 2 conditions (see Table 4).

**Table 4:** ^85^Rb/^88^Sr correction factors and associated 95% CI, *s*_sys_, determined from the analysis of DG-1 for each dataset from Proteus and iCAP TQ™ Torch *Z* position and sample gas flow are reported for each dataset

Instrument	Dataset	Laser pulse count	*Z* Torch position (mm)	Sample gas (l min^−1^)	^85^Rb/^88^Sr correction factor	*s*_sys_
Proteus	1	1000	15	0.96	0.975	±0.004
Proteus	2	1000	15	0.88	1.004	±0.005
Proteus	3	600	15	0.88	0.996	±0.011
iCAP TQ		600	5	0.78	1.007	±0.057

The Rb–Sr instrumental fractionation difference between K-feldspar and the NIST SRM 610 glass was determined by means of measurements of secondary standard DG-1 prior to each analytical session. An isochron plot of the DG-1 data is made, with measured ^85^Rb/^88^Sr normalised to NIST SRM 610, and the slope compared to that corresponding to the published age of 285 Ma. The ratio of the observed to the expected slope gives a Rb–Sr correction factor, which is applied to all ^85^Rb/^88^Sr ratios measured in that session. The relative uncertainty of the DG-1 isochron slope, *i.e.* the relative uncertainty of the correction factor, *s*_sys_, is a systematic uncertainty and is added in quadrature to the (random) relative uncertainty of the isochron slope, *s*_rand_, for all samples measured in the session, to give the total relative uncertainty,1
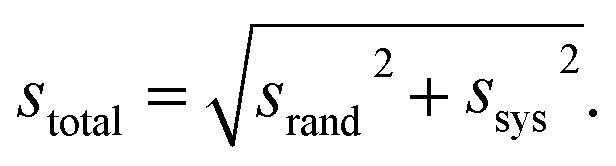


All slope and initial ^87^Sr/^86^Sr_i_ uncertainties are studentised 95% confidence intervals (95% CI) calculated in IsoplotR.^38^ This program uses the 2004 York algorithm^39^ to regress the data and calculate an age and uncertainty. The relative age and isochron slope uncertainties will be taken as being equal since the half-life of ^87^Rb is long compared to the isochron ages. A ^87^Rb decay constant of 1.3972 × 10^−11^ y^−1^ was used for all reported Rb–Sr ages^40^ and cited literature Rb–Sr ages have been recalculated using this decay constant to aid direct comparison.

#### 2.3.2 iCAP TQ™

For the analysis involving the iCAP TQ™ a SF_6_ (99.999% purity) reaction gas mixture was used. A separate He gas cylinder (99.9% purity) was connected into the reaction gas stream *via* its own mass flow controller allowing for independently adjustable SF_6_ and He partial pressures in the collision cell.

The first quadrupole, before the collision cell, was operated in ‘normal’ resolution, permitting the transmission of ions within a range of 10 u/e, see Table 2. The third quadrupole, after the collision cell, transmits a single mass with 0.7 u/e resolution. The iCAP TQ™ ion counter was operated in pulse counting mode.

The laser ablation and tuning conditions, for reduced elemental fractionation, are given in Table 3. Internal and external normalisation of isotope ratios follows the same treatment as for Proteus. The ^85^Rb^+^ and ^86^SrF^+^ beams were used to determine the ^87^Rb/^86^Sr ratio, rather than using the ^88^Sr^+^ beam, due to software limitations. The laser ablation duration was 600 shots with some data at the start and end rejected to match, approximately, the treatment of the Proteus data. A glass/feldspar bias correction factor for ^85^Rb/^88^Sr ratios was derived, as for Proteus, by best fitting measured DG-1 data to the literature age,^27^ which yielded a value of 1.007. The slope and initial ^87^Sr/^86^Sr_i_ uncertainties reported in this study for the analysis of DG-1 using the iCAP TQ™ are studentised 95% CI calculated in IsoplotR.^38^

## 3. Results and discussion

### 3.1 Rb–Sr chemical resolution

**Fig. 3:**
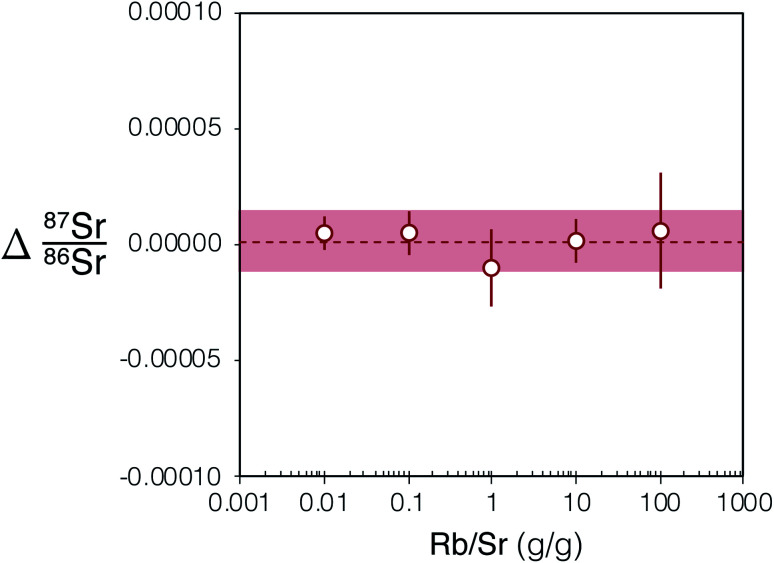
Plot showing Δ^87^Sr/^86^Sr = ^87^Sr/^86^Sr_measured_ − ^87^Sr/^86^Sr_reference_ of Rb-doped NIST SRM 987 after internal normalisation to ^86^Sr/^88^Sr = 0.1194 and external normalisation to an un-doped SRM 987 solution of the same concentration (0.05 μg g^−1^). 2SE uncertainties for each Rb-doped NIST SRM 987 solution shown using red bars (*n* = 5). The light red box shows the 2SD uncertainty for the five Rb-doped solution data plotted and the red dashed line is their mean. The Rb/Sr ratio reported is a mass ratio. For tabulated data used in this figure please refer to ESI Table S1.†

To assess the effectiveness of SF_6_ in chemically resolving the interference of ^87^Rb on ^87^Sr we conducted ^87^Sr/^86^Sr analyses of Rb-doped NIST SRM 987 solutions. The measurements of these solutions demonstrated that, despite analysing solutions with Rb/Sr ratios spanning 4 orders of magnitude (Rb/Sr = 0.01–100), the Δ^87^Sr/^86^Sr = ^87^Sr/^86^Sr_measured_ − ^87^Sr/^86^Sr_reference_ all remain within uncertainty of zero (Fig. 3). The mean Δ^87^Sr/^86^Sr for all measured Rb-doped NIST SRM 987 was 0.000001 ± 13, 2SD, *n* = 25 (Fig. 3). The results of this experiment demonstrate the efficiency of SF_6_ as a reaction gas to chemically completely resolve the interference of ^87^Rb on ^87^Sr in samples with a Rb/Sr as high as 100.

### 3.2 Pre-cell mass-filter

**Fig. 4:**
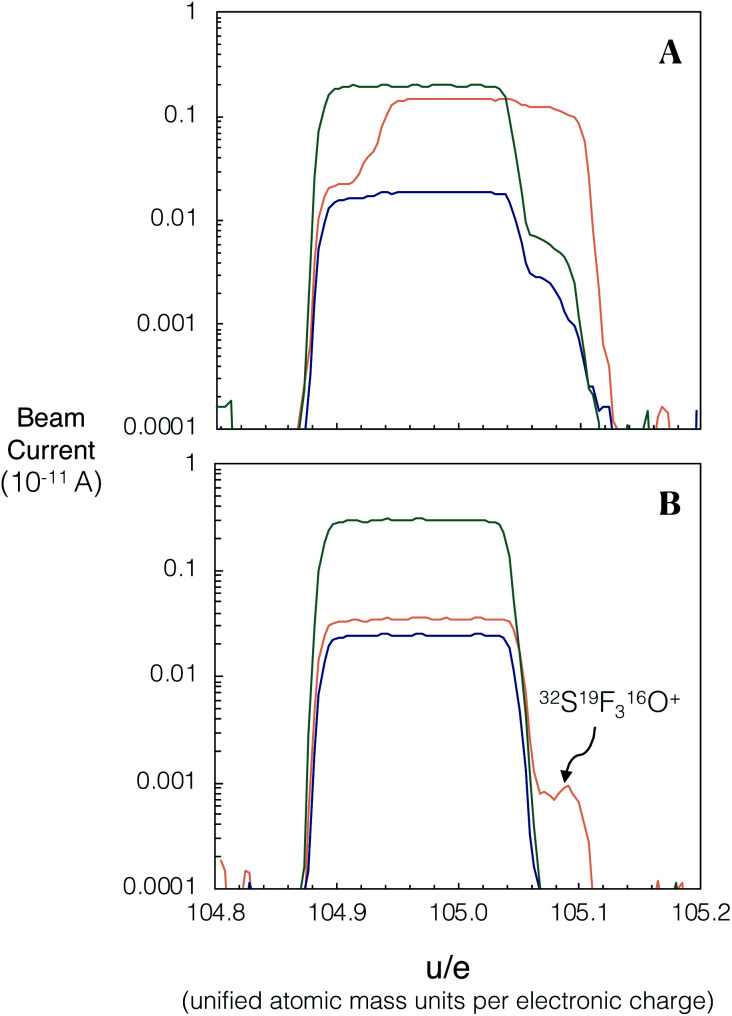
Peak scans of NIST SRM 610 using the low (Δ*M*/*M* ∼ 3000) resolution slit. The laser conditions used for both peak scans were a fluence of 6 J cm^−2^ and repetition rate of 10 Hz and a spot size of 110 μm. The orange, blue and green traces correspond to the central, high 1 and high 2 Faraday cups respectively (central = ^86^SrF^+^, high 1 = ^87^SrF^+^ and high 2 = ^88^SrF^+^). (A) Many interferences are produced from unwanted ion–gas reactions with matrix elements when the pre-cell mass-filter is operated in full quadrupole transmission mode. (B) Operating the pre-cell mass-filter in band-pass mode, transmitting 82–92 u/e, eliminates the majority of the isobars. The remaining interference on the high mass shoulder of the central Faraday cup is ^32^SF_3_^16^O^+^ which is sufficiently mass-resolved using a ‘low resolution’ aperture slit. Measurements of mass difference determined from a high resolution peak scan (Δ*M*/*M* ∼ 11 000) of ^86^SrF^+^ and the interference show a mass difference of 0.05 u/e, which is in agreement with the calculated mass difference expected for ^86^SrF^+^ and ^32^SF_3_^16^O^+^. The corresponding ^32^SF_3_^+^ beam intensity was ∼2 orders of magnitude larger than the ^32^SF_3_^16^O^+^ beam.

**Fig. 5:**
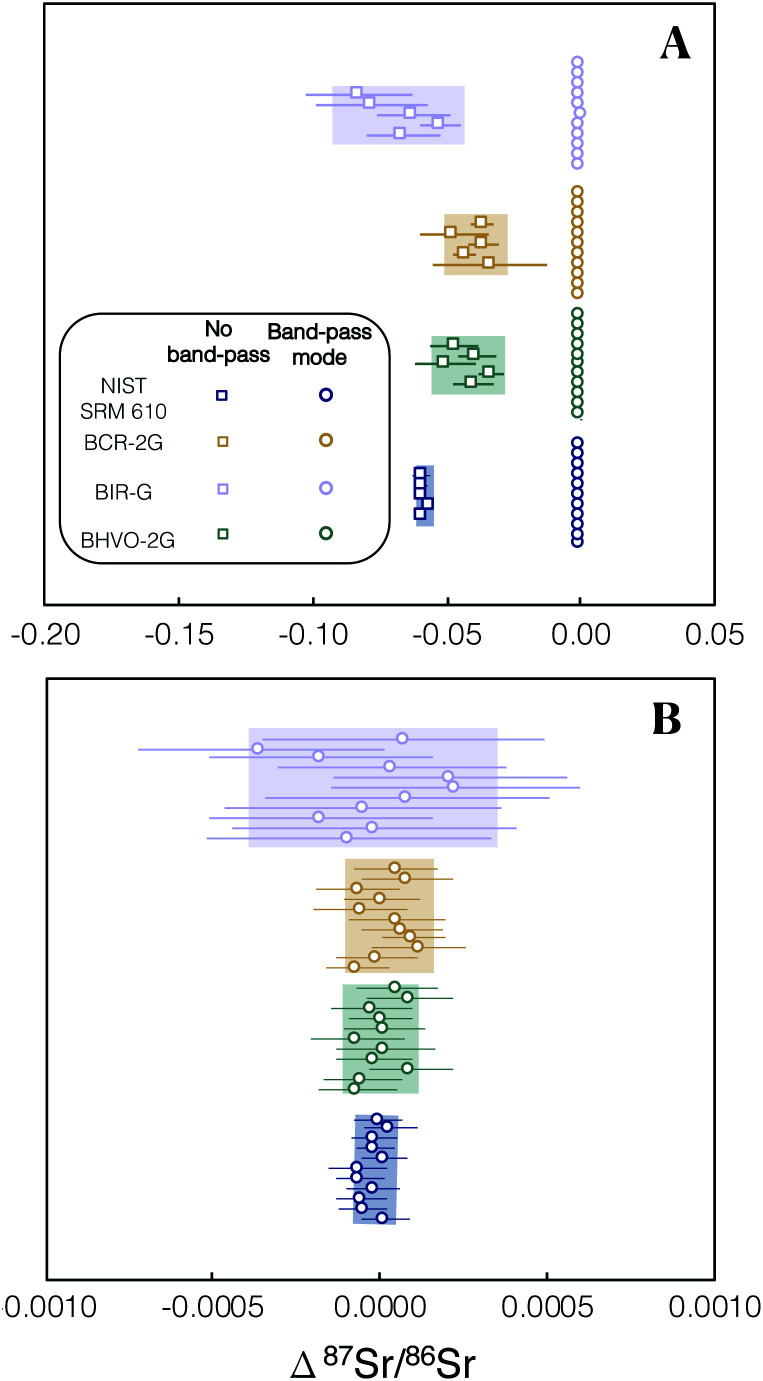
Plot showing the Δ^87^Sr/^86^Sr = ^87^Sr/^86^Sr_measured_ − ^87^Sr/^86^Sr_reference_ of several glass reference materials after internal normalisation and external normalised using Te-1. (A) Results are shown when using no pre-cell mass-filter by operating the quadrupole in full quadrupole transmission (FQT), square symbols, and when operating a 10 u/e band-pass (82–92 u/e), circle symbols. When using FQT all of the analyses are inaccurate (not within uncertainty of Δ^87^Sr/^86^Sr = 0) due to unresolved isobaric interferences. (B) All of the reference glasses provide accurate externally normalised ^87^Sr/^86^Sr ratios (within uncertainty of Δ^87^Sr/^86^Sr = 0) when the pre-cell mass-filter is set to the 10 u/e band-pass. For tabulated data used in this figure please refer to ESI Table S2.†

We investigated spectral interferences on the SrF^+^ spectrum during laser ablation. Fig. 4A shows an example of peak scans during *in situ* analysis of NIST SRM 610 without the use of the pre-cell mass-filter in band-pass mode. A variety of poly-atomic interferences are resolved on the high mass side of the peak scan when using a Neptune Plus™ low resolution slit on Proteus (*M*/Δ*M* ∼ 3000; Δ*M* is defined as 5–95% of full-height across a peak edge wherever Proteus or Neptune mass resolution is stated). To assess the accuracy of *in situ*^87^Sr/^86^Sr measurement without using an initial pre-collision cell mass-filter (same quadrupole conditions as Fig. 4A) and using a low resolution aperture slit, several mafic rock glasses (BCR-2G, BIR-G and BHVO-2G) and the synthetic SRM 610 were analysed and their measured ^87^Sr/^86^Sr ratios externally normalised using Te-1. The results of this experiment, show that without the pre-cell quadrupole operating in band-pass mode, isobaric interferences (insufficiently mass resolved with Δ*M*/*M* ∼ 3000 Fig. 4A) produce inaccurate measured ^87^Sr/^86^Sr for all of these reference materials when using Te-1 for external normalisation (Fig. 5A). However, with the pre-cell quadrupole operating in band-pass mode (transmission of 82–92 u/e) a significant reduction in poly-atomic interferences is observed (Fig. 4B) although some still persist. Molecular ion ^32^SF_3_^16^O^+^ is the largest remaining isobaric interference (Fig. 4B labelled on high mass shoulder of orange trace). The production of this interference is linked to the presence of ablated Zr and Y. When plasma derived Zr^+^ and Y^+^ ions are transmitted into the collision cell they react with SF_6_ to form SF_3_^+^ ions.^13^ We infer that SF_3_^+^ subsequently reacts with oxygen present as a contaminant in the reaction gas and any residual O_2_/He mix that remains in the shared SF_6_ pipework. Fortunately, ^32^SF_3_^16^O^+^ can be mass resolved from ^86^SrF^+^ sufficiently using a Neptune Plus™ low resolution slit (Δ*M*/*M* ∼ 2000 required to mass resolve ^86^SrF^+^ from ^32^SF_3_^16^O^+^). With this residual isobaric interference resolved and the pre-cell quadrupole operating in band-pass mode, the measured ^87^Sr/^86^Sr for all glass reference materials, when externally normalised using Te-1, were accurate (Δ^87^Sr/^86^Sr within uncertainty of zero, see Fig. 5B). This accuracy was achieved without any need for corrections for isobaric interferences. These results demonstrate that the removal of most ICP-derived atomic and polyatomic ions by the quadrupole mass-filter before they are able to enter the collision cell is crucial for the robustness of laser ablation analysis with Proteus, as for *in situ* analysis no prior purification can be conducted. Without this pre-cell mass-filter, the abundance of secondary interferences compromises the accuracy of the radiogenic Sr isotope ratios measured. The accurate measured ^87^Sr/^86^Sr for a variety of glass reference materials in band-pass mode, normalised to a reference, Te-1, with a very different composition (Fig. 5B), demonstrates the matrix robustness of this technique. This is particularly advantageous in constructing isochrons from a variety of different minerals, *e.g.* from feldspars to micas.

### 3.3 *In situ* Rb–Sr dating: Proteus

The Dartmoor granite sample DG-1 was used in this study to correct differential elemental fractionation of Rb–Sr between the reference glass (NIST SRM 610) and sample minerals, dominantly feldspar (see Section 2.3.1). The glass/feldspar bias correction factor for analytical sessions associated with each identified dataset (see Section 2.3.1) were within uncertainty, therefore all analysis of DG-1 for each dataset were combined to produce a single correction factor.

**Fig. 6:**
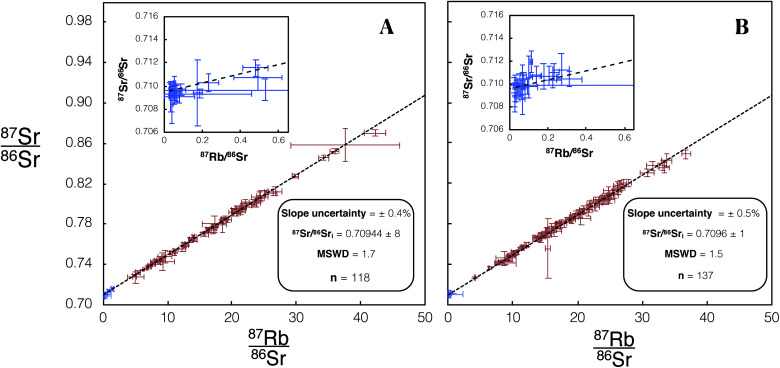
Isochron from *in situ* Rb–Sr analyses of DG-1 using Proteus. Blue/red data points are plagioclase/K-feldspar respectively. Error bars are 2SE individual spot uncertainties. (A) Includes data for DG-1 included in dataset 1 using REF 1 tune conditions. (B) Includes data for DG-1 included in dataset 2 using REF 2 tune conditions. Slope and ^87^Sr/^86^Sr_i_ uncertainties are 95% CI calculated in IsoplotR.^38^ For tabulated data used in the construction of these isochrons please refer to ESI Table S3.†

For *in situ* Rb–Sr dating, dataset 1 (Fig. 6A) 34 plagioclase and 84 K-feldspar laser spot analyses were carried out on DG-1. A glass/feldspar bias correction factor for measured ^85^Rb/^88^Sr in dataset 1 was determined to be 0.975 ± 4 from these analyses of DG-1 (Table 4). The analysis of DG-1 in dataset 2 includes 30 plagioclase and 107 K-feldspar spot analyses. The glass/feldspar bias correction factor determined from these analyses is 1.004 ± 5 (Table 4). The 95% CI associated with the slope of DG-1 isochrons for dataset 1 and 2 are 0.4% and 0.5% respectively. The initial ^87^Sr/^86^Sr calculated for DG-1 of 0.70944 ± 8 and 0.7096 ± 1 are within uncertainty of the value from a previous study of the same intrusion, 0.7094 ± 3,^27^ but considerably more precise.

**Fig. 7:**
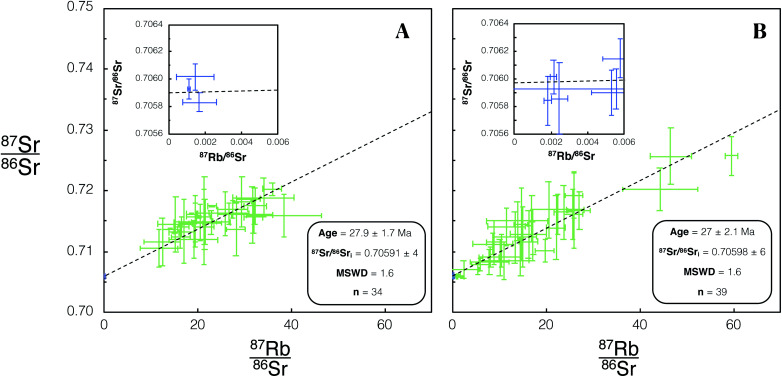
Isochron from *in situ* Rb–Sr analysis of FCT-1 using Proteus. Green/blue data points are mica/plagioclase respectively. Error bars are 2SE individual spot uncertainties. (A) Includes data for FCT-1 included in dataset 1 using REF 1 tune conditions. (B) Includes data for FCT-1 included in dataset 2 using REF 2 tune conditions. Age and ^87^Sr/^86^Sr_i_ uncertainties reported are 95% CI calculated in IsoplotR.^38^ For tabulated data used in the construction of these isochrons please refer to ESI Table S4.†

Once a glass/feldspar bias correction factor for measured ^85^Rb/^88^Sr was determined using DG-1, SG-1 and FCT-1 were then dated using identical tune conditions. For all samples dated in this study two age uncertainties are reported corresponding to Age × *s*_rand_ (reported in text and Table 5) and Age × *s*_total_ (reported in Table 5). For the dating of the Fish Canyon Tuff sample FCT-1, 3 plagioclase and 31 mica spot analyses were made using REF 1 focus conditions, 6 plagioclase and 33 mica spot analyses were collected using REF 2 conditions. The calculated ages for FCT-1 of 27.9 ± 1.7 Ma and 27.2 ± 2.1 Ma, from dataset 1 and 2 respectively (Fig. 7), are both within uncertainty of previous Rb–Sr dating studies of this eruption.^32^ The ^87^Sr/^86^Sr_i_ calculated for this sample of 0.70591 ± 4 and 0.70598 ± 4 are also within uncertainty of previously determined initial radiogenic Sr ratios for the Fish Canyon Tuff.^41^

**Table 5:** *In situ* Rb–Sr ages determined for all samples using Proteus for datasets 1 and 2.95% CI age uncertainties and total uncertainties, the latter being inclusive of the uncertainty of external normalisation of Rb–Sr inter-element fractionation, are reported as Age × *s*_rand_ and Age × *s*_total_. See Subsection 2.3.1 for details. A ^87^Rb decay constant of 1.3972 × 10^−11^ y^−1^ (ref. 40) was assumed for all reported Rb–Sr ages. Reference literature Rb–Sr ages have been recalculated using this decay constant to aid direct comparison. Reported reference age uncertainties are 2*σ*

Sample	Reference age, Ma	Dataset	Age, Ma (this study)	Age × *s*_rand_	Age × *s*_total_	^87^Sr/^86^Sr_i_ (this study)	95% CI
FCT-1	27.9 ± 0.2 (ref. 32)	1	27.9	±1.7	±1.7	0.70591	±0.00004
SG-1	400.3 ± 3 (ref. 28)	1	398.5	±2.5	±2.9	0.70755	±0.00004
SG1K1	400.3 ± 3 (ref. 28)	1	397.9	±6.2	±6.4	0.70740	±0.00017
FCT-1	27.9 ± 0.2 (ref. 32)	2	27.2	±2.1	±2.1	0.70598	±0.00006
SG-1	400.3 ± 3 (ref. 28)	2	397.2	±1.3	±2.3	0.70756	±0.00004
SG1K1	400.3 ± 3 (ref. 28)	2	397.2	±6.3	±6.6	0.70769	±0.00016

**Fig. 8:**
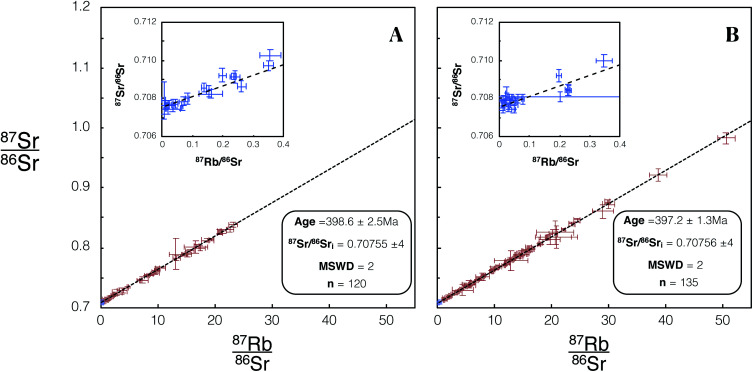
Isochron from *in situ* Rb–Sr analyses of SG-1 using Proteus. Blue/red data points are plagioclase/K-feldspar respectively. Error bars are 2SE individual spot uncertainties. (A) Includes data for SG-1 included in dataset 1 using REF 1 tune conditions. (B) Includes data for SG-1 included in dataset 2 using REF 2 tune conditions. Age and ^87^Sr/^86^Sr_i_ uncertainties reported are 95% CI calculated in IsoplotR.^38^ For tabulated data used in the construction of these isochrons please refer to ESI Table S5.†

We made 22 plagioclase and 98 K-feldspar spot analyses of the Shap granite sample SG-1 using REF 1 tuning conditions, and 26 plagioclase and 109 K-feldspar analyses using REF 2 conditions. These analyses of SG-1 yielded an age of 398.6 ± 2.5 Ma (Fig. 8A) and 397.2 ± 1.3 Ma (Fig. 8B). Initial ^87^Sr/^86^Sr_i_ ratios of 0.70755 ± 4 (Fig. 8A) and 0.70756 ± 4 (Fig. 8B) were calculated for SG-1. These values are in agreement with the age and initial ^87^Sr/^86^Sr calculated by previous TIMS studies of this intrusion^28,42^ and with comparable uncertainties (Table 5).

**Fig. 9:**
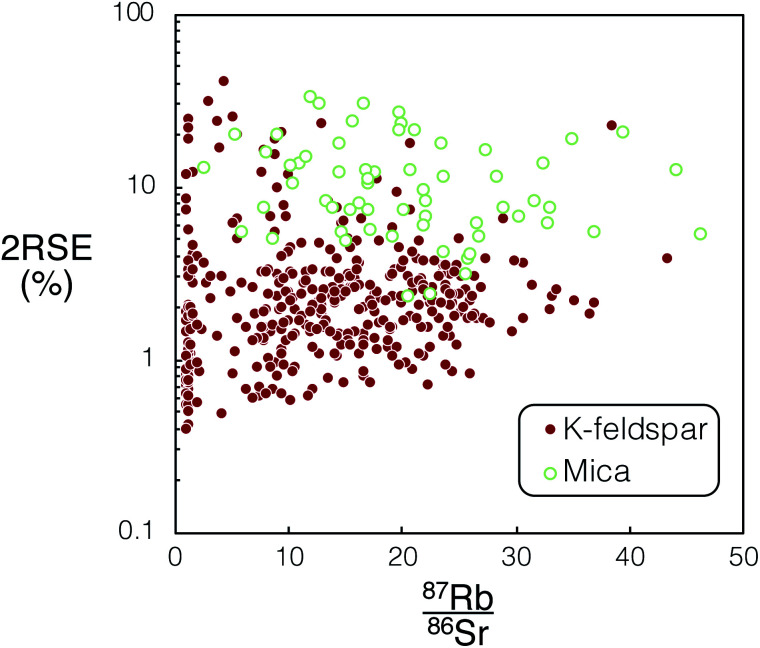
Comparison of calculated mean ^87^Rb/^86^Sr and associated 2RSE (%) *vs.* uncertainties for K-feldspar (DG-1 & SG-1) and mica (FCT-1) from Proteus dataset 1 and 2. For tabulated ^87^Rb/^86^Sr and associated uncertainties used in this figure please refer to ESI Tables S3–S5.†

Unlike the K-feldspar present in the DG-1 and SG-1, the sanidine in the Fish Canyon Tuff does not provide sufficient spread in ^87^Rb/^86^Sr for *in situ* Rb–Sr dating.^32^ Therefore a combination of mica and plagioclase spot analyses were used for the dating of this sample. There are large uncertainties for measured ^85^Rb/^88^Sr in the micas compared to the feldspar analysed in DG-1 and SG-1. We infer the increased ^85^Rb/^88^Sr uncertainty in micas may be caused by compositional heterogeneity exposed down-hole during ablation for this mineral (Fig. 9). The significantly less precise age determined for FCT-1 compared to SG-1 (Table 5) suggests *in situ* dating using K-feldspar and plagioclase, if possible, will produce higher precision ages.

We infer that the excellent agreement of our ages for the Shap granite and the Fish Canyon Tuff with prior TIMS analyses, when using DG-1 as a calibrant (Table 5), validates the ability of Proteus to perform accurate *in situ* Rb–Sr dating. The age resolution and uncertainty of ^87^Sr/^86^Sr_i_ ratios for SG-1 compared to TIMS demonstrate that relatively high precision dating (0.3%) can be achieved using the *in situ* dating of feldspar with this technique.

### 3.4 Proteus *vs.* CC-ICPMS/MS

**Fig. 10:**
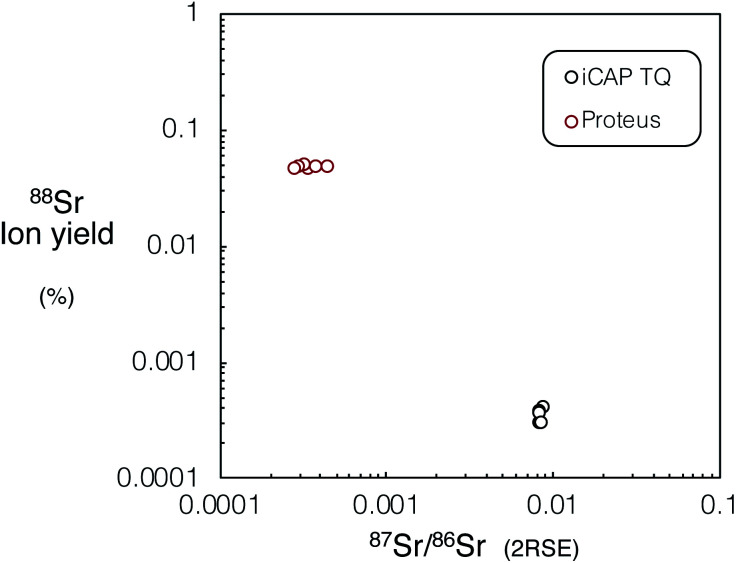
Mean ^88^Sr sample ion yield (atoms ablated per second/ions counted per second, assuming 9500 μm^3^ per second of ablated material) *vs.*^87^Sr/^86^Sr precision (2RSE) for Proteus and iCAP TQ™ for NIST SRM 610, using the same laser conditions (110 μm spot diameter, 10 Hz repetition rate, 6 J cm^−2^ fluence and 60 s integration time). For tabulated mean sample ion yields and associated ^87^Sr/^86^Sr ratio uncertainties please refer to ESI Table S6.†

We investigated the performance of Proteus for *in situ* Rb–Sr dating relative to experiments using the iCAP TQ™ and literature data for single collector Agilent 8800, which has been most widely used to date for *in situ* Rb–Sr dating. Tune conditions for both Proteus and iCAP TQ were optimised to reduce elemental fractionation, which is required for *in situ* dating. The use of the ‘cold plasma’ skimmer cone geometry for Proteus resulted in a factor of ∼4 improvement in Sr^+^ sensitivity when compared to the standard skimmer cone with ‘high sensitivity’ insert, which was used with the iCAP TQ™. Using the ARIS^18^ also resulted in a minimum factor of 2 improvement in sensitivity, compared to the standard 6 mm Analyte G2 sample introduction tubing for spot sizes 10–150 μm, and was used on both Proteus and the iCAP TQ™. The resulting sample ion yield (atoms ablated per second/ions counted per second, assuming 0.1 μm ablation depth per pulse) of ^88^SrF^+^ for Proteus was approximately two orders of magnitude greater than that achieved for the iCAP TQ™ (Fig. 10). However, we anticipate that using the cold plasma cone on the iCAP TQ™ should increase its sensitivity by a factor of ∼4, as for Proteus. We have calculated ion yields of the Agilent 8800 using reported intensities from ablation of NIST SRM 610 (∼1 million counts of ^88^SrF^+^ per second for a 50 μm diameter spot and 10 Hz spot ablation).^11^ Compared to this, Proteus appears to have approximately an order of magnitude better ion yield.

The ^87^Sr/^86^Sr ratios measured for NIST SRM 610 using Proteus were ∼25 times more precise than the iCAP TQ™. The best reported reproducibility achieved for *in situ*^87^Sr/^86^Sr spot analysis of NIST SRM 610 using single collector CC-ICPMS/MS when *in situ* Rb–Sr dating is 0.14% (ref. 17) (2RSD, *n* = 30). Comparatively, Proteus achieves a minimum reproducibility of 0.009% for ^87^Sr/^86^Sr measurements of NIST SRM 610 (2RSD, *n* = 11, see Section 3.2 and ESI S2†). This significant contrast in performance is achieved through a combination of increased sensitivity, coupled with the use of Faraday detectors and the ability to simultaneously collect all SrF^+^ isotopes on Proteus, which should result in better the age resolution of *in situ* Rb–Sr dating by Proteus compared to single collector instruments.

**Fig. 11:**
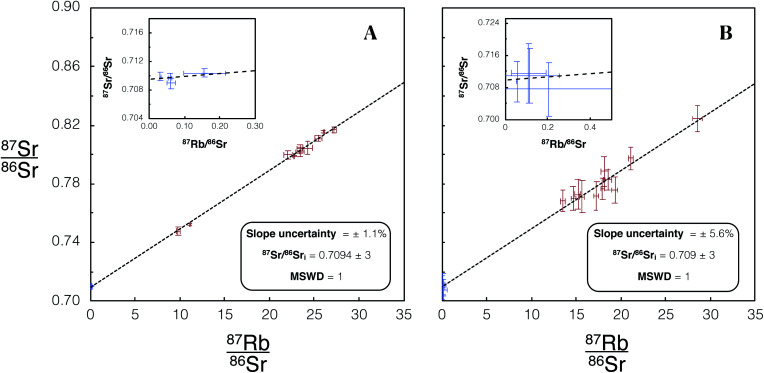
(A) Isochron from *in situ* Rb–Sr analysis of DG-1 using Proteus with an identical number of spot analyses and laser conditions to the analysis using the single collector iCAP TQ™, shown in (B). Blue/red data points are plagioclase/K-feldspar respectively. Error bars are 2SE individual spot uncertainties. Four plagioclase and 13 K-feldspar spot analyses were used to construct this isochron. Slope and ^87^Sr/^86^Sr_i_ uncertainties reported are 95% CI calculated in IsoplotR.^38^ For tabulated data used in the construction of these isochrons please refer to ESI Table S7.†

An empirical comparison of the performance of the iCAP TQ™ with Proteus for *in situ* dating was undertaken using the Dartmoor granite, comprising additional analyses of DG-1 using Proteus to those presented in Fig. 6. Identical laser conditions were used for analysis on both Proteus and iCAP TQ™. For Proteus and iCAP TQ™ instrument tune conditions see Tables 3 and 4 (Proteus dataset 3). The iCAP TQ™ yielded a slope uncertainty of ∼6% (Fig. 11B). The slope uncertainty is a factor ∼5 larger than that achieved using the companion Proteus isochron with the same number of spot analyses (Fig. 11A). The uncertainty of the calculated initial ^87^Sr/^86^Sr ratio when using the iCAP TQ™ is 0.4%, an order of magnitude larger than the uncertainty achieved using Proteus (Fig. 11). This direct comparison of Proteus with the iCAP TQ™ quantifies the advantages of CC-MC-ICPMS/MS over CC-ICPMS/MS in this new field of *in situ* Rb–Sr dating.

### 3.5 Theoretical age resolution

In order to generalise the effects of greater ion sensitivity and better precision of ^87^Sr/^86^Sr on the uncertainties of *in situ* Rb–Sr ages, we have made a simple model of age uncertainty. This model provides a useful indication of the contrasting age resolution possible by CC-ICPMS/MS (iCAP TQ™ and Agilent 8800) and Proteus.

We constructed model isochrons comprised of 11 points (hypothetical analyses). We generated isochrons of different ages and from points spanning different ranges of ^87^Rb/^86^Sr (0–1, 0–3, 0–30, 0–300). We assumed a fixed uncertainty for measured ^85^Rb/^88^Sr (2% 2RSD, from the mean for K-feldspar analyses using both Proteus and iCAP TQ™ presented in Fig. 11), as this is largely controlled by variable fractionation from laser ablation which should be similar for all instruments. All hypothetical analyses were assumed to have identical initial ^87^Sr/^86^Sr ratios and reference ^86^Sr/^88^Sr = 0.1194 (denoted *y*_0_ in equations below). We calculate ion intensities for each of the hypothetical analyses using their assigned Sr concentration, and assume the same relative ion yields as NIST SRM 610 (determined using mean sample ion yields reported in Section 3.4 and ESI Table S6†). Strontium concentrations and ^87^Rb/^86^Sr for the hypothetical analyses were assigned to approximately represent plagioclase (^87^Rb/^86^Sr = 0.001 and Sr concentration 500 μg g^−1^), K-feldspar (^87^Rb/^86^Sr = 0.1–30 and Sr concentration 50 μg g^−1^) and mica (^87^Rb/^86^Sr = 30–300 and Sr concentration 5 μg g^−1^). All model isochrons consisted of a single plagioclase and a set of K-feldspar analyses that evenly spanned the given range of ^87^Rb/^86^Sr, except for the model sample with ^87^Rb/^86^Sr 0–300, which was comprised of a plagioclase with ten hypothetical micas rather than K-feldspars. In addition to the generalised model, a specific comparison of DG-1 for Proteus and iCAP TQ™ was modelled. For this example the number of points and the ^87^Rb/^86^Sr used were identical to the analyses for each instrument presented in Fig. 11. The concentrations used for our hypothetical K-feldspar and plagioclase were also reduced to 30 μg g^−1^ and 100 μg g^−1^ respectively to more accurately represent the low Sr concentration in DG-1 feldspars. For further information regarding the ^87^Rb/^86^Sr and Sr concentrations used in this model see ESI Table S9.† An integration time of 60 seconds for SrF, Rb and Sr measurements was used in the model for all instruments.

The theoretical variances of ^88^Sr/^86^Sr (*y*) and un-normalised ^87^Sr/^86^Sr (*x*) are given by,2
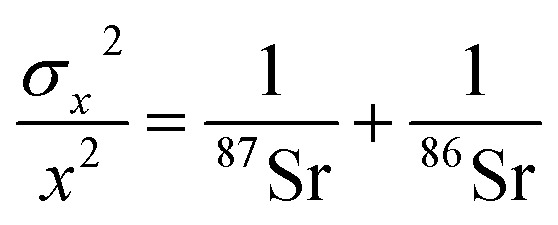
3
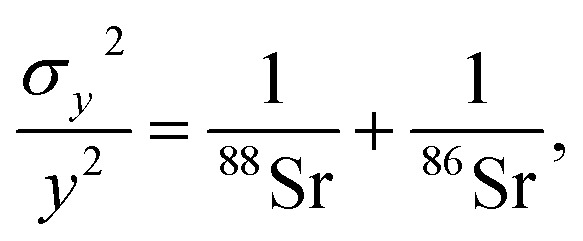
where ^86^Sr, ^87^Sr and ^88^Sr are the total ion fluxes collected during a hypothetical analysis. Hence, the co-variance is given by,4
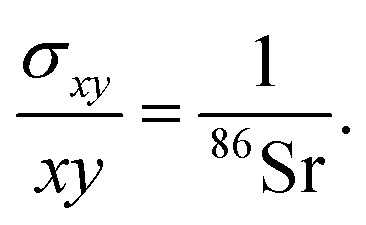


The normalised ^87^Sr/^86^Sr (*z*) is given by,

5 *z* = *x*e^−*P* ln(*y*/*y*_0_)^,

where6
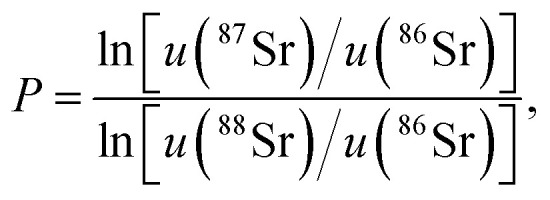
*y*_0_ is the reference ^88^Sr/^86^Sr ratio and *u* (^87^Sr) is the atomic mass of ^87^Sr *etc.* By first-order error propagation, the variance of *z* is given by,7
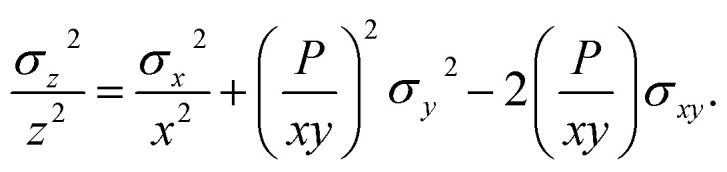


Additionally, to incorporate the effect of ion source instability and the performance of a multiplier collector system on the ultimate precision of ^87^Sr/^86^Sr measurements using single collector CC-ICPMS/MS instruments, additional noise (*n*) was added in quadrature to the relative uncertainty of *z* for the iCAP TQ™ and Agilent 8800 models, to give a total *z* uncertainty of,8
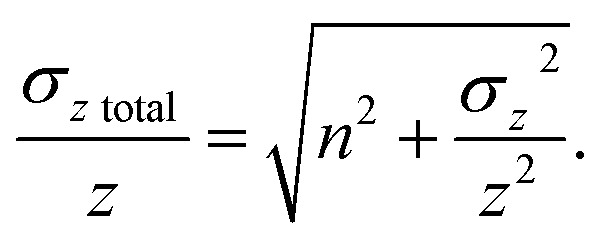


The value of *n* was chosen to result in a minimum 2*σ*_*z* total_/*z* of 0.14%, which equals the best reproducibility reported for *in situ* spot analysis of NIST SRM 610 using single collector CC-ICPMS/MS in previous *in situ* Rb–Sr dating studies.^17^ This uncertainty was comparable to the minimum ^87^Sr/^86^Sr uncertainty achieved using the iCAP TQ™. The minimum theoretical ^87^Sr/^86^Sr precision calculated for Proteus in the model did not exceed observed best reproducibility, therefore no additional uncertainty was applied to Proteus ^87^Sr/^86^Sr ratios.

**Fig. 12:**
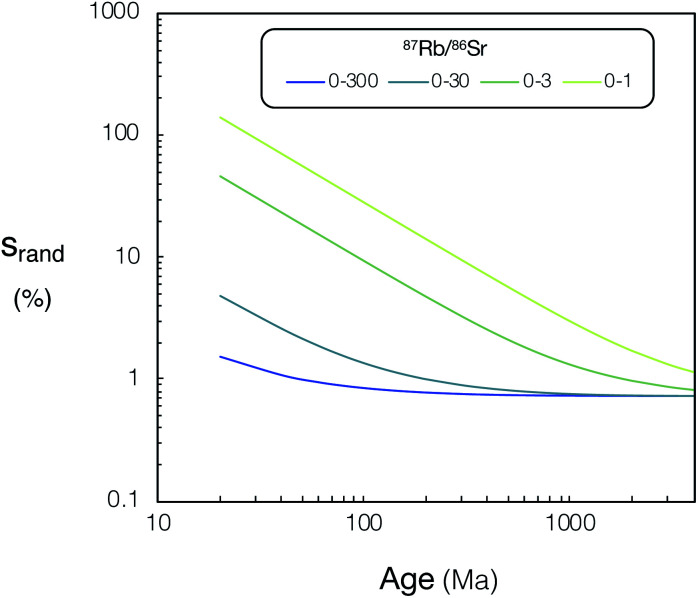
Relative uncertainties (*s*_rand_, %) of calculated ages for Proteus *vs.* age (20–4000 Ma) for various ^87^Rb/^86^Sr ranges, see text for details. For tabulated *s*_rand_, % and ^87^Rb/^86^Sr ranges used in this figure please refer to ESI Tables S8 and S9.†

**Fig. 13:**
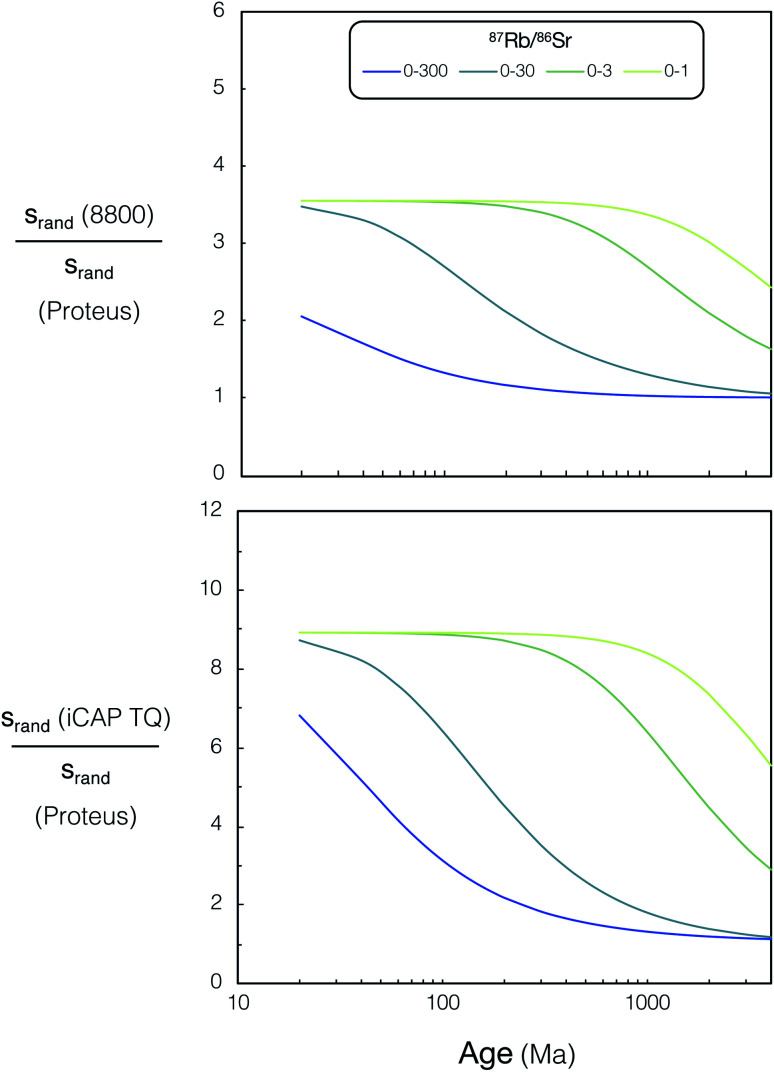
Relative age uncertainty ratio (Agilent 8800 QQQ)/(Proteus) (top) and (iCAP TQ™)/(Proteus) (bottom) *vs.* age (20–4000 Ma) for various ^87^Rb/^86^Sr ranges, see text for details. For tabulated uncertainties and ^87^Rb/^86^Sr ranges used in this figure please refer to ESI Tables S8 and S9.†

From the model uncertainties generated for the individual data points, uncertainties in isochron ages were calculated using IsoplotR.^38^ These values are shown for simulated Proteus data over a wide range of scenarios in (Fig. 12). A direct comparison of the modelled uncertainties of Proteus to that of the two CC-ICPMS/MS instruments is shown in (Fig. 13). Broadly, for younger samples, Proteus should produce more precise ages compared to both CC-ICPMS/MS instruments (Fig. 13). Also of note are the significantly lower age uncertainties calculated for samples with lower ^87^Rb/^86^Sr (Fig. 13). A 9 & 4-fold improvement in age uncertainty is predicted for Proteus compared to iCAP TQ™ and Agilent 8800 respectively, for a sample with an age of 20 Ma and ^87^Rb/^86^Sr range of 0–3. Conversely, modelled samples with ^87^Rb/^86^Sr that span to higher values (0–300) and older ages (>1 Ga), show smaller differences in uncertainty between Proteus and the two single collector CC-ICPMS/MS instruments (Fig. 13). The Proteus model gives comparable age uncertainties at significantly smaller spot sizes than both CC-ICPMS/MS instruments. For a sample with an age of 20 Ma and ^87^Rb/^86^Sr range of 0–30 Proteus achieves comparable relative age uncertainties to the Agilent 8800 and iCAP TQ™ (both using a 110 μm spot size) at spot sizes of ∼30 μm and ∼15 μm respectively. Our model also predicts a factor of 5 smaller age uncertainty for DG-1 measured by Proteus relative to the iCAP TQ™, which is in good agreement with the measured data reported in the previous section (Fig. 11).

Our results highlight the improvement in age resolution using Proteus, which now allows younger samples with lower ^87^Rb/^86^Sr to be viable for precise *in situ* dating.^6,14,15^ The modelled results also demonstrate the potential of Proteus to perform precise *in situ* dating at significantly higher spatial resolution than both CC-ICPMS/MS instruments, although the application of higher spatial resolution analysis of real samples using Proteus is beyond the scope of this study. Broadly our results illustrate the potential of Proteus to provide precise ages on Phanerozoic samples and make good chronological use of the widely occurring phase potassium feldspar, which has elevated, but not extremely high Rb/Sr.

### 3.6 Single grain *in situ* Rb–Sr dating

**Fig. 14:**
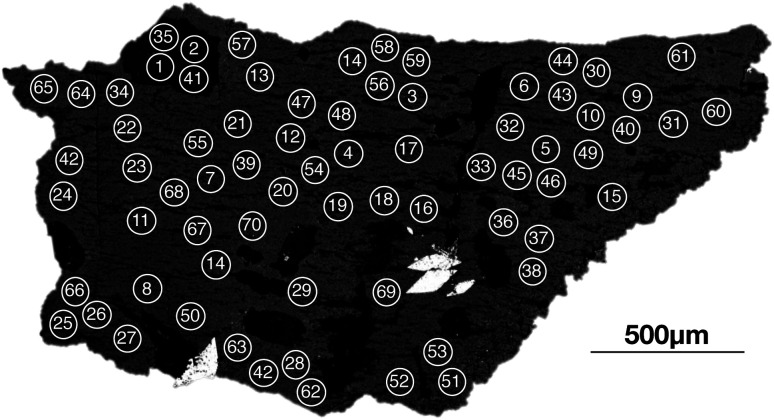
Back-scattered electron image of a single K-feldspar from the Shap granite (SG1K1) sample used for *in situ* single grain Rb–Sr dating on Proteus. These images were used to identify the location of laser spot positions within the K-feldspar grain (marked schematically). The image was specifically used to avoid quartz and titanite inclusions and target a plagioclase inclusion (identified with a Hitachi S-3500N™ scanning electron microscope at an accelerating voltage of 20 kV).

**Fig. 15:**
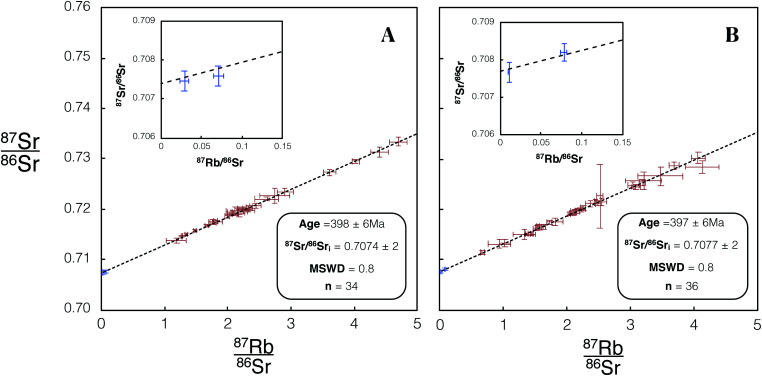
Isochron from *in situ* Rb–Sr analysis of a single K-feldspar SG1K1. 66 K-feldspar (red) and 4 plagioclase inclusion (blue) spot analyses were used in the construction of this isochron. (A) Includes data for SG1K1 included in dataset 1 using REF 1 tune conditions. (B) Includes data for SG1K1 included in dataset 2 using REF 2 tune conditions. Age and ^87^Sr/^86^Sr_i_ uncertainties reported are 95% CI calculated using IsoplotR.^38^ For tabulated data used in the construction of this isochron please refer to ESI Table S10.†

As an illustration of the unique capabilities of Proteus, we have constructed an internal isochron from *in situ* Rb–Sr analyses of a single K-feldspar grain from the Shap granite (SG1K1). Two age uncertainties are reported for each SG1K1 isochron, Age × *s*_rand_ (reported in text and Table 5) and Age × *s*_total_ (reported in Table 5). The presence of a plagioclase inclusion and primary, magmatic zoning in the potassium feldspar, provides a useful range in Rb/Sr. Using 70 individual spot analyses on this grain (Fig. 14) we calculate a single grain Rb–Sr of 398 ± 6 Ma for Proteus dataset 1 and 397 ± 6 Ma for dataset 2 (see Fig. 15). For the total age uncertainties of SG1K1 see Table 5. The ^87^Sr/^86^Sr_i_ calculated for SG1K1 was 0.7074 ± 2 for dataset 1 and 0.7077 ± 2 for dataset 2. The *in situ* Rb–Sr analysis of a single K-feldspar grain from the Shap granite produces ages and ^87^Sr/^86^Sr_i_ ratios that are within uncertainty of those calculated from the inter-mineral isochron (Fig. 8) and previous Rb–Sr analysis of the Shap granite^28^ (Table 5). The ability to obtain *in situ* Rb–Sr ages from single grains opens the door for a number of applications, including dating detrital K-feldspar preserved in sediments, providing a potentially powerful new tool in understanding the evolution of the continental crust using one of its major constituent mineral phases.

## 4. Conclusion

We demonstrate the ability of a novel CC-MC-ICPMS/MS instrument, Proteus, to yield accurate ages and initial ^87^Sr/^86^Sr ratios from *in situ* Rb–Sr analyses for typical, granitic crustal samples. Our results emphasise the importance of a pre-cell mass-filter to ensure the accuracy of *in situ* radiogenic Sr isotope ratio measurements, particularly when using poorly matrix-matched reference materials such as NIST SRM 610 and mafic rock glass standards. Compared to a single collector CC-ICPMS/MS instrument, similar to those used to pioneer *in situ* Rb–Sr analyses, the better ion-sensitivity and multicollection of Proteus yields a five-fold improvement in precision of an age for a Phanerozoic granite. A simple, counting statistical model indicates that Proteus offers the greatest advantages over single collector instruments for relatively young samples (<1 Ga) with low ^87^Rb/^86^Sr (<30). Hence, Proteus offers notable potential for dating commonly occurring rocks and constraining the evolution of the continents by facilitating dating of one of the most prevalent mineral phases, potassium feldspar. Notably we have *in situ* dated a single potassium feldspar from the Shap granite using an internal isochron, yielding an accurate age with a precision of ±1.5% (95% CI). Given the ease of sample preparation and the ability to date major mineral phases that comprise the continental crust, we suggest that multicollection, *in situ* Rb–Sr dating provides a valuable new tool in geochronology.

## Conflicts of interest

There are no conflicts to declare.

## Supplementary Material

JA-036-D1JA00006C-s001
