# Supplementary material for: In situ Rb–Sr dating by collision cell, multicollection inductively-coupled plasma mass-spectrometry with pre-cell mass-filter, (CC-MC-ICPMS/MS)
Source: J Anal At Spectrom. 2021 Apr 1;36(5):917–31. doi: 10.1039/d1ja00006c (PMC8115725; doi:10.1039/d1ja00006c)
Supplement: JA-036-D1JA00006C-s001 [file JA-036-D1JA00006C-s001.pdf]

Electronic Supplementary Material (ESI) for Journal of Analytical Atomic Spectrometry.
This journal is © The Royal Society of Chemistry 2021

Table S1: Data plotted in Figure 3

NIST SRM987 - Rb-doped 987

Rb/Sr	$^{87}Sr/^{86}Sr$	2SE	Δ $^{87}Sr/^{86}Sr$	n
0.01	0.710256	0.000007	0.000005	5
0.1	0.710256	0.000009	0.000005	5
1	0.710241	0.000017	-0.000010	5
10	0.710252	0.000009	0.000001	5
100	0.710257	0.000025	0.000006	5

Table S2: Data plotted in Figure 5

Instrument	Pre-cell quadrupole	Sample	$^{87}Sr/^{86}Sr$	2SE	Δ $^{87}Sr/^{86}Sr$	$^{87}Sr/^{86}Sr$ standard	Spot size μm	Repetiton rate (Hz)	Fluence (J/cm^2)	Pulse count
Proteus	No bandpass	NIST SRM610	0.651	0.001	-0.059	Te-1	110	10	6	600
Proteus	No bandpass	NIST SRM610	0.653	0.001	-0.056	Te-1	110	10	6	600
Proteus	No bandpass	NIST SRM610	0.650	0.001	-0.059	Te-1	110	10	6	600
Proteus	No bandpass	NIST SRM610	0.651	0.002	-0.059	Te-1	110	10	6	600
Proteus	No bandpass	NIST SRM610	0.650	0.003	-0.059	Te-1	110	10	6	600
Proteus	Bandpass mode	NIST SRM610	0.70971	0.00007	0.00002	Te-1	110	10	6	600
Proteus	Bandpass mode	NIST SRM610	0.70965	0.00007	-0.00005	Te-1	110	10	6	600
Proteus	Bandpass mode	NIST SRM610	0.70965	0.00008	-0.00005	Te-1	110	10	6	600
Proteus	Bandpass mode	NIST SRM610	0.70968	0.00008	-0.00002	Te-1	110	10	6	600
Proteus	Bandpass mode	NIST SRM610	0.70964	0.00007	-0.00006	Te-1	110	10	6	600
Proteus	Bandpass mode	NIST SRM610	0.70963	0.00008	-0.00007	Te-1	110	10	6	600
Proteus	Bandpass mode	NIST SRM610	0.70971	0.00007	0.00001	Te-1	110	10	6	600
Proteus	Bandpass mode	NIST SRM610	0.70968	0.00006	-0.00001	Te-1	110	10	6	600
Proteus	Bandpass mode	NIST SRM610	0.70968	0.00007	-0.00002	Te-1	110	10	6	600
Proteus	Bandpass mode	NIST SRM610	0.70973	0.00008	0.00003	Te-1	110	10	6	600
Proteus	Bandpass mode	NIST SRM610	0.70970	0.00007	0.00000	Te-1	110	10	6	600
Proteus	No bandpass	BHVO-2G	0.663	0.008	-0.040	Te-1	110	10	6	600
Proteus	No bandpass	BHVO-2G	0.670	0.004	-0.033	Te-1	110	10	6	600
Proteus	No bandpass	BHVO-2G	0.653	0.011	-0.051	Te-1	110	10	6	600
Proteus	No bandpass	BHVO-2G	0.665	0.007	-0.039	Te-1	110	10	6	600
Proteus	No bandpass	BHVO-2G	0.657	0.009	-0.047	Te-1	110	10	6	600
Proteus	Bandpass mode	BHVO-2G	0.70340	0.00012	-0.00007	Te-1	110	10	6	600
Proteus	Bandpass mode	BHVO-2G	0.70341	0.00012	-0.00006	Te-1	110	10	6	600
Proteus	Bandpass mode	BHVO-2G	0.70356	0.00012	0.00009	Te-1	110	10	6	600
Proteus	Bandpass mode	BHVO-2G	0.70345	0.00011	-0.00002	Te-1	110	10	6	600
Proteus	Bandpass mode	BHVO-2G	0.70348	0.00015	0.00002	Te-1	110	10	6	600
Proteus	Bandpass mode	BHVO-2G	0.70340	0.00014	-0.00007	Te-1	110	10	6	600
Proteus	Bandpass mode	BHVO-2G	0.70348	0.00012	0.00002	Te-1	110	10	6	600
Proteus	Bandpass mode	BHVO-2G	0.70347	0.00010	0.00000	Te-1	110	10	6	600
Proteus	Bandpass mode	BHVO-2G	0.70344	0.00012	-0.00003	Te-1	110	10	6	600
Proteus	Bandpass mode	BHVO-2G	0.70356	0.00013	0.00009	Te-1	110	10	6	600
Proteus	Bandpass mode	BHVO-2G	0.70352	0.00012	0.00005	Te-1	110	10	6	600
Proteus	No bandpass	BCR-2G	0.671	0.022	-0.034	Te-1	110	10	6	600
Proteus	No bandpass	BCR-2G	0.662	0.004	-0.043	Te-1	110	10	6	600
Proteus	No bandpass	BCR-2G	0.669	0.006	-0.036	Te-1	110	10	6	600
Proteus	No bandpass	BCR-2G	0.658	0.013	-0.047	Te-1	110	10	6	600
Proteus	No bandpass	BCR-2G	0.668	0.004	-0.037	Te-1	110	10	6	600
Proteus	Bandpass mode	BCR-2G	0.70493	0.00010	-0.00007	Te-1	110	10	6	600
Proteus	Bandpass mode	BCR-2G	0.70499	0.00012	-0.00001	Te-1	110	10	6	600
Proteus	Bandpass mode	BCR-2G	0.70512	0.00014	0.00012	Te-1	110	10	6	600
Proteus	Bandpass mode	BCR-2G	0.70510	0.00009	0.00010	Te-1	110	10	6	600
Proteus	Bandpass mode	BCR-2G	0.70507	0.00012	0.00007	Te-1	110	10	6	600
Proteus	Bandpass mode	BCR-2G	0.70505	0.00014	0.00005	Te-1	110	10	6	600
Proteus	Bandpass mode	BCR-2G	0.70494	0.00014	-0.00006	Te-1	110	10	6	600
Proteus	Bandpass mode	BCR-2G	0.70501	0.00011	0.00001	Te-1	110	10	6	600
Proteus	Bandpass mode	BCR-2G	0.70494	0.00012	-0.00006	Te-1	110	10	6	600
Proteus	Bandpass mode	BCR-2G	0.70508	0.00014	0.00008	Te-1	110	10	6	600
Proteus	Bandpass mode	BCR-2G	0.70505	0.00013	0.00005	Te-1	110	10	6	600
Proteus	No bandpass	BIR-G	0.637	0.014	-0.066	Te-1	110	10	6	600
Proteus	No bandpass	BIR-G	0.651	0.008	-0.052	Te-1	110	10	6	600
Proteus	No bandpass	BIR-G	0.640	0.014	-0.063	Te-1	110	10	6	600
Proteus	No bandpass	BIR-G	0.625	0.021	-0.078	Te-1	110	10	6	600
Proteus	No bandpass	BIR-G	0.620	0.020	-0.083	Te-1	110	10	6	600
Proteus	Bandpass mode	BIR-G	0.7030	0.0004	-0.0001	Te-1	110	10	6	600
Proteus	Bandpass mode	BIR-G	0.7031	0.0004	0.0000	Te-1	110	10	6	600
Proteus	Bandpass mode	BIR-G	0.7029	0.0003	-0.0002	Te-1	110	10	6	600
Proteus	Bandpass mode	BIR-G	0.7031	0.0004	-0.0001	Te-1	110	10	6	600
Proteus	Bandpass mode	BIR-G	0.7032	0.0004	0.0001	Te-1	110	10	6	600
Proteus	Bandpass mode	BIR-G	0.7033	0.0004	0.0002	Te-1	110	10	6	600
Proteus	Bandpass mode	BIR-G	0.7033	0.0003	0.0002	Te-1	110	10	6	600
Proteus	Bandpass mode	BIR-G	0.7031	0.0003	0.0000	Te-1	110	10	6	600
Proteus	Bandpass mode	BIR-G	0.7029	0.0003	-0.0002	Te-1	110	10	6	600
Proteus	Bandpass mode	BIR-G	0.7027	0.0004	-0.0004	Te-1	110	10	6	600
Proteus	Bandpass mode	BIR-G	0.7032	0.0004	0.0001	Te-1	110	10	6	600

Table S3: Data plotted in Figure 6

Sample: DG-1

Instrument	Dataset	Analysis Session	$^{87}Rb/^{86}Sr$	2SE	$^{87}Sr/^{86}Sr$	2SE	$^{87}Sr/^{86}Sr$ standard	$^{87}Rb/^{86}Sr$ correction factor	Spot diameter μm	Repetiton rate (Hz)	Fluence (J/cm^2)	Pulse count	Torch Depth (mm)	Sample gas (l/min)
Proteus	Dataset 1	Session 1	0.04647	0.00181	0.70909	0.00037	Te-1	0.975	110	10	6	1000	15	0.96
Proteus	Dataset 1	Session 1	0.18930	0.26987	0.70935	0.00039	Te-1	0.975	110	10	6	1000	15	0.96
Proteus	Dataset 1	Session 1	0.03941	0.00319	0.70950	0.00041	Te-1	0.975	110	10	6	1000	15	0.96
Proteus	Dataset 1	Session 1	12.34330	0.58973	0.75551	0.00305	Te-1	0.975	110	10	6	1000	15	0.96
Proteus	Dataset 1	Session 1	12.46032	0.27433	0.75965	0.00192	Te-1	0.975	110	10	6	1000	15	0.96
Proteus	Dataset 1	Session 1	0.89432	0.17388	0.71373	0.00110	Te-1	0.975	110	10	6	1000	15	0.96
Proteus	Dataset 1	Session 1	16.27929	0.33053	0.77466	0.00234	Te-1	0.975	110	10	6	1000	15	0.96
Proteus	Dataset 1	Session 1	21.84122	0.46676	0.79771	0.00258	Te-1	0.975	110	10	6	1000	15	0.96
Proteus	Dataset 1	Session 1	12.93642	0.32691	0.75956	0.00127	Te-1	0.975	110	10	6	1000	15	0.96
Proteus	Dataset 1	Session 1	12.63181	0.26165	0.75880	0.00201	Te-1	0.975	110	10	6	1000	15	0.96
Proteus	Dataset 1	Session 1	0.49105	0.12661	0.71073	0.00106	Te-1	0.975	110	10	6	1000	15	0.96
Proteus	Dataset 1	Session 1	0.07991	0.07862	0.70909	0.00059	Te-1	0.975	110	10	6	1000	15	0.96
Proteus	Dataset 1	Session 1	0.23147	0.05620	0.71030	0.00077	Te-1	0.975	110	10	6	1000	15	0.96
Proteus	Dataset 1	Session 1	15.77071	0.57979	0.77000	0.00263	Te-1	0.975	110	10	6	1000	15	0.96
Proteus	Dataset 1	Session 1	8.85642	0.88231	0.74384	0.00349	Te-1	0.975	110	10	6	1000	15	0.96
Proteus	Dataset 1	Session 1	5.05375	1.27650	0.72743	0.00552	Te-1	0.975	110	10	6	1000	15	0.96
Proteus	Dataset 1	Session 1	22.09719	0.52567	0.80182	0.00317	Te-1	0.975	110	10	6	1000	15	0.96
Proteus	Dataset 1	Session 1	37.58319	8.40550	0.85815	0.01617	Te-1	0.975	110	10	6	1000	15	0.96
Proteus	Dataset 1	Session 1	8.53530	0.47269	0.74203	0.00157	Te-1	0.975	110	10	6	1000	15	0.96
Proteus	Dataset 1	Session 1	14.17142	0.39995	0.76457	0.00185	Te-1	0.975	110	10	6	1000	15	0.96
Proteus	Dataset 1	Session 2	0.04023	0.00584	0.70840	0.00156	Te-1	0.975	110	10	6	1000	15	0.96
Proteus	Dataset 1	Session 2	0.03656	0.00658	0.70899	0.00117	Te-1	0.975	110	10	6	1000	15	0.96
Proteus	Dataset 1	Session 2	18.49334	0.56717	0.77910	0.00697	Te-1	0.975	110	10	6	1000	15	0.96
Proteus	Dataset 1	Session 2	0.17587	0.02225	0.70942	0.00279	Te-1	0.975	110	10	6	1000	15	0.96
Proteus	Dataset 1	Session 2	18.68593	0.43252	0.78341	0.00401	Te-1	0.975	110	10	6	1000	15	0.96
Proteus	Dataset 1	Session 2	12.67146	0.23486	0.75884	0.00265	Te-1	0.975	110	10	6	1000	15	0.96
Proteus	Dataset 1	Session 2	17.10492	0.41165	0.77552	0.00489	Te-1	0.975	110	10	6	1000	15	0.96
Proteus	Dataset 1	Session 2	11.02758	0.30500	0.75191	0.00286	Te-1	0.975	110	10	6	1000	15	0.96
Proteus	Dataset 1	Session 2	0.05449	0.00689	0.70900	0.00052	Te-1	0.975	110	10	6	1000	15	0.96
Proteus	Dataset 1	Session 2	0.08850	0.02489	0.70971	0.00049	Te-1	0.975	110	10	6	1000	15	0.96
Proteus	Dataset 1	Session 2	0.04946	0.00525	0.70955	0.00050	Te-1	0.975	110	10	6	1000	15	0.96
Proteus	Dataset 1	Session 2	17.39552	0.39494	0.77771	0.00301	Te-1	0.975	110	10	6	1000	15	0.96
Proteus	Dataset 1	Session 2	17.06159	0.22573	0.77818	0.00295	Te-1	0.975	110	10	6	1000	15	0.96
Proteus	Dataset 1	Session 2	9.63567	1.12243	0.74770	0.00622	Te-1	0.975	110	10	6	1000	15	0.96
Proteus	Dataset 1	Session 2	20.08128	0.61631	0.79182	0.00409	Te-1	0.975	110	10	6	1000	15	0.96
Proteus	Dataset 1	Session 2	16.83919	0.37203	0.77433	0.00304	Te-1	0.975	110	10	6	1000	15	0.96
Proteus	Dataset 1	Session 2	26.73876	1.03434	0.81258	0.00434	Te-1	0.975	110	10	6	1000	15	0.96
Proteus	Dataset 1	Session 2	13.57147	0.35969	0.76420	0.00244	Te-1	0.975	110	10	6	1000	15	0.96

Proteus	Dataset 1	Session 3	0.04667	0.01304	0.70971	0.00026	NIST SRM610	0.975	110	10	6	1000	15	0.96
Proteus	Dataset 1	Session 3	0.03400	0.00750	0.70955	0.00027	NIST SRM610	0.975	110	10	6	1000	15	0.96
Proteus	Dataset 1	Session 3	18.91192	0.63519	0.78547	0.00264	NIST SRM610	0.975	110	10	6	1000	15	0.96
Proteus	Dataset 1	Session 3	8.67859	0.08376	0.74479	0.00075	NIST SRM610	0.975	110	10	6	1000	15	0.96
Proteus	Dataset 1	Session 3	20.99975	1.01399	0.79336	0.00333	NIST SRM610	0.975	110	10	6	1000	15	0.96
Proteus	Dataset 1	Session 3	7.71670	0.09835	0.74108	0.00077	NIST SRM610	0.975	110	10	6	1000	15	0.96
Proteus	Dataset 1	Session 3	42.22959	1.62741	0.87046	0.00342	NIST SRM610	0.975	110	10	6	1000	15	0.96
Proteus	Dataset 1	Session 3	21.75644	0.33921	0.79484	0.00134	NIST SRM610	0.975	110	10	6	1000	15	0.96
Proteus	Dataset 1	Session 3	0.03557	0.00096	0.70965	0.00049	NIST SRM610	0.975	110	10	6	1000	15	0.96
Proteus	Dataset 1	Session 3	0.05888	0.00879	0.70965	0.00045	NIST SRM610	0.975	110	10	6	1000	15	0.96
Proteus	Dataset 1	Session 3	0.03565	0.00051	0.70978	0.00065	NIST SRM610	0.975	110	10	6	1000	15	0.96
Proteus	Dataset 1	Session 3	8.44606	0.05755	0.74404	0.00076	NIST SRM610	0.975	110	10	6	1000	15	0.96
Proteus	Dataset 1	Session 3	21.10882	0.61636	0.79486	0.00287	NIST SRM610	0.975	110	10	6	1000	15	0.96
Proteus	Dataset 1	Session 3	7.27706	0.04486	0.73887	0.00095	NIST SRM610	0.975	110	10	6	1000	15	0.96
Proteus	Dataset 1	Session 3	21.86816	0.26140	0.79524	0.00111	NIST SRM610	0.975	110	10	6	1000	15	0.96
Proteus	Dataset 1	Session 3	5.33294	0.34415	0.73201	0.00163	NIST SRM610	0.975	110	10	6	1000	15	0.96
Proteus	Dataset 1	Session 3	0.52860	0.72489	0.70966	0.00093	NIST SRM610	0.975	110	10	6	1000	15	0.96
Proteus	Dataset 1	Session 3	0.48089	0.06591	0.71157	0.00071	NIST SRM610	0.975	110	10	6	1000	15	0.96
Proteus	Dataset 1	Session 3	0.06345	0.01628	0.70909	0.00058	NIST SRM610	0.975	110	10	6	1000	15	0.96
Proteus	Dataset 1	Session 3	13.01387	0.22061	0.76009	0.00128	NIST SRM610	0.975	110	10	6	1000	15	0.96
Proteus	Dataset 1	Session 3	7.95771	0.08028	0.74099	0.00060	NIST SRM610	0.975	110	10	6	1000	15	0.96
Proteus	Dataset 1	Session 3	9.17823	0.10410	0.74493	0.00104	NIST SRM610	0.975	110	10	6	1000	15	0.96
Proteus	Dataset 1	Session 3	9.04757	0.17688	0.74457	0.00109	NIST SRM610	0.975	110	10	6	1000	15	0.96
Proteus	Dataset 1	Session 3	0.03383	0.00326	0.70964	0.00025	NIST SRM610	0.975	110	10	6	1000	15	0.96
Proteus	Dataset 1	Session 3	0.04437	0.00895	0.70999	0.00021	NIST SRM610	0.975	110	10	6	1000	15	0.96
Proteus	Dataset 1	Session 3	9.20290	0.05866	0.74729	0.00067	NIST SRM610	0.975	110	10	6	1000	15	0.96
Proteus	Dataset 1	Session 3	21.25398	0.22080	0.79444	0.00110	NIST SRM610	0.975	110	10	6	1000	15	0.96
Proteus	Dataset 1	Session 3	23.80091	0.20062	0.80337	0.00137	NIST SRM610	0.975	110	10	6	1000	15	0.96
Proteus	Dataset 1	Session 3	7.06919	0.06487	0.73814	0.00062	NIST SRM610	0.975	110	10	6	1000	15	0.96
Proteus	Dataset 1	Session 3	29.81628	0.52335	0.82753	0.00228	NIST SRM610	0.975	110	10	6	1000	15	0.96
Proteus	Dataset 1	Session 3	11.18680	0.35758	0.75361	0.00141	NIST SRM610	0.975	110	10	6	1000	15	0.96
Proteus	Dataset 1	Session 3	0.04013	0.00140	0.70942	0.00029	NIST SRM610	0.975	110	10	6	1000	15	0.96
Proteus	Dataset 1	Session 3	26.36969	0.68527	0.81093	0.00323	NIST SRM610	0.975	110	10	6	1000	15	0.96
Proteus	Dataset 1	Session 3	9.53591	0.12233	0.74753	0.00072	NIST SRM610	0.975	110	10	6	1000	15	0.96
Proteus	Dataset 1	Session 3	20.73994	0.80576	0.79421	0.00382	NIST SRM610	0.975	110	10	6	1000	15	0.96
Proteus	Dataset 1	Session 3	35.93434	0.76736	0.85195	0.00277	NIST SRM610	0.975	110	10	6	1000	15	0.96
Proteus	Dataset 1	Session 3	6.16185	0.04169	0.73499	0.00057	NIST SRM610	0.975	110	10	6	1000	15	0.96
Proteus	Dataset 1	Session 3	34.28246	0.74489	0.84558	0.00229	NIST SRM610	0.975	110	10	6	1000	15	0.96
Proteus	Dataset 1	Session 3	18.30607	0.21374	0.78295	0.00113	NIST SRM610	0.975	110	10	6	1000	15	0.96
Proteus	Dataset 1	Session 3	0.03781	0.00294	0.70938	0.00038	NIST SRM610	0.975	110	10	6	1000	15	0.96
Proteus	Dataset 1	Session 3	0.05502	0.00265	0.70921	0.00048	NIST SRM610	0.975	110	10	6	1000	15	0.96
Proteus	Dataset 1	Session 3	19.34270	0.65191	0.78446	0.00298	NIST SRM610	0.975	110	10	6	1000	15	0.96
Proteus	Dataset 1	Session 3	7.33707	0.05048	0.73823	0.00052	NIST SRM610	0.975	110	10	6	1000	15	0.96
Proteus	Dataset 1	Session 3	23.21784	0.62390	0.80184	0.00225	NIST SRM610	0.975	110	10	6	1000	15	0.96
Proteus	Dataset 1	Session 3	6.78037	0.04103	0.73650	0.00055	NIST SRM610	0.975	110	10	6	1000	15	0.96
Proteus	Dataset 1	Session 3	23.55066	0.32247	0.80001	0.00140	NIST SRM610	0.975	110	10	6	1000	15	0.96
Proteus	Dataset 1	Session 4	21.36103	0.79135	0.79313	0.00386	Te-1	0.975	110	10	6	1000	15	0.96
Proteus	Dataset 1	Session 4	8.69609	0.27133	0.74375	0.00110	Te-1	0.975	110	10	6	1000	15	0.96
Proteus	Dataset 1	Session 4	10.38568	0.14742	0.75185	0.00144	Te-1	0.975	110	10	6	1000	15	0.96
Proteus	Dataset 1	Session 4	25.30093	0.21148	0.81138	0.00238	Te-1	0.975	110	10	6	1000	15	0.96
Proteus	Dataset 1	Session 4	8.20372	0.54695	0.74258	0.00230	Te-1	0.975	110	10	6	1000	15	0.96
Proteus	Dataset 1	Session 4	22.63651	0.39423	0.79888	0.00233	Te-1	0.975	110	10	6	1000	15	0.96
Proteus	Dataset 1	Session 4	9.14653	0.13502	0.74549	0.00107	Te-1	0.975	110	10	6	1000	15	0.96
Proteus	Dataset 1	Session 4	9.23077	1.90195	0.74311	0.00857	Te-1	0.975	110	10	6	1000	15	0.96
Proteus	Dataset 1	Session 4	0.03228	0.00360	0.70978	0.00056	Te-1	0.975	110	10	6	1000	15	0.96
Proteus	Dataset 1	Session 4	17.13764	0.32337	0.77696	0.00246	Te-1	0.975	110	10	6	1000	15	0.96
Proteus	Dataset 1	Session 5	0.03400	0.00170	0.70979	0.00050	NIST SRM610	0.975	110	10	6	1000	15	0.96
Proteus	Dataset 1	Session 5	0.06351	0.03091	0.70957	0.00054	NIST SRM610	0.975	110	10	6	1000	15	0.96
Proteus	Dataset 1	Session 5	24.53127	0.74301	0.80818	0.00510	NIST SRM610	0.975	110	10	6	1000	15	0.96
Proteus	Dataset 1	Session 5	24.58162	0.46269	0.80584	0.00251	NIST SRM610	0.975	110	10	6	1000	15	0.96
Proteus	Dataset 1	Session 5	17.28797	1.93832	0.78001	0.00809	NIST SRM610	0.975	110	10	6	1000	15	0.96
Proteus	Dataset 1	Session 5	16.14368	0.22533	0.77426	0.00122	NIST SRM610	0.975	110	10	6	1000	15	0.96
Proteus	Dataset 1	Session 5	0.04112	0.00587	0.70959	0.00058	NIST SRM610	0.975	110	10	6	1000	15	0.96
Proteus	Dataset 1	Session 5	0.02825	0.00063	0.70972	0.00061	NIST SRM610	0.975	110	10	6	1000	15	0.96
Proteus	Dataset 1	Session 5	4.89699	0.29817	0.72861	0.00150	NIST SRM610	0.975	110	10	6	1000	15	0.96
Proteus	Dataset 1	Session 5	17.44157	0.28813	0.77788	0.00141	NIST SRM610	0.975	110	10	6	1000	15	0.96
Proteus	Dataset 1	Session 5	23.85963	0.50559	0.80099	0.00420	NIST SRM610	0.975	110	10	6	1000	15	0.96
Proteus	Dataset 1	Session 5	15.51928	0.22463	0.77042	0.00116	NIST SRM610	0.975	110	10	6	1000	15	0.96
Proteus	Dataset 1	Session 6	0.06808	0.01136	0.70940	0.00080	Te-1	0.975	110	10	6	1000	15	0.96
Proteus	Dataset 1	Session 6	21.65363	0.15597	0.79791	0.00254	Te-1	0.975	110	10	6	1000	15	0.96
Proteus	Dataset 1	Session 6	13.66038	0.28719	0.76488	0.00108	Te-1	0.975	110	10	6	1000	15	0.96
Proteus	Dataset 1	Session 6	20.63731	0.57030	0.79123	0.00308	Te-1	0.975	110	10	6	1000	15	0.96
Proteus	Dataset 1	Session 6	20.37970	0.50168	0.79118	0.00269	Te-1	0.975	110	10	6	1000	15	0.96
Proteus	Dataset 1	Session 6	23.03552	0.31540	0.79959	0.00216	Te-1	0.975	110	10	6	1000	15	0.96
Proteus	Dataset 1	Session 6	0.05217	0.00837	0.71026	0.00056	Te-1	0.975	110	10	6	1000	15	0.96
Proteus	Dataset 1	Session 6	20.42554	0.18142	0.79333	0.00167	Te-1	0.975	110	10	6	1000	15	0.96
Proteus	Dataset 1	Session 6	1.48573	0.18240	0.71587	0.00141	Te-1	0.975	110	10	6	1000	15	0.96
Proteus	Dataset 1	Session 6	24.30470	1.23054	0.80696	0.00483	Te-1	0.975	110	10	6	1000	15	0.96
Proteus	Dataset 1	Session 6	14.09719	0.19604	0.76737	0.00147	Te-1	0.975	110	10	6	1000	15	0.96
Proteus	Dataset 1	Session 6	21.65698	0.49759	0.79529	0.00291	Te-1	0.975	110	10	6	1000	15	0.96
Proteus	Dataset 2	Session 7	0.04302	0.00286	0.70972	0.00068	Te-1	1.004	110	10	6	1000	15	0.88
Proteus	Dataset 2	Session 7	11.44827	0.32246	0.75599	0.00231	Te-1	1.004	110	10	6	1000	15	0.88
Proteus	Dataset 2	Session 7	20.79770	0.86283	0.79139	0.00310	Te-1	1.004	110	10	6	1000	15	0.88
Proteus	Dataset 2	Session 7	19.11298	0.28861	0.78654	0.00227	Te-1	1.004	110	10	6	1000	15	0.88
Proteus	Dataset 2	Session 7	24.84638	0.90635	0.80327	0.00357	Te-1	1.004	110	10	6	1000	15	0.88
Proteus	Dataset 2	Session 7	0.11233	0.00884	0.71194	0.00095	Te-1	1.004	110	10	6	1000	15	0.88
Proteus	Dataset 2	Session 7	0.22982	0.02617	0.71103	0.00074	Te-1	1.004	110	10	6	1000	15	0.88
Proteus	Dataset 2	Session 7	26.64000	0.45793	0.81633	0.00324	Te-1	1.004	110	10	6	1000	15	0.88
Proteus	Dataset 2	Session 7	23.92240	0.60154	0.80501	0.00301	Te-1	1.004	110	10	6	1000	15	0.88
Proteus	Dataset 2	Session 7	4.12682	0.12545	0.72651	0.00094	Te-1	1.004	110	10	6	1000	15	0.88
Proteus	Dataset 2	Session 7	14.67812	0.18539	0.76901	0.00193	Te-1	1.004	110	10	6	1000	15	0.88
Proteus	Dataset 2	Session 7	13.41815	0.43472	0.76556	0.00287	Te-1	1.004	110	10	6	1000	15	0.88
Proteus	Dataset 2	Session 7	16.06849	0.59605	0.77688	0.00337	Te-1	1.004	110	10	6	1000	15	0.88
Proteus	Dataset 2	Session 7	11.49936	0.17990	0.75647	0.00131	Te-1	1.004	110	10	6	1000	15	0.88
Proteus	Dataset 2	Session 7	9.35102	0.18930	0.74763	0.00126	Te-1	1.004	110	10	6	1000	15	0.88
Proteus	Dataset 2	Session 8	8.66115	1.33267	0.74545	0.00652	NIST SRM610	1.004	110	10	6	1000	15	0.88
Proteus	Dataset 2	Session 8	0.20592	0.04886	0.71069	0.00083	NIST SRM610	1.004	110	10	6	1000	15	0.88
Proteus	Dataset 2	Session 8	27.12824	0.47750	0.81958	0.00382	NIST SRM610	1.004	110	10	6	1000	15	0.88
Proteus	Dataset 2	Session 8	15.24788	0.21759	0.77306	0.00262	NIST SRM610	1.004	110	10	6	1000	15	0.88
Proteus	Dataset 2	Session 8	24.17151	0.81752	0.80281	0.00421	NIST SRM610	1.004	110	10	6	1000	15	0.88
Proteus	Dataset 2	Session 8	27.85604	0.42842	0.82025	0.00348	NIST SRM610	1.004	110	10	6	1000	15	0.88
Proteus	Dataset 2	Session 8	17.06756	0.26784	0.77949	0.00259	NIST SRM610	1.004	110	10	6	1000	15	0.88
Proteus	Dataset 2	Session 8	20.92787	0.59091	0.79382	0.00381	NIST SRM610	1.004	110	10	6	1000	15	0.88
Proteus	Dataset 2	Session 8	24.63269	0.44181	0.80657	0.00387	NIST SRM610	1.004	110	10	6	1000	15	0.88
Proteus	Dataset 2	Session 8	0.07796	0.01993	0.70960	0.00080	NIST SRM610	1.004	110	10	6	1000	15	0.88
Proteus	Dataset 2	Session 8	25.71017	0.66018	0.80839	0.00371	NIST SRM610	1.004	110	10	6	1000	15	0.88
Proteus	Dataset 2	Session 8	16.22841	0.17245	0.77496	0.00179	NIST SRM610	1.004	110	10	6	1000	15	0.88
Proteus	Dataset 2	Session 8	19.12727	0.59205	0.78377	0.00371	NIST SRM610	1.004	110	10	6	1000	15	0.88
Proteus	Dataset 2	Session 8	23.92067	0.22588	0.80578	0.00234	NIST SRM610	1.004	110	10	6	1000	15	0.88
Proteus	Dataset 2	Session 8	0.07159	0.00386	0.71031	0.00074	NIST SRM610	1.004	110	10	6	1000	15	0.88
Proteus	Dataset 2	Session 8	19.54449	0.23741	0.78749	0.00208	NIST SRM610	1.004	110	10	6	1000	15	0.88
Proteus	Dataset 2	Session 8	0.06060	0.01071	0.70967	0.00065	NIST SRM610	1.004	110	10	6	1000	15	0.88
Proteus	Dataset 2	Session 8	0.10084	0.01709	0.71060	0.00187	NIST SRM610	1.004	110	10	6	1000	15	0.88
Proteus	Dataset 2	Session 8	20.67955	0.37321	0.79271	0.00185	NIST SRM610	1.004	110	10	6	1000	15	0.88
Proteus	Dataset 2	Session 8	15.84504	0.46251	0.76969	0.00216	NIST SRM610	1.004	110	10	6	1000	15	0.88
Proteus	Dataset 2	Session 8	20.53455	1.50598	0.78511	0.00620	NIST SRM610	1.004	110	10	6	1000	15	0.88
Proteus	Dataset 2	Session 8	0.78611	1.51794	0.70992	0.00055	NIST SRM610	1.004	110	10	6	1000	15	0.88
Proteus	Dataset 2	Session 8	15.17125	0.19933	0.77019	0.00140	NIST SRM610	1.004	110	10	6	1000	15	0.88
Proteus	Dataset 2	Session 8	0.31081	0.06608	0.71040	0.00069	NIST SRM610	1.004	110	10	6	1000	15	0.88
Proteus	Dataset 2	Session 8	0.06703	0.01438	0.70867	0.00135	NIST SRM610	1.004	110	10	6	1000	15	0.88
Proteus	Dataset 2	Session 8	20.05051	0.42454	0.78884	0.00346	NIST SRM610	1.004	110	10	6	1000	15	0.88
Proteus	Dataset 2	Session 8	14.26995	1.08660	0.76576	0.00478	NIST SRM610	1.004	110	10	6	1000	15	0.88

Proteus	Dataset 2	Session 8	11.97545	0.17595	0.75720	0.00134	NIST SRM610	1.004	110	10	6	1000	15	0.88
Proteus	Dataset 2	Session 8	16.43368	1.07751	0.77211	0.00429	NIST SRM610	1.004	110	10	6	1000	15	0.88
Proteus	Dataset 2	Session 8	0.05119	0.00596	0.70993	0.00101	NIST SRM610	1.004	110	10	6	1000	15	0.88
Proteus	Dataset 2	Session 8	16.75528	0.21630	0.77726	0.00190	NIST SRM610	1.004	110	10	6	1000	15	0.88
Proteus	Dataset 2	Session 8	21.66142	0.23480	0.79354	0.00259	NIST SRM610	1.004	110	10	6	1000	15	0.88
Proteus	Dataset 2	Session 8	9.67616	0.75710	0.74613	0.00389	NIST SRM610	1.004	110	10	6	1000	15	0.88
Proteus	Dataset 2	Session 8	14.78409	0.17795	0.76733	0.00200	NIST SRM610	1.004	110	10	6	1000	15	0.88
Proteus	Dataset 2	Session 8	0.05312	0.00931	0.70935	0.00066	NIST SRM610	1.004	110	10	6	1000	15	0.88
Proteus	Dataset 2	Session 8	0.02877	0.00307	0.70924	0.00078	NIST SRM610	1.004	110	10	6	1000	15	0.88
Proteus	Dataset 2	Session 8	19.09594	0.28460	0.78523	0.00229	NIST SRM610	1.004	110	10	6	1000	15	0.88
Proteus	Dataset 2	Session 8	15.25174	0.76347	0.76891	0.00315	NIST SRM610	1.004	110	10	6	1000	15	0.88
Proteus	Dataset 2	Session 8	20.47105	0.19599	0.78854	0.00272	NIST SRM610	1.004	110	10	6	1000	15	0.88
Proteus	Dataset 2	Session 8	16.75777	0.13353	0.77956	0.00180	NIST SRM610	1.004	110	10	6	1000	15	0.88
Proteus	Dataset 2	Session 8	18.43309	0.31085	0.78366	0.00214	NIST SRM610	1.004	110	10	6	1000	15	0.88
Proteus	Dataset 2	Session 8	0.06704	0.01639	0.71079	0.00087	NIST SRM610	1.004	110	10	6	1000	15	0.88
Proteus	Dataset 2	Session 8	0.13846	0.02894	0.71073	0.00082	NIST SRM610	1.004	110	10	6	1000	15	0.88
Proteus	Dataset 2	Session 8	17.52525	0.36066	0.78107	0.00239	NIST SRM610	1.004	110	10	6	1000	15	0.88
Proteus	Dataset 2	Session 8	0.26851	0.04340	0.71126	0.00138	NIST SRM610	1.004	110	10	6	1000	15	0.88
Proteus	Dataset 2	Session 8	19.82169	0.18725	0.78704	0.00237	NIST SRM610	1.004	110	10	6	1000	15	0.88
Proteus	Dataset 2	Session 8	14.25626	0.15013	0.76782	0.00156	NIST SRM610	1.004	110	10	6	1000	15	0.88
Proteus	Dataset 2	Session 8	17.27976	0.12836	0.77684	0.00229	NIST SRM610	1.004	110	10	6	1000	15	0.88
Proteus	Dataset 2	Session 8	0.03468	0.00314	0.70963	0.00070	NIST SRM610	1.004	110	10	6	1000	15	0.88
Proteus	Dataset 2	Session 8	15.44654	0.18568	0.77344	0.00176	NIST SRM610	1.004	110	10	6	1000	15	0.88
Proteus	Dataset 2	Session 8	18.86209	0.24156	0.78304	0.00205	NIST SRM610	1.004	110	10	6	1000	15	0.88
Proteus	Dataset 2	Session 8	0.17621	0.06779	0.71064	0.00109	NIST SRM610	1.004	110	10	6	1000	15	0.88
Proteus	Dataset 2	Session 8	18.70392	0.41286	0.78349	0.00234	NIST SRM610	1.004	110	10	6	1000	15	0.88
Proteus	Dataset 2	Session 9	0.03256	0.00393	0.70953	0.00076	NIST SRM610	1.004	110	10	6	1000	15	0.88
Proteus	Dataset 2	Session 9	24.32619	0.49807	0.80217	0.00339	NIST SRM610	1.004	110	10	6	1000	15	0.88
Proteus	Dataset 2	Session 9	21.65554	0.61893	0.79309	0.00505	NIST SRM610	1.004	110	10	6	1000	15	0.88
Proteus	Dataset 2	Session 9	0.09481	0.05009	0.70974	0.00070	NIST SRM610	1.004	110	10	6	1000	15	0.88
Proteus	Dataset 2	Session 9	0.22456	0.04738	0.71035	0.00068	NIST SRM610	1.004	110	10	6	1000	15	0.88
Proteus	Dataset 2	Session 9	17.85015	0.44687	0.77971	0.00300	NIST SRM610	1.004	110	10	6	1000	15	0.88
Proteus	Dataset 2	Session 9	19.82926	0.88964	0.78817	0.00366	NIST SRM610	1.004	110	10	6	1000	15	0.88
Proteus	Dataset 2	Session 9	10.18145	0.45671	0.75126	0.00247	NIST SRM610	1.004	110	10	6	1000	15	0.88
Proteus	Dataset 2	Session 9	10.66460	0.20632	0.75130	0.00161	NIST SRM610	1.004	110	10	6	1000	15	0.88
Proteus	Dataset 2	Session 9	12.15568	0.31305	0.75892	0.00147	NIST SRM610	1.004	110	10	6	1000	15	0.88
Proteus	Dataset 2	Session 9	20.18169	0.34186	0.79106	0.00276	NIST SRM610	1.004	110	10	6	1000	15	0.88
Proteus	Dataset 2	Session 9	0.03016	0.00134	0.70952	0.00064	NIST SRM610	1.004	110	10	6	1000	15	0.88
Proteus	Dataset 2	Session 9	33.36005	0.76812	0.83533	0.00474	NIST SRM610	1.004	110	10	6	1000	15	0.88
Proteus	Dataset 2	Session 9	25.81753	0.44864	0.81245	0.00338	NIST SRM610	1.004	110	10	6	1000	15	0.88
Proteus	Dataset 2	Session 9	33.76158	0.84621	0.84244	0.00642	NIST SRM610	1.004	110	10	6	1000	15	0.88
Proteus	Dataset 2	Session 9	31.53109	0.85359	0.83738	0.00458	NIST SRM610	1.004	110	10	6	1000	15	0.88
Proteus	Dataset 2	Session 9	21.84393	0.33711	0.79925	0.00302	NIST SRM610	1.004	110	10	6	1000	15	0.88
Proteus	Dataset 2	Session 9	14.29416	0.19776	0.76670	0.00173	NIST SRM610	1.004	110	10	6	1000	15	0.88
Proteus	Dataset 2	Session 9	16.33097	0.44489	0.77356	0.00218	NIST SRM610	1.004	110	10	6	1000	15	0.88
Proteus	Dataset 2	Session 9	14.07439	0.34036	0.76518	0.00217	NIST SRM610	1.004	110	10	6	1000	15	0.88
Proteus	Dataset 2	Session 9	23.44960	0.72495	0.80227	0.00412	NIST SRM610	1.004	110	10	6	1000	15	0.88
Proteus	Dataset 2	Session 9	13.74829	0.44366	0.76462	0.00259	NIST SRM610	1.004	110	10	6	1000	15	0.88
Proteus	Dataset 2	Session 9	10.07878	0.42134	0.75059	0.00167	NIST SRM610	1.004	110	10	6	1000	15	0.88
Proteus	Dataset 2	Session 9	13.97324	0.32204	0.76485	0.00213	NIST SRM610	1.004	110	10	6	1000	15	0.88
Proteus	Dataset 2	Session 9	14.89640	0.10932	0.76997	0.00182	NIST SRM610	1.004	110	10	6	1000	15	0.88
Proteus	Dataset 2	Session 9	24.74709	0.78941	0.81219	0.00367	NIST SRM610	1.004	110	10	6	1000	15	0.88
Proteus	Dataset 2	Session 9	30.78121	1.11113	0.83015	0.00399	NIST SRM610	1.004	110	10	6	1000	15	0.88
Proteus	Dataset 2	Session 9	0.02635	0.00115	0.70886	0.00084	NIST SRM610	1.004	110	10	6	1000	15	0.88
Proteus	Dataset 2	Session 9	6.36276	0.09659	0.73570	0.00098	NIST SRM610	1.004	110	10	6	1000	15	0.88
Proteus	Dataset 2	Session 9	8.43038	0.12281	0.74230	0.00115	NIST SRM610	1.004	110	10	6	1000	15	0.88
Proteus	Dataset 2	Session 9	9.24710	0.26347	0.74464	0.00143	NIST SRM610	1.004	110	10	6	1000	15	0.88
Proteus	Dataset 2	Session 9	33.18737	0.64164	0.83777	0.00509	NIST SRM610	1.004	110	10	6	1000	15	0.88
Proteus	Dataset 2	Session 9	15.44836	0.40515	0.75513	0.02990	NIST SRM610	1.004	110	10	6	1000	15	0.88
Proteus	Dataset 2	Session 9	0.02595	0.00210	0.70854	0.00105	NIST SRM610	1.004	110	10	6	1000	15	0.88
Proteus	Dataset 2	Session 9	0.02891	0.00356	0.70988	0.00090	NIST SRM610	1.004	110	10	6	1000	15	0.88
Proteus	Dataset 2	Session 9	0.02609	0.00157	0.70879	0.00076	NIST SRM610	1.004	110	10	6	1000	15	0.88
Proteus	Dataset 2	Session 9	26.56715	0.48811	0.81837	0.00368	NIST SRM610	1.004	110	10	6	1000	15	0.88
Proteus	Dataset 2	Session 9	24.96468	0.29943	0.80904	0.00405	NIST SRM610	1.004	110	10	6	1000	15	0.88
Proteus	Dataset 2	Session 9	24.07989	0.36763	0.80231	0.00405	NIST SRM610	1.004	110	10	6	1000	15	0.88
Proteus	Dataset 2	Session 9	0.06827	0.01000	0.70995	0.00085	NIST SRM610	1.004	110	10	6	1000	15	0.88
Proteus	Dataset 2	Session 9	26.46459	0.80080	0.81558	0.00394	NIST SRM610	1.004	110	10	6	1000	15	0.88
Proteus	Dataset 2	Session 9	25.05711	0.60045	0.80708	0.00351	NIST SRM610	1.004	110	10	6	1000	15	0.88
Proteus	Dataset 2	Session 9	22.82202	0.50254	0.80067	0.00330	NIST SRM610	1.004	110	10	6	1000	15	0.88
Proteus	Dataset 2	Session 9	22.84913	0.43783	0.80300	0.00285	NIST SRM610	1.004	110	10	6	1000	15	0.88
Proteus	Dataset 2	Session 9	22.76904	0.59881	0.79600	0.00346	NIST SRM610	1.004	110	10	6	1000	15	0.88
Proteus	Dataset 2	Session 9	8.82093	1.68100	0.74052	0.00138	NIST SRM610	1.004	110	10	6	1000	15	0.88
Proteus	Dataset 2	Session 9	10.00961	0.24720	0.74930	0.00164	NIST SRM610	1.004	110	10	6	1000	15	0.88
Proteus	Dataset 2	Session 9	8.98030	0.07214	0.74490	0.00138	NIST SRM610	1.004	110	10	6	1000	15	0.88
Proteus	Dataset 2	Session 9	9.05521	0.21355	0.74479	0.00117	NIST SRM610	1.004	110	10	6	1000	15	0.88
Proteus	Dataset 2	Session 9	7.88857	0.10497	0.74110	0.00136	NIST SRM610	1.004	110	10	6	1000	15	0.88
Proteus	Dataset 2	Session 9	36.71359	0.67307	0.84878	0.00437	NIST SRM610	1.004	110	10	6	1000	15	0.88
Proteus	Dataset 2	Session 9	0.03670	0.00185	0.70968	0.00066	NIST SRM610	1.004	110	10	6	1000	15	0.88
Proteus	Dataset 2	Session 9	23.64461	0.84208	0.80327	0.00315	NIST SRM610	1.004	110	10	6	1000	15	0.88
Proteus	Dataset 2	Session 9	22.06494	0.59710	0.79704	0.00300	NIST SRM610	1.004	110	10	6	1000	15	0.88
Proteus	Dataset 2	Session 9	26.11891	0.57146	0.81322	0.00404	NIST SRM610	1.004	110	10	6	1000	15	0.88
Proteus	Dataset 2	Session 9	22.99356	0.85668	0.80054	0.00277	NIST SRM610	1.004	110	10	6	1000	15	0.88
Proteus	Dataset 2	Session 9	8.01673	0.15766	0.74240	0.00119	NIST SRM610	1.004	110	10	6	1000	15	0.88
Proteus	Dataset 2	Session 9	10.00189	0.16708	0.74975	0.00141	NIST SRM610	1.004	110	10	6	1000	15	0.88
Proteus	Dataset 2	Session 9	10.50889	0.14250	0.75096	0.00131	NIST SRM610	1.004	110	10	6	1000	15	0.88
Proteus	Dataset 2	Session 9	7.90945	0.12109	0.74057	0.00150	NIST SRM610	1.004	110	10	6	1000	15	0.88
Proteus	Dataset 2	Session 9	26.30313	0.46516	0.81296	0.00387	NIST SRM610	1.004	110	10	6	1000	15	0.88
Proteus	Dataset 2	Session 9	26.23949	0.61215	0.81686	0.00399	NIST SRM610	1.004	110	10	6	1000	15	0.88
Proteus	Dataset 2	Session 9	0.02392	0.00071	0.70987	0.00071	NIST SRM610	1.004	110	10	6	1000	15	0.88
Proteus	Dataset 2	Session 9	24.43009	0.57722	0.80701	0.00338	NIST SRM610	1.004	110	10	6	1000	15	0.88
Proteus	Dataset 2	Session 9	25.56176	0.51312	0.80987	0.00434	NIST SRM610	1.004	110	10	6	1000	15	0.88
Proteus	Dataset 2	Session 9	8.34134	0.19982	0.74187	0.00146	NIST SRM610	1.004	110	10	6	1000	15	0.88
Proteus	Dataset 2	Session 9	11.10635	0.19832	0.75491	0.00161	NIST SRM610	1.004	110	10	6	1000	15	0.88
Proteus	Dataset 2	Session 9	9.45868	0.10100	0.74815	0.00134	NIST SRM610	1.004	110	10	6	1000	15	0.88
Proteus	Dataset 2	Session 9	7.65516	0.24413	0.74106	0.00152	NIST SRM610	1.004	110	10	6	1000	15	0.88

Table S4: Data plotted in Figure 7

Sample: FCT-1

Instrument	Datset	Analysis Session	$^{87}Rb/^{86}Sr$	2SE	$^{87}Sr/^{86}Sr$	2SE	$^{87}Sr/^{86}Sr$ standard	$^{87}Rb/^{86}Sr$ correction factor	Spot diameter µm	Repetiton rate (Hz)	Fluence (J/cm^2)	Pulse count	Torch Depth (mm)	Sample gas (l/min)
Proteus	Dataset 1	Session 3	0.00169	0.00093	0.70583	0.00007	NIST SRM610	0.975	110	10	6	1000	15	0.96
Proteus	Dataset 1	Session 3	0.00146	0.00101	0.70602	0.00009	NIST SRM610	0.975	110	10	6	1000	15	0.96
Proteus	Dataset 1	Session 3	0.00110	0.00009	0.70593	0.00007	NIST SRM610	0.975	110	10	6	1000	15	0.96
Proteus	Dataset 1	Session 3	12.41676	3.78798	0.71158	0.00180	NIST SRM610	0.975	110	10	6	1000	15	0.96
Proteus	Dataset 1	Session 3	15.31544	3.71296	0.71455	0.00219	NIST SRM610	0.975	110	10	6	1000	15	0.96
Proteus	Dataset 1	Session 3	26.71917	4.33009	0.71630	0.00245	NIST SRM610	0.975	110	10	6	1000	15	0.96
Proteus	Dataset 1	Session 3	31.69396	4.37127	0.71604	0.00263	NIST SRM610	0.975	110	10	6	1000	15	0.96
Proteus	Dataset 1	Session 3	31.98310	1.98505	0.71564	0.00221	NIST SRM610	0.975	110	10	6	1000	15	0.96
Proteus	Dataset 1	Session 3	19.19255	5.23044	0.71196	0.00184	NIST SRM610	0.975	110	10	6	1000	15	0.96
Proteus	Dataset 1	Session 5	32.23613	2.43950	0.71750	0.00313	NIST SRM610	0.975	110	10	6	1000	15	0.96
Proteus	Dataset 1	Session 5	27.55851	3.19542	0.71699	0.00345	NIST SRM610	0.975	110	10	6	1000	15	0.96
Proteus	Dataset 1	Session 5	28.18843	2.11675	0.71369	0.00244	NIST SRM610	0.975	110	10	6	1000	15	0.96
Proteus	Dataset 1	Session 5	16.56506	1.72384	0.71408	0.00284	NIST SRM610	0.975	110	10	6	1000	15	0.96
Proteus	Dataset 1	Session 5	21.47972	1.46640	0.71638	0.00375	NIST SRM610	0.975	110	10	6	1000	15	0.96
Proteus	Dataset 1	Session 5	17.10836	2.06845	0.71328	0.00310	NIST SRM610	0.975	110	10	6	1000	15	0.96
Proteus	Dataset 1	Session 5	23.07168	0.96490	0.71748	0.00371	NIST SRM610	0.975	110	10	6	1000	15	0.96
Proteus	Dataset 1	Session 5	30.87814	2.53454	0.71869	0.00231	NIST SRM610	0.975	110	10	6	1000	15	0.96
Proteus	Dataset 1	Session 5	14.16147	1.70950	0.71374	0.00304	NIST SRM610	0.975	110	10	6	1000	15	0.96
Proteus	Dataset 1	Session 5	15.01951	1.10154	0.71319	0.00303	NIST SRM610	0.975	110	10	6	1000	15	0.96
Proteus	Dataset 1	Session 5	18.68698	0.96446	0.71621	0.00358	NIST SRM610	0.975	110	10	6	1000	15	0.96
Proteus	Dataset 1	Session 5	20.20960	2.53312	0.71573	0.00270	NIST SRM610	0.975	110	10	6	1000	15	0.96
Proteus	Dataset 1	Session 5	22.84588	4.09950	0.71479	0.00277	NIST SRM610	0.975	110	10	6	1000	15	0.96
Proteus	Dataset 1	Session 5	25.91960	1.61908	0.71621	0.00197	NIST SRM610	0.975	110	10	6	1000	15	0.96

Proteus	Dataset 1	Session 5	16.32591	2.02096	0.71247	0.00284	NIST SRM610	0.975	110	10	6	1000	15	0.96
Proteus	Dataset 1	Session 5	19.44340	4.49018	0.71492	0.00268	NIST SRM610	0.975	110	10	6	1000	15	0.96
Proteus	Dataset 1	Session 5	21.50326	1.77026	0.71495	0.00349	NIST SRM610	0.975	110	10	6	1000	15	0.96
Proteus	Dataset 1	Session 5	29.50398	1.98087	0.71818	0.00406	NIST SRM610	0.975	110	10	6	1000	15	0.96
Proteus	Dataset 1	Session 5	38.51357	8.02509	0.71590	0.00316	NIST SRM610	0.975	110	10	6	1000	15	0.96
Proteus	Dataset 1	Session 5	19.53996	1.43813	0.71473	0.00356	NIST SRM610	0.975	110	10	6	1000	15	0.96
Proteus	Dataset 1	Session 5	11.71703	3.93209	0.71060	0.00364	NIST SRM610	0.975	110	10	6	1000	15	0.96
Proteus	Dataset 1	Session 5	34.06603	6.45279	0.71886	0.00396	NIST SRM610	0.975	110	10	6	1000	15	0.96
Proteus	Dataset 1	Session 5	35.95692	1.95443	0.72031	0.00447	NIST SRM610	0.975	110	10	6	1000	15	0.96
Proteus	Dataset 1	Session 5	21.34836	2.06099	0.71408	0.00143	NIST SRM610	0.975	110	10	6	1000	15	0.96
Proteus	Dataset 1	Session 5	20.57103	4.42397	0.71473	0.00208	NIST SRM610	0.975	110	10	6	1000	15	0.96
Proteus	Dataset 2	Session 8	16.26935	1.30745	0.71288	0.00390	NIST SRM610	1.004	110	10	6	1000	15	0.88
Proteus	Dataset 2	Session 8	20.55539	0.47724	0.71684	0.00455	NIST SRM610	1.004	110	10	6	1000	15	0.88
Proteus	Dataset 2	Session 8	8.70702	0.44140	0.71122	0.00174	NIST SRM610	1.004	110	10	6	1000	15	0.88
Proteus	Dataset 2	Session 8	46.60302	2.37411	0.72567	0.00458	NIST SRM610	1.004	110	10	6	1000	15	0.88
Proteus	Dataset 2	Session 8	16.24428	5.05001	0.71166	0.00269	NIST SRM610	1.004	110	10	6	1000	15	0.88
Proteus	Dataset 2	Session 8	11.04462	1.49118	0.70845	0.00310	NIST SRM610	1.004	110	10	6	1000	15	0.88
Proteus	Dataset 2	Session 8	27.10883	1.31502	0.71671	0.00302	NIST SRM610	1.004	110	10	6	1000	15	0.88
Proteus	Dataset 2	Session 8	2.40925	0.26608	0.70705	0.00145	NIST SRM610	1.004	110	10	6	1000	15	0.88
Proteus	Dataset 2	Session 8	23.71469	2.79076	0.71646	0.00438	NIST SRM610	1.004	110	10	6	1000	15	0.88
Proteus	Dataset 2	Session 8	8.21743	1.31180	0.70902	0.00167	NIST SRM610	1.004	110	10	6	1000	15	0.88
Proteus	Dataset 2	Session 8	14.72453	0.81724	0.71445	0.00594	NIST SRM610	1.004	110	10	6	1000	15	0.88
Proteus	Dataset 2	Session 8	22.14007	1.26992	0.71391	0.00528	NIST SRM610	1.004	110	10	6	1000	15	0.88
Proteus	Dataset 2	Session 8	0.00214	0.00017	0.70602	0.00012	NIST SRM610	1.004	110	10	6	1000	15	0.88
Proteus	Dataset 2	Session 8	0.00531	0.00106	0.70590	0.00016	NIST SRM610	1.004	110	10	6	1000	15	0.88
Proteus	Dataset 2	Session 8	0.00581	0.00094	0.70615	0.00014	NIST SRM610	1.004	110	10	6	1000	15	0.88
Proteus	Dataset 2	Session 9	0.00557	0.00817	0.70593	0.00014	NIST SRM610	1.004	110	10	6	1000	15	0.88
Proteus	Dataset 2	Session 9	0.00183	0.00022	0.70584	0.00018	NIST SRM610	1.004	110	10	6	1000	15	0.88
Proteus	Dataset 2	Session 9	0.00247	0.00043	0.70586	0.00026	NIST SRM610	1.004	110	10	6	1000	15	0.88
Proteus	Dataset 2	Session 9	7.93930	0.60471	0.70830	0.00204	NIST SRM610	1.004	110	10	6	1000	15	0.88
Proteus	Dataset 2	Session 9	8.96427	1.81720	0.71147	0.00355	NIST SRM610	1.004	110	10	6	1000	15	0.88
Proteus	Dataset 2	Session 9	19.81565	4.20669	0.71010	0.00188	NIST SRM610	1.004	110	10	6	1000	15	0.88
Proteus	Dataset 2	Session 9	13.36569	1.09600	0.71234	0.00423	NIST SRM610	1.004	110	10	6	1000	15	0.88
Proteus	Dataset 2	Session 9	25.95962	0.99601	0.71919	0.00367	NIST SRM610	1.004	110	10	6	1000	15	0.88
Proteus	Dataset 2	Session 9	25.75013	0.81488	0.71608	0.00411	NIST SRM610	1.004	110	10	6	1000	15	0.88
Proteus	Dataset 2	Session 9	44.28187	5.54018	0.72025	0.00344	NIST SRM610	1.004	110	10	6	1000	15	0.88
Proteus	Dataset 2	Session 9	59.52842	3.60668	0.72571	0.00318	NIST SRM610	1.004	110	10	6	1000	15	0.88
Proteus	Dataset 2	Session 9	11.61055	1.71203	0.71409	0.00310	NIST SRM610	1.004	110	10	6	1000	15	0.88
Proteus	Dataset 2	Session 9	13.87937	1.05353	0.71500	0.00318	NIST SRM610	1.004	110	10	6	1000	15	0.88
Proteus	Dataset 2	Session 9	5.93777	0.32606	0.70784	0.00103	NIST SRM610	1.004	110	10	6	1000	15	0.88
Proteus	Dataset 2	Session 9	25.99595	1.04803	0.71684	0.00625	NIST SRM610	1.004	110	10	6	1000	15	0.88
Proteus	Dataset 2	Session 9	10.16950	1.33978	0.70902	0.00298	NIST SRM610	1.004	110	10	6	1000	15	0.88
Proteus	Dataset 2	Session 9	17.05506	1.91312	0.71172	0.00322	NIST SRM610	1.004	110	10	6	1000	15	0.88
Proteus	Dataset 2	Session 9	5.40702	1.08759	0.70720	0.00089	NIST SRM610	1.004	110	10	6	1000	15	0.88
Proteus	Dataset 2	Session 9	14.59579	2.57847	0.70907	0.00305	NIST SRM610	1.004	110	10	6	1000	15	0.88
Proteus	Dataset 2	Session 9	10.49606	1.11673	0.71014	0.00271	NIST SRM610	1.004	110	10	6	1000	15	0.88
Proteus	Dataset 2	Session 9	17.00827	1.26158	0.71535	0.00379	NIST SRM610	1.004	110	10	6	1000	15	0.88
Proteus	Dataset 2	Session 9	22.49656	0.53456	0.71620	0.00331	NIST SRM610	1.004	110	10	6	1000	15	0.88
Proteus	Dataset 2	Session 9	17.28909	0.98571	0.71320	0.00367	NIST SRM610	1.004	110	10	6	1000	15	0.88
Proteus	Dataset 2	Session 9	15.06705	0.72597	0.71240	0.00493	NIST SRM610	1.004	110	10	6	1000	15	0.88

Table S5: Data plotted in Figure 8

Sample: SG-1

Instrument	Dataset	Analysis Session	$^{87}Rb/^{86}Sr$	2SE	$^{87}Sr/^{86}Sr$	2SE	$^{87}Sr/^{86}Sr$ standard	$^{87}Rb/^{86}Sr$ correction factor	Spot diameter μm	Repetiton rate (Hz)	Fluence (J/cm^2)	Pulse count	Torch Depth (mm)	Sample gas (l/min)
Proteus	Dataset 1	Session 1	1.28547	0.01403	0.71447	0.00032	Te-1	0.975	110	10	6	1000	15	0.96
Proteus	Dataset 1	Session 1	1.06390	0.00770	0.71333	0.00028	Te-1	0.975	110	10	6	1000	15	0.96
Proteus	Dataset 1	Session 1	1.12358	0.00475	0.71355	0.00027	Te-1	0.975	110	10	6	1000	15	0.96
Proteus	Dataset 1	Session 1	0.08315	0.00629	0.70800	0.00031	Te-1	0.975	110	10	6	1000	15	0.96
Proteus	Dataset 1	Session 1	0.19761	0.01250	0.70921	0.00037	Te-1	0.975	110	10	6	1000	15	0.96
Proteus	Dataset 1	Session 1	1.19438	0.22839	0.71340	0.00028	Te-1	0.975	110	10	6	1000	15	0.96
Proteus	Dataset 1	Session 1	0.24085	0.01662	0.70912	0.00030	Te-1	0.975	110	10	6	1000	15	0.96
Proteus	Dataset 1	Session 1	0.97318	0.00824	0.71255	0.00028	Te-1	0.975	110	10	6	1000	15	0.96
Proteus	Dataset 1	Session 1	0.26147	0.01430	0.70864	0.00029	Te-1	0.975	110	10	6	1000	15	0.96
Proteus	Dataset 1	Session 1	2.73431	0.07700	0.72257	0.00053	Te-1	0.975	110	10	6	1000	15	0.96
Proteus	Dataset 1	Session 1	1.18084	0.00773	0.71426	0.00026	Te-1	0.975	110	10	6	1000	15	0.96
Proteus	Dataset 1	Session 1	0.06520	0.00219	0.70759	0.00024	Te-1	0.975	110	10	6	1000	15	0.96
Proteus	Dataset 1	Session 1	0.01737	0.00240	0.70749	0.00027	Te-1	0.975	110	10	6	1000	15	0.96
Proteus	Dataset 1	Session 1	0.03513	0.00226	0.70770	0.00026	Te-1	0.975	110	10	6	1000	15	0.96
Proteus	Dataset 1	Session 1	1.00827	0.08713	0.71365	0.00057	Te-1	0.975	110	10	6	1000	15	0.96
Proteus	Dataset 1	Session 1	1.08423	0.00564	0.71370	0.00031	Te-1	0.975	110	10	6	1000	15	0.96
Proteus	Dataset 1	Session 1	4.71338	0.11666	0.73394	0.00113	Te-1	0.975	110	10	6	1000	15	0.96
Proteus	Dataset 1	Session 1	0.35086	0.01484	0.70973	0.00026	Te-1	0.975	110	10	6	1000	15	0.96
Proteus	Dataset 1	Session 1	1.00176	0.00826	0.71299	0.00026	Te-1	0.975	110	10	6	1000	15	0.96
Proteus	Dataset 1	Session 1	0.23714	0.00920	0.70916	0.00018	Te-1	0.975	110	10	6	1000	15	0.96
Proteus	Dataset 1	Session 1	0.98651	0.11692	0.71293	0.00080	Te-1	0.975	110	10	6	1000	15	0.96
Proteus	Dataset 1	Session 1	0.15483	0.00721	0.70840	0.00029	Te-1	0.975	110	10	6	1000	15	0.96
Proteus	Dataset 1	Session 1	1.37638	0.04458	0.71562	0.00028	Te-1	0.975	110	10	6	1000	15	0.96
Proteus	Dataset 1	Session 1	1.11667	0.00754	0.71383	0.00029	Te-1	0.975	110	10	6	1000	15	0.96
Proteus	Dataset 1	Session 1	1.18905	0.04034	0.71417	0.00035	Te-1	0.975	110	10	6	1000	15	0.96
Proteus	Dataset 1	Session 1	0.13774	0.00678	0.70857	0.00021	Te-1	0.975	110	10	6	1000	15	0.96
Proteus	Dataset 1	Session 1	7.97920	0.26158	0.75247	0.00168	Te-1	0.975	110	10	6	1000	15	0.96
Proteus	Dataset 1	Session 3	20.92189	0.43014	0.82461	0.00203	NIST SRM610	0.975	110	10	6	1000	15	0.96
Proteus	Dataset 1	Session 3	23.22876	0.81770	0.83838	0.00478	NIST SRM610	0.975	110	10	6	1000	15	0.96
Proteus	Dataset 1	Session 3	8.97687	0.24742	0.75574	0.00167	NIST SRM610	0.975	110	10	6	1000	15	0.96
Proteus	Dataset 1	Session 3	1.10940	0.02172	0.71387	0.00025	NIST SRM610	0.975	110	10	6	1000	15	0.96
Proteus	Dataset 1	Session 3	21.42661	0.54666	0.82768	0.00333	NIST SRM610	0.975	110	10	6	1000	15	0.96
Proteus	Dataset 1	Session 3	1.18200	0.03949	0.71463	0.00022	NIST SRM610	0.975	110	10	6	1000	15	0.96
Proteus	Dataset 1	Session 3	2.34939	0.03471	0.72146	0.00039	NIST SRM610	0.975	110	10	6	1000	15	0.96
Proteus	Dataset 1	Session 3	1.43265	0.01428	0.71550	0.00024	NIST SRM610	0.975	110	10	6	1000	15	0.96
Proteus	Dataset 1	Session 3	1.46317	0.05985	0.71549	0.00032	NIST SRM610	0.975	110	10	6	1000	15	0.96
Proteus	Dataset 1	Session 3	1.22422	0.01070	0.71463	0.00022	NIST SRM610	0.975	110	10	6	1000	15	0.96
Proteus	Dataset 1	Session 3	20.75196	0.35641	0.82720	0.00254	NIST SRM610	0.975	110	10	6	1000	15	0.96
Proteus	Dataset 1	Session 3	0.98316	0.00543	0.71363	0.00026	NIST SRM610	0.975	110	10	6	1000	15	0.96
Proteus	Dataset 1	Session 3	0.64045	0.23546	0.71282	0.00181	NIST SRM610	0.975	110	10	6	1000	15	0.96
Proteus	Dataset 1	Session 3	0.35670	0.03414	0.71026	0.00028	NIST SRM610	0.975	110	10	6	1000	15	0.96
Proteus	Dataset 1	Session 3	2.84473	0.88068	0.72531	0.00521	NIST SRM610	0.975	110	10	6	1000	15	0.96
Proteus	Dataset 1	Session 3	0.90889	0.12844	0.71308	0.00098	NIST SRM610	0.975	110	10	6	1000	15	0.96
Proteus	Dataset 1	Session 3	1.09598	1.39116	0.71071	0.00050	NIST SRM610	0.975	110	10	6	1000	15	0.96
Proteus	Dataset 1	Session 3	1.97434	0.07765	0.71901	0.00046	NIST SRM610	0.975	110	10	6	1000	15	0.96
Proteus	Dataset 1	Session 3	2.16204	0.01855	0.72008	0.00026	NIST SRM610	0.975	110	10	6	1000	15	0.96
Proteus	Dataset 1	Session 3	1.29709	0.02323	0.71492	0.00021	NIST SRM610	0.975	110	10	6	1000	15	0.96
Proteus	Dataset 1	Session 4	1.26953	0.01203	0.71459	0.00020	Te-1	0.975	110	10	6	1000	15	0.96
Proteus	Dataset 1	Session 4	1.05534	0.01800	0.71337	0.00028	Te-1	0.975	110	10	6	1000	15	0.96
Proteus	Dataset 1	Session 4	22.57757	0.67932	0.83042	0.00350	Te-1	0.975	110	10	6	1000	15	0.96
Proteus	Dataset 1	Session 4	13.20830	1.09499	0.78903	0.02577	Te-1	0.975	110	10	6	1000	15	0.96
Proteus	Dataset 1	Session 4	17.49712	0.85398	0.79921	0.00471	Te-1	0.975	110	10	6	1000	15	0.96
Proteus	Dataset 1	Session 4	1.13087	0.00922	0.71382	0.00019	Te-1	0.975	110	10	6	1000	15	0.96
Proteus	Dataset 1	Session 4	0.90594	0.01723	0.71260	0.00017	Te-1	0.975	110	10	6	1000	15	0.96
Proteus	Dataset 1	Session 4	19.44324	0.44898	0.81298	0.00245	Te-1	0.975	110	10	6	1000	15	0.96
Proteus	Dataset 1	Session 4	15.60864	0.75526	0.79442	0.00322	Te-1	0.975	110	10	6	1000	15	0.96
Proteus	Dataset 1	Session 4	13.32807	0.57639	0.78297	0.00379	Te-1	0.975	110	10	6	1000	15	0.96
Proteus	Dataset 1	Session 4	0.97339	0.01031	0.71332	0.00028	Te-1	0.975	110	10	6	1000	15	0.96
Proteus	Dataset 1	Session 4	1.15213	0.02353	0.71378	0.00029	Te-1	0.975	110	10	6	1000	15	0.96
Proteus	Dataset 1	Session 4	0.03325	0.02449	0.70747	0.00018	Te-1	0.975	110	10	6	1000	15	0.96
Proteus	Dataset 1	Session 4	1.10175	0.00747	0.71325	0.00024	Te-1	0.975	110	10	6	1000	15	0.96
Proteus	Dataset 1	Session 4	0.02045	0.00970	0.70765	0.00023	Te-1	0.975	110	10	6	1000	15	0.96
Proteus	Dataset 1	Session 4	0.01136	0.00053	0.70770	0.00019	Te-1	0.975	110	10	6	1000	15	0.96
Proteus	Dataset 1	Session 4	1.10269	0.01138	0.71373	0.00028	Te-1	0.975	110	10	6	1000	15	0.96
Proteus	Dataset 1	Session 4	1.02863	0.00906	0.71324	0.00032	Te-1	0.975	110	10	6	1000	15	0.96
Proteus	Dataset 1	Session 4	1.34444	0.03912	0.71468	0.00029	Te-1	0.975	110	10	6	1000	15	0.96
Proteus	Dataset 1	Session 4	1.14142	0.01232	0.71397	0.00034	Te-1	0.975	110	10	6	1000	15	0.96

Proteus	Dataset 1	Session 4	0.98594	0.01878	0.71281	0.00029	Te-1	0.975	110	10	6	1000	15	0.96
Proteus	Dataset 1	Session 4	15.14449	0.68161	0.78892	0.00337	Te-1	0.975	110	10	6	1000	15	0.96
Proteus	Dataset 1	Session 4	1.14034	0.00633	0.71416	0.00025	Te-1	0.975	110	10	6	1000	15	0.96
Proteus	Dataset 1	Session 4	1.16620	0.00790	0.71377	0.00026	Te-1	0.975	110	10	6	1000	15	0.96
Proteus	Dataset 1	Session 4	1.16068	0.00843	0.71384	0.00031	Te-1	0.975	110	10	6	1000	15	0.96
Proteus	Dataset 1	Session 4	1.19048	0.01372	0.71418	0.00030	Te-1	0.975	110	10	6	1000	15	0.96
Proteus	Dataset 1	Session 4	1.23365	0.01548	0.71429	0.00033	Te-1	0.975	110	10	6	1000	15	0.96
Proteus	Dataset 1	Session 4	1.20787	0.00612	0.71440	0.00042	Te-1	0.975	110	10	6	1000	15	0.96
Proteus	Dataset 1	Session 4	1.96416	0.01119	0.71884	0.00044	Te-1	0.975	110	10	6	1000	15	0.96
Proteus	Dataset 1	Session 4	0.00529	0.00042	0.70769	0.00022	Te-1	0.975	110	10	6	1000	15	0.96
Proteus	Dataset 1	Session 4	0.00475	0.00028	0.70743	0.00024	Te-1	0.975	110	10	6	1000	15	0.96
Proteus	Dataset 1	Session 4	0.16102	0.03821	0.70824	0.00021	Te-1	0.975	110	10	6	1000	15	0.96
Proteus	Dataset 1	Session 4	1.47011	0.04028	0.71603	0.00042	Te-1	0.975	110	10	6	1000	15	0.96
Proteus	Dataset 1	Session 4	1.21087	0.04510	0.71439	0.00030	Te-1	0.975	110	10	6	1000	15	0.96
Proteus	Dataset 1	Session 4	0.95750	0.01584	0.71269	0.00024	Te-1	0.975	110	10	6	1000	15	0.96
Proteus	Dataset 1	Session 4	0.95974	0.01638	0.71291	0.00030	Te-1	0.975	110	10	6	1000	15	0.96
Proteus	Dataset 1	Session 4	0.04190	0.00802	0.70773	0.00023	Te-1	0.975	110	10	6	1000	15	0.96
Proteus	Dataset 1	Session 4	0.98005	0.00622	0.71334	0.00043	Te-1	0.975	110	10	6	1000	15	0.96
Proteus	Dataset 1	Session 4	0.99224	0.01056	0.71346	0.00032	Te-1	0.975	110	10	6	1000	15	0.96
Proteus	Dataset 1	Session 4	9.95712	0.31969	0.76469	0.00245	Te-1	0.975	110	10	6	1000	15	0.96
Proteus	Dataset 1	Session 4	10.05752	0.31852	0.76134	0.00172	Te-1	0.975	110	10	6	1000	15	0.96
Proteus	Dataset 1	Session 4	14.73654	0.44613	0.78841	0.00237	Te-1	0.975	110	10	6	1000	15	0.96
Proteus	Dataset 1	Session 4	18.65930	1.06902	0.81199	0.00282	Te-1	0.975	110	10	6	1000	15	0.96
Proteus	Dataset 1	Session 4	16.78946	0.44965	0.80164	0.00256	Te-1	0.975	110	10	6	1000	15	0.96
Proteus	Dataset 1	Session 4	16.67489	1.98511	0.80098	0.01123	Te-1	0.975	110	10	6	1000	15	0.96
Proteus	Dataset 1	Session 4	1.13728	0.03431	0.71417	0.00040	Te-1	0.975	110	10	6	1000	15	0.96
Proteus	Dataset 1	Session 4	1.24898	0.07110	0.71413	0.00045	Te-1	0.975	110	10	6	1000	15	0.96
Proteus	Dataset 1	Session 4	0.98630	0.00743	0.71269	0.00027	Te-1	0.975	110	10	6	1000	15	0.96
Proteus	Dataset 1	Session 4	1.05901	0.01550	0.71349	0.00026	Te-1	0.975	110	10	6	1000	15	0.96
Proteus	Dataset 1	Session 4	0.68066	0.01615	0.71126	0.00024	Te-1	0.975	110	10	6	1000	15	0.96
Proteus	Dataset 1	Session 4	0.91543	0.01333	0.71259	0.00024	Te-1	0.975	110	10	6	1000	15	0.96
Proteus	Dataset 1	Session 4	0.97318	0.00725	0.71311	0.00034	Te-1	0.975	110	10	6	1000	15	0.96
Proteus	Dataset 1	Session 4	1.03697	0.01485	0.71328	0.00033	Te-1	0.975	110	10	6	1000	15	0.96
Proteus	Dataset 1	Session 6	0.00424	0.00672	0.70793	0.00098	Te-1	0.975	110	10	6	1000	15	0.96
Proteus	Dataset 1	Session 6	1.19884	0.02130	0.71383	0.00020	Te-1	0.975	110	10	6	1000	15	0.96
Proteus	Dataset 1	Session 6	8.43669	0.13539	0.75393	0.00121	Te-1	0.975	110	10	6	1000	15	0.96
Proteus	Dataset 1	Session 6	10.86071	0.51474	0.76629	0.00107	Te-1	0.975	110	10	6	1000	15	0.96
Proteus	Dataset 1	Session 6	9.57641	0.64479	0.75764	0.00254	Te-1	0.975	110	10	6	1000	15	0.96
Proteus	Dataset 1	Session 6	0.68462	0.07650	0.71173	0.00059	Te-1	0.975	110	10	6	1000	15	0.96
Proteus	Dataset 1	Session 6	1.00310	0.07472	0.71345	0.00050	Te-1	0.975	110	10	6	1000	15	0.96
Proteus	Dataset 1	Session 6	8.23441	0.07474	0.75383	0.00125	Te-1	0.975	110	10	6	1000	15	0.96
Proteus	Dataset 1	Session 6	6.87374	0.12722	0.74544	0.00109	Te-1	0.975	110	10	6	1000	15	0.96
Proteus	Dataset 1	Session 6	0.07038	0.01383	0.70785	0.00023	Te-1	0.975	110	10	6	1000	15	0.96
Proteus	Dataset 1	Session 6	1.64408	0.05432	0.71619	0.00048	Te-1	0.975	110	10	6	1000	15	0.96
Proteus	Dataset 1	Session 6	1.54760	0.02730	0.71570	0.00045	Te-1	0.975	110	10	6	1000	15	0.96
Proteus	Dataset 1	Session 6	3.91588	0.65926	0.72840	0.00358	Te-1	0.975	110	10	6	1000	15	0.96
Proteus	Dataset 1	Session 6	7.35305	0.89360	0.74540	0.00496	Te-1	0.975	110	10	6	1000	15	0.96
Proteus	Dataset 1	Session 6	3.72164	0.88030	0.72786	0.00456	Te-1	0.975	110	10	6	1000	15	0.96
Proteus	Dataset 1	Session 6	0.02132	0.00947	0.70768	0.00022	Te-1	0.975	110	10	6	1000	15	0.96
Proteus	Dataset 1	Session 6	0.78771	0.05766	0.71200	0.00033	Te-1	0.975	110	10	6	1000	15	0.96
Proteus	Dataset 1	Session 6	9.57131	0.23285	0.76093	0.00127	Te-1	0.975	110	10	6	1000	15	0.96
Proteus	Dataset 1	Session 6	9.98999	0.05722	0.76388	0.00100	Te-1	0.975	110	10	6	1000	15	0.96
Proteus	Dataset 1	Session 6	9.19705	0.13941	0.75927	0.00116	Te-1	0.975	110	10	6	1000	15	0.96
Proteus	Dataset 2	Session 7	12.25440	0.42020	0.77446	0.00239	Te-1	1.004	110	10	6	1000	15	0.88
Proteus	Dataset 2	Session 7	10.41401	0.24236	0.76569	0.00178	Te-1	1.004	110	10	6	1000	15	0.88
Proteus	Dataset 2	Session 7	19.06979	0.27809	0.81630	0.00213	Te-1	1.004	110	10	6	1000	15	0.88
Proteus	Dataset 2	Session 7	0.02316	0.00220	0.70776	0.00020	Te-1	1.004	110	10	6	1000	15	0.88
Proteus	Dataset 2	Session 7	14.35027	0.54270	0.78556	0.00364	Te-1	1.004	110	10	6	1000	15	0.88
Proteus	Dataset 2	Session 7	14.65207	0.37638	0.78699	0.00227	Te-1	1.004	110	10	6	1000	15	0.88
Proteus	Dataset 2	Session 7	24.44625	0.53388	0.84595	0.00362	Te-1	1.004	110	10	6	1000	15	0.88
Proteus	Dataset 2	Session 7	0.01157	0.00182	0.70766	0.00017	Te-1	1.004	110	10	6	1000	15	0.88
Proteus	Dataset 2	Session 7	16.65294	0.14250	0.79920	0.00174	Te-1	1.004	110	10	6	1000	15	0.88
Proteus	Dataset 2	Session 7	13.16263	0.20333	0.78027	0.00182	Te-1	1.004	110	10	6	1000	15	0.88
Proteus	Dataset 2	Session 7	12.99420	0.25092	0.77854	0.00156	Te-1	1.004	110	10	6	1000	15	0.88
Proteus	Dataset 2	Session 7	0.06173	0.01258	0.70800	0.00021	Te-1	1.004	110	10	6	1000	15	0.88
Proteus	Dataset 2	Session 7	0.05220	0.00607	0.70790	0.00017	Te-1	1.004	110	10	6	1000	15	0.88
Proteus	Dataset 2	Session 7	15.65855	0.40791	0.79203	0.00241	Te-1	1.004	110	10	6	1000	15	0.88
Proteus	Dataset 2	Session 7	19.67463	1.82055	0.81394	0.00883	Te-1	1.004	110	10	6	1000	15	0.88
Proteus	Dataset 2	Session 7	1.19376	0.29811	0.71473	0.00170	Te-1	1.004	110	10	6	1000	15	0.88
Proteus	Dataset 2	Session 7	7.90576	1.29048	0.75128	0.00702	Te-1	1.004	110	10	6	1000	15	0.88
Proteus	Dataset 2	Session 7	14.65674	0.91592	0.78898	0.00537	Te-1	1.004	110	10	6	1000	15	0.88
Proteus	Dataset 2	Session 7	14.71433	0.33830	0.78688	0.00192	Te-1	1.004	110	10	6	1000	15	0.88
Proteus	Dataset 2	Session 7	15.82324	0.41451	0.79526	0.00209	Te-1	1.004	110	10	6	1000	15	0.88
Proteus	Dataset 2	Session 7	10.59951	0.09478	0.76796	0.00111	Te-1	1.004	110	10	6	1000	15	0.88
Proteus	Dataset 2	Session 7	0.03430	0.00265	0.70791	0.00019	Te-1	1.004	110	10	6	1000	15	0.88
Proteus	Dataset 2	Session 7	15.34158	0.48584	0.79205	0.00257	Te-1	1.004	110	10	6	1000	15	0.88
Proteus	Dataset 2	Session 7	15.15010	0.38096	0.79491	0.00245	Te-1	1.004	110	10	6	1000	15	0.88
Proteus	Dataset 2	Session 7	13.07742	0.21001	0.78162	0.00146	Te-1	1.004	110	10	6	1000	15	0.88
Proteus	Dataset 2	Session 7	13.19819	0.34017	0.78087	0.00255	Te-1	1.004	110	10	6	1000	15	0.88
Proteus	Dataset 2	Session 7	14.10176	0.31880	0.78743	0.00199	Te-1	1.004	110	10	6	1000	15	0.88
Proteus	Dataset 2	Session 7	50.59332	1.52786	0.98232	0.00903	Te-1	1.004	110	10	6	1000	15	0.88
Proteus	Dataset 2	Session 7	14.57826	0.43216	0.78922	0.00211	Te-1	1.004	110	10	6	1000	15	0.88
Proteus	Dataset 2	Session 7	13.45997	0.10530	0.78329	0.00151	Te-1	1.004	110	10	6	1000	15	0.88
Proteus	Dataset 2	Session 7	29.84395	0.43680	0.87642	0.00312	Te-1	1.004	110	10	6	1000	15	0.88
Proteus	Dataset 2	Session 7	0.03097	0.00230	0.70763	0.00015	Te-1	1.004	110	10	6	1000	15	0.88
Proteus	Dataset 2	Session 7	0.20285	0.24807	0.70808	0.00027	Te-1	1.004	110	10	6	1000	15	0.88
Proteus	Dataset 2	Session 7	9.43717	0.37396	0.75917	0.00155	Te-1	1.004	110	10	6	1000	15	0.88
Proteus	Dataset 2	Session 7	7.59909	0.11717	0.74979	0.00106	Te-1	1.004	110	10	6	1000	15	0.88
Proteus	Dataset 2	Session 7	11.90892	0.18121	0.77249	0.00171	Te-1	1.004	110	10	6	1000	15	0.88
Proteus	Dataset 2	Session 7	30.00263	1.13377	0.87253	0.00635	Te-1	1.004	110	10	6	1000	15	0.88
Proteus	Dataset 2	Session 7	5.08508	0.04158	0.73634	0.00095	Te-1	1.004	110	10	6	1000	15	0.88
Proteus	Dataset 2	Session 7	16.31062	0.31550	0.79667	0.00308	Te-1	1.004	110	10	6	1000	15	0.88
Proteus	Dataset 2	Session 7	0.04812	0.00685	0.70744	0.00018	Te-1	1.004	110	10	6	1000	15	0.88
Proteus	Dataset 2	Session 7	4.13278	0.02022	0.73040	0.00046	Te-1	1.004	110	10	6	1000	15	0.88
Proteus	Dataset 2	Session 7	1.09555	0.00431	0.71334	0.00018	Te-1	1.004	110	10	6	1000	15	0.88
Proteus	Dataset 2	Session 7	1.14419	0.00871	0.71368	0.00018	Te-1	1.004	110	10	6	1000	15	0.88
Proteus	Dataset 2	Session 7	1.16214	0.06616	0.71372	0.00030	Te-1	1.004	110	10	6	1000	15	0.88
Proteus	Dataset 2	Session 7	1.27542	0.02581	0.71457	0.00023	Te-1	1.004	110	10	6	1000	15	0.88
Proteus	Dataset 2	Session 7	1.46665	0.01775	0.71542	0.00019	Te-1	1.004	110	10	6	1000	15	0.88
Proteus	Dataset 2	Session 7	1.40810	0.02861	0.71504	0.00020	Te-1	1.004	110	10	6	1000	15	0.88
Proteus	Dataset 2	Session 8	0.02549	0.00132	0.70810	0.00022	NIST SRM610	1.004	110	10	6	1000	15	0.88
Proteus	Dataset 2	Session 8	0.03267	0.01136	0.70805	0.00018	NIST SRM610	1.004	110	10	6	1000	15	0.88
Proteus	Dataset 2	Session 8	0.34732	0.02693	0.70997	0.00031	NIST SRM610	1.004	110	10	6	1000	15	0.88
Proteus	Dataset 2	Session 8	14.07310	0.15110	0.78566	0.00109	NIST SRM610	1.004	110	10	6	1000	15	0.88
Proteus	Dataset 2	Session 8	13.74815	0.19769	0.78766	0.00159	NIST SRM610	1.004	110	10	6	1000	15	0.88
Proteus	Dataset 2	Session 8	12.74831	0.13785	0.77898	0.00176	NIST SRM610	1.004	110	10	6	1000	15	0.88
Proteus	Dataset 2	Session 8	14.83568	0.94832	0.78951	0.00242	NIST SRM610	1.004	110	10	6	1000	15	0.88
Proteus	Dataset 2	Session 8	12.14177	0.08223	0.77614	0.00118	NIST SRM610	1.004	110	10	6	1000	15	0.88
Proteus	Dataset 2	Session 8	9.55116	0.15707	0.76343	0.00132	NIST SRM610	1.004	110	10	6	1000	15	0.88
Proteus	Dataset 2	Session 8	0.01286	0.00075	0.70751	0.00025	NIST SRM610	1.004	110	10	6	1000	15	0.88
Proteus	Dataset 2	Session 8	17.77360	0.20528	0.80528	0.00172	NIST SRM610	1.004	110	10	6	1000	15	0.88
Proteus	Dataset 2	Session 8	5.49892	1.09048	0.73831	0.00613	NIST SRM610	1.004	110	10	6	1000	15	0.88
Proteus	Dataset 2	Session 8	12.60134	0.19024	0.77936	0.00160	NIST SRM610	1.004	110	10	6	1000	15	0.88
Proteus	Dataset 2	Session 8	11.69203	0.07281	0.77151	0.00148	NIST SRM610	1.004	110	10	6	1000	15	0.88
Proteus	Dataset 2	Session 8	16.62943	0.20263	0.79769	0.00153	NIST SRM610	1.004	110	10	6	1000	15	0.88
Proteus	Dataset 2	Session 8	12.26825	0.25500	0.77698	0.00196	NIST SRM610	1.004	110	10	6	1000	15	0.88
Proteus	Dataset 2	Session 8	3.65365	0.04930	0.72785	0.00058	NIST SRM610	1.004	110	10	6	1000	15	0.88
Proteus	Dataset 2	Session 8	11.01318	0.18334	0.76909	0.00110	NIST SRM610	1.004	110	10	6	1000	15	0.88
Proteus	Dataset 2	Session 8	14.06083	0.34620	0.78658	0.00205	NIST SRM610	1.004	110	10	6	1000	15	0.88
Proteus	Dataset 2	Session 8	0.03924	0.00498	0.70800	0.00026	NIST SRM610	1.004	110	10	6	1000	15	0.88
Proteus	Dataset 2	Session 8	0.00902	0.00049	0.70768	0.00018	NIST SRM610	1.004	110	10	6	1000	15	0.88
Proteus	Dataset 2	Session 8	28.99228	1.89528	0.86069	0.01096	NIST SRM610	1.004	110	10	6	1000	15	0.88

Proteus	Dataset 2	Session 8	20.88267	3.67677	0.81807	0.01908	NIST SRM610	1.004	110	10	6	1000	15	0.88
Proteus	Dataset 2	Session 8	10.36104	0.08891	0.76696	0.00125	NIST SRM610	1.004	110	10	6	1000	15	0.88
Proteus	Dataset 2	Session 8	5.41176	0.06107	0.73713	0.00068	NIST SRM610	1.004	110	10	6	1000	15	0.88
Proteus	Dataset 2	Session 8	9.57329	0.08965	0.76074	0.00135	NIST SRM610	1.004	110	10	6	1000	15	0.88
Proteus	Dataset 2	Session 8	15.43379	0.46400	0.79465	0.00276	NIST SRM610	1.004	110	10	6	1000	15	0.88
Proteus	Dataset 2	Session 8	0.03064	0.00178	0.70751	0.00020	NIST SRM610	1.004	110	10	6	1000	15	0.88
Proteus	Dataset 2	Session 8	0.00845	0.00035	0.70774	0.00020	NIST SRM610	1.004	110	10	6	1000	15	0.88
Proteus	Dataset 2	Session 8	5.83113	0.16146	0.73967	0.00092	NIST SRM610	1.004	110	10	6	1000	15	0.88
Proteus	Dataset 2	Session 8	5.44109	0.27420	0.73691	0.00157	NIST SRM610	1.004	110	10	6	1000	15	0.88
Proteus	Dataset 2	Session 8	5.48588	0.09614	0.73727	0.00064	NIST SRM610	1.004	110	10	6	1000	15	0.88
Proteus	Dataset 2	Session 8	8.12010	0.25778	0.75319	0.00133	NIST SRM610	1.004	110	10	6	1000	15	0.88
Proteus	Dataset 2	Session 8	9.66814	0.28359	0.76505	0.00199	NIST SRM610	1.004	110	10	6	1000	15	0.88
Proteus	Dataset 2	Session 8	10.21998	0.22321	0.76564	0.00139	NIST SRM610	1.004	110	10	6	1000	15	0.88
Proteus	Dataset 2	Session 8	12.86544	2.95568	0.77823	0.01632	NIST SRM610	1.004	110	10	6	1000	15	0.88
Proteus	Dataset 2	Session 8	0.01217	0.00155	0.70776	0.00018	NIST SRM610	1.004	110	10	6	1000	15	0.88
Proteus	Dataset 2	Session 8	20.24154	0.76035	0.82332	0.00593	NIST SRM610	1.004	110	10	6	1000	15	0.88
Proteus	Dataset 2	Session 8	12.15832	0.14176	0.77618	0.00145	NIST SRM610	1.004	110	10	6	1000	15	0.88
Proteus	Dataset 2	Session 8	11.93297	0.22669	0.77600	0.00181	NIST SRM610	1.004	110	10	6	1000	15	0.88
Proteus	Dataset 2	Session 8	6.87810	0.17843	0.74603	0.00114	NIST SRM610	1.004	110	10	6	1000	15	0.88
Proteus	Dataset 2	Session 8	7.19831	0.09473	0.74734	0.00100	NIST SRM610	1.004	110	10	6	1000	15	0.88
Proteus	Dataset 2	Session 8	6.88190	0.08962	0.74587	0.00115	NIST SRM610	1.004	110	10	6	1000	15	0.88
Proteus	Dataset 2	Session 8	3.22428	0.09666	0.72541	0.00047	NIST SRM610	1.004	110	10	6	1000	15	0.88
Proteus	Dataset 2	Session 8	23.51009	0.50208	0.83945	0.00373	NIST SRM610	1.004	110	10	6	1000	15	0.88
Proteus	Dataset 2	Session 8	11.10810	0.26458	0.76944	0.00144	NIST SRM610	1.004	110	10	6	1000	15	0.88
Proteus	Dataset 2	Session 8	9.59438	0.15479	0.76057	0.00117	NIST SRM610	1.004	110	10	6	1000	15	0.88
Proteus	Dataset 2	Session 8	4.40712	1.81238	0.72942	0.00896	NIST SRM610	1.004	110	10	6	1000	15	0.88
Proteus	Dataset 2	Session 8	16.01077	0.52761	0.80048	0.00331	NIST SRM610	1.004	110	10	6	1000	15	0.88
Proteus	Dataset 2	Session 8	19.29528	0.20432	0.81697	0.00234	NIST SRM610	1.004	110	10	6	1000	15	0.88
Proteus	Dataset 2	Session 8	12.87663	0.28792	0.77908	0.00219	NIST SRM610	1.004	110	10	6	1000	15	0.88
Proteus	Dataset 2	Session 8	7.83403	0.14622	0.75029	0.00134	NIST SRM610	1.004	110	10	6	1000	15	0.88
Proteus	Dataset 2	Session 8	0.02312	0.00137	0.70829	0.00029	NIST SRM610	1.004	110	10	6	1000	15	0.88
Proteus	Dataset 2	Session 8	0.19692	0.00734	0.70920	0.00034	NIST SRM610	1.004	110	10	6	1000	15	0.88
Proteus	Dataset 2	Session 8	1.59167	0.02902	0.71671	0.00030	NIST SRM610	1.004	110	10	6	1000	15	0.88
Proteus	Dataset 2	Session 8	1.23151	0.00751	0.71466	0.00035	NIST SRM610	1.004	110	10	6	1000	15	0.88
Proteus	Dataset 2	Session 8	1.27070	0.27636	0.71445	0.00033	NIST SRM610	1.004	110	10	6	1000	15	0.88
Proteus	Dataset 2	Session 8	8.03871	0.05030	0.75189	0.00095	NIST SRM610	1.004	110	10	6	1000	15	0.88
Proteus	Dataset 2	Session 8	0.01329	0.00083	0.70787	0.00023	NIST SRM610	1.004	110	10	6	1000	15	0.88
Proteus	Dataset 2	Session 8	0.01083	0.00070	0.70767	0.00026	NIST SRM610	1.004	110	10	6	1000	15	0.88
Proteus	Dataset 2	Session 8	0.01139	0.00064	0.70783	0.00033	NIST SRM610	1.004	110	10	6	1000	15	0.88
Proteus	Dataset 2	Session 8	1.36560	0.01272	0.71518	0.00028	NIST SRM610	1.004	110	10	6	1000	15	0.88
Proteus	Dataset 2	Session 8	1.44178	0.02159	0.71527	0.00033	NIST SRM610	1.004	110	10	6	1000	15	0.88
Proteus	Dataset 2	Session 8	1.53336	0.07056	0.71637	0.00046	NIST SRM610	1.004	110	10	6	1000	15	0.88
Proteus	Dataset 2	Session 8	3.08251	0.07056	0.72456	0.00055	NIST SRM610	1.004	110	10	6	1000	15	0.88
Proteus	Dataset 2	Session 8	2.54136	0.09262	0.72153	0.00081	NIST SRM610	1.004	110	10	6	1000	15	0.88
Proteus	Dataset 2	Session 8	0.04812	0.01236	0.70769	0.00023	NIST SRM610	1.004	110	10	6	1000	15	0.88
Proteus	Dataset 2	Session 8	0.07809	0.00395	0.70796	0.00024	NIST SRM610	1.004	110	10	6	1000	15	0.88
Proteus	Dataset 2	Session 8	2.07743	0.03306	0.71927	0.00035	NIST SRM610	1.004	110	10	6	1000	15	0.88
Proteus	Dataset 2	Session 8	2.03093	0.01948	0.71886	0.00042	NIST SRM610	1.004	110	10	6	1000	15	0.88
Proteus	Dataset 2	Session 9	0.22782	0.00810	0.70846	0.00031	NIST SRM610	1.004	110	10	6	1000	15	0.88
Proteus	Dataset 2	Session 9	5.06633	0.08758	0.73629	0.00071	NIST SRM610	1.004	110	10	6	1000	15	0.88
Proteus	Dataset 2	Session 9	18.09159	0.39456	0.80783	0.00215	NIST SRM610	1.004	110	10	6	1000	15	0.88
Proteus	Dataset 2	Session 9	16.13870	0.31369	0.79792	0.00199	NIST SRM610	1.004	110	10	6	1000	15	0.88
Proteus	Dataset 2	Session 9	4.33791	0.14481	0.73235	0.00077	NIST SRM610	1.004	110	10	6	1000	15	0.88
Proteus	Dataset 2	Session 9	0.23114	0.00828	0.70841	0.00024	NIST SRM610	1.004	110	10	6	1000	15	0.88
Proteus	Dataset 2	Session 9	6.20434	0.28213	0.74193	0.00119	NIST SRM610	1.004	110	10	6	1000	15	0.88
Proteus	Dataset 2	Session 9	38.63894	1.55862	0.92094	0.01077	NIST SRM610	1.004	110	10	6	1000	15	0.88
Proteus	Dataset 2	Session 9	20.76549	2.66465	0.81768	0.01208	NIST SRM610	1.004	110	10	6	1000	15	0.88
Proteus	Dataset 2	Session 9	4.69366	0.38557	0.73694	0.00385	NIST SRM610	1.004	110	10	6	1000	15	0.88
Proteus	Dataset 2	Session 9	3.95376	0.08458	0.72958	0.00078	NIST SRM610	1.004	110	10	6	1000	15	0.88
Proteus	Dataset 2	Session 9	5.56189	0.09923	0.73820	0.00078	NIST SRM610	1.004	110	10	6	1000	15	0.88
Proteus	Dataset 2	Session 9	22.12513	0.30370	0.83007	0.00215	NIST SRM610	1.004	110	10	6	1000	15	0.88
Proteus	Dataset 2	Session 9	14.17053	0.26544	0.78499	0.00176	NIST SRM610	1.004	110	10	6	1000	15	0.88
Proteus	Dataset 2	Session 9	20.61915	3.26884	0.82534	0.01967	NIST SRM610	1.004	110	10	6	1000	15	0.88
Proteus	Dataset 2	Session 9	7.16655	0.09323	0.74724	0.00108	NIST SRM610	1.004	110	10	6	1000	15	0.88
Proteus	Dataset 2	Session 9	6.20859	0.13770	0.74153	0.00090	NIST SRM610	1.004	110	10	6	1000	15	0.88
Proteus	Dataset 2	Session 9	5.69107	0.11614	0.73854	0.00071	NIST SRM610	1.004	110	10	6	1000	15	0.88

Table S6: Data plotted in Figure 10 & sample ion yield calculated for Agilent 8800 from 2 million counts per second of ^{88}Sr reported for NIST SRM610 with 49% Sr to SrF conversion efficiency when using SF_6 reaction gas (Hogmalm et al., 2017).

Sample: NIST SRM610

Instrument	Sample ion yield (%)	$^{87}Sr/^{86}Sr$ 2RSE	Spot diameter μm	Repetiton rate (Hz)	Fluence (J/cm^2)	Pulse count
Proteus	0.05	0.00035	110	10	6	600
Proteus	0.05	0.00046	110	10	6	600
Proteus	0.05	0.00033	110	10	6	600
Proteus	0.05	0.00030	110	10	6	600
Proteus	0.05	0.00028	110	10	6	600
Proteus	0.05	0.00038	110	10	6	600
iCAP-TQ	0.0003	0.0087	110	10	6	600
iCAP-TQ	0.0003	0.0086	110	10	6	600
iCAP-TQ	0.0004	0.0089	110	10	6	600
iCAP-TQ	0.0004	0.0085	110	10	6	600
iCAP-TQ	0.0004	0.0084	110	10	6	600
iCAP-TQ	0.0003	0.0087	110	10	6	600
Agilent 8800	0.006		50	10	7	

Table S7: Data plotted in Figure 11

Sample: DG-1

Instrument	Dataset	Analysis Session	$^{87}Rb/^{86}Sr$	2SE	$^{87}Sr/^{86}Sr$	2SE	$^{87}Sr/^{86}Sr$ standard	$^{87}Rb/^{86}Sr$ correction factor	Spot diameter μm	Repetiton rate (Hz)	Fluence (J/cm^2)	Laser pulse count	Torch Depth (mm)	Sample gas (l/min)
Proteus	Dataset 3	Session 8	0.15647	0.05867	0.71036	0.00059	NIST SRM610	0.997	110	10	6	600	15	0.88
Proteus	Dataset 3	Session 8	0.05982	0.00459	0.70962	0.00064	NIST SRM610	0.997	110	10	6	600	15	0.88
Proteus	Dataset 3	Session 8	0.06199	0.01124	0.70898	0.00073	NIST SRM610	0.997	110	10	6	600	15	0.88
Proteus	Dataset 3	Session 8	0.03179	0.00106	0.70986	0.00060	NIST SRM610	0.997	110	10	6	600	15	0.88
Proteus	Dataset 3	Session 8	20.27922	0.51534	0.79195	0.00314	NIST SRM610	0.997	110	10	6	600	15	0.88
Proteus	Dataset 3	Session 8	25.87102	0.51662	0.81381	0.00262	NIST SRM610	0.997	110	10	6	600	15	0.88
Proteus	Dataset 3	Session 8	22.77867	0.40329	0.79909	0.00227	NIST SRM610	0.997	110	10	6	600	15	0.88
Proteus	Dataset 3	Session 8	11.09805	0.21022	0.75228	0.00189	NIST SRM610	0.997	110	10	6	600	15	0.88
Proteus	Dataset 3	Session 8	23.40030	0.25162	0.80181	0.00261	NIST SRM610	0.997	110	10	6	600	15	0.88
Proteus	Dataset 3	Session 8	26.01956	0.52616	0.81564	0.00423	NIST SRM610	0.997	110	10	6	600	15	0.88
Proteus	Dataset 3	Session 8	23.41687	0.33944	0.80176	0.00272	NIST SRM610	0.997	110	10	6	600	15	0.88
Proteus	Dataset 3	Session 8	22.06416	0.24104	0.79987	0.00284	NIST SRM610	0.997	110	10	6	600	15	0.88
Proteus	Dataset 3	Session 8	25.41640	0.25375	0.81100	0.00317	NIST SRM610	0.997	110	10	6	600	15	0.88
Proteus	Dataset 3	Session 8	27.22180	0.45435	0.81686	0.00298	NIST SRM610	0.997	110	10	6	600	15	0.88
Proteus	Dataset 3	Session 8	9.81082	0.29318	0.74774	0.00151	NIST SRM610	0.997	110	10	6	600	15	0.88
Proteus	Dataset 3	Session 8	24.24648	0.26958	0.80456	0.00305	NIST SRM610	0.997	110	10	6	600	15	0.88
Proteus	Dataset 3	Session 8	23.42339	0.19341	0.80421	0.00301	NIST SRM610	0.997	110	10	6	600	15	0.88
iCAP TQ			21.07779	0.29853	0.79747	0.00769	NIST SRM610	1.007	110	10	6	600	5	0.78
iCAP TQ			14.65313	0.25987	0.77015	0.00805	NIST SRM610	1.007	110	10	6	600	5	0.78
iCAP TQ			18.51711	0.42427	0.78274	0.00716	NIST SRM610	1.007	110	10	6	600	5	0.78
iCAP TQ			13.48329	0.25645	0.76813	0.00726	NIST SRM610	1.007	110	10	6	600	5	0.78
iCAP TQ			19.31580	0.30230	0.77578	0.00884	NIST SRM610	1.007	110	10	6	600	5	0.78
iCAP TQ			17.94930	0.21014	0.77659	0.00745	NIST SRM610	1.007	110	10	6	600	5	0.78
iCAP TQ			17.28124	0.28149	0.77157	0.00969	NIST SRM610	1.007	110	10	6	600	5	0.78
iCAP TQ			18.11955	0.40214	0.78855	0.01024	NIST SRM610	1.007	110	10	6	600	5	0.78
iCAP TQ			15.67324	0.25441	0.77127	0.01091	NIST SRM610	1.007	110	10	6	600	5	0.78
iCAP TQ			28.57226	0.57759	0.82454	0.00916	NIST SRM610	1.007	110	10	6	600	5	0.78
iCAP TQ			15.18125	0.35066	0.77275	0.01002	NIST SRM610	1.007	110	10	6	600	5	0.78
iCAP TQ			21.64559	0.52738	0.79804	0.01027	NIST SRM610	1.007	110	10	6	600	5	0.78
iCAP TQ			14.73862	0.38375	0.76534	0.01029	NIST SRM610	1.007	110	10	6	600	5	0.78
iCAP TQ			0.11637	0.14016	0.71089	0.00678	NIST SRM610	1.007	110	10	6	600	5	0.78
iCAP TQ			0.05677	0.00260	0.70945	0.00502	NIST SRM610	1.007	110	10	6	600	5	0.78
iCAP TQ			0.20750	0.34122	0.70755	0.00667	NIST SRM610	1.007	110	10	6	600	5	0.78
iCAP TQ			0.11199	0.07994	0.71155	0.00743	NIST SRM610	1.007	110	10	6	600	5	0.78

Table S8: Age resolution model outputs (Fig.12 & 13)

		Proteus	8800 QQQ	iCAP-TQ
$^{87}Rb/^{86}Sr$ range	Age (Ma)	(2σ % of age)	(2σ % of age)	(2σ % of age)
0-1	20	140.19	497.06	1254.33
0-1	40	70.10	248.54	627.16
0-1	60	46.74	165.70	418.10
0-1	80	35.06	124.28	313.57
0-1	100	28.05	99.43	250.86
0-1	120	23.39	82.86	209.04
0-1	140	20.05	71.03	179.18
0-1	160	17.55	62.15	156.78
0-1	180	15.61	55.25	139.36
0-1	200	14.06	49.73	125.42
0-1	300	9.40	33.17	83.61
0-1	400	7.08	24.89	62.71
0-1	500	5.70	19.93	50.17
0-1	600	4.78	16.63	41.81
0-1	700	4.13	14.27	35.84
0-1	800	3.65	12.50	31.36
0-1	900	3.28	11.13	27.88
0-1	1000	2.98	10.03	25.09
0-1	1100	2.74	9.13	22.82
0-1	1200	2.55	8.39	20.92
0-1	1300	2.38	7.76	19.31
0-1	1400	2.24	7.22	17.94
0-1	1500	2.12	6.76	16.74
0-1	1600	2.01	6.35	15.70
0-1	1700	1.92	5.99	14.78
0-1	1800	1.84	5.67	13.96
0-1	1900	1.77	5.39	13.23
0-1	2000	1.71	5.14	12.57
0-1	3000	1.32	3.54	8.41
0-1	4000	1.14	2.77	6.35
0-3	20	46.71	165.67	417.91
0-3	40	23.38	82.87	209.01
0-3	60	15.61	55.27	139.38
0-3	80	11.73	41.48	104.56
0-3	100	9.41	33.20	83.67
0-3	120	7.86	27.68	69.74
0-3	140	6.76	23.75	59.80
0-3	160	5.94	20.79	52.34
0-3	180	5.30	18.50	46.54
0-3	200	4.79	16.66	41.90
0-3	300	3.29	11.17	27.98
0-3	400	2.56	8.43	21.02
0-3	500	2.13	6.80	16.85
0-3	600	1.85	5.72	14.07
0-3	700	1.66	4.95	12.09
0-3	800	1.52	4.38	10.60
0-3	900	1.41	3.94	9.45
0-3	1000	1.33	3.59	8.52
0-3	1100	1.26	3.30	7.77
0-3	1200	1.21	3.07	7.14
0-3	1300	1.16	2.87	6.61
0-3	1400	1.13	2.70	6.16
0-3	1500	1.09	2.56	5.77
0-3	1600	1.06	2.43	5.43
0-3	1700	1.04	2.33	5.12
0-3	1800	1.02	2.23	4.86
0-3	1900	1.00	2.14	4.62
0-3	2000	0.98	2.07	4.40
0-3	3000	0.88	1.59	3.06
0-3	4000	0.83	1.36	2.41
0-30	20	4.80	3.48	8.74
0-30	40	2.56	3.30	8.23
0-30	60	1.86	3.09	7.60
0-30	80	1.52	2.88	6.98
0-30	100	1.34	2.70	6.42
0-30	120	1.21	2.54	5.92
0-30	140	1.13	2.41	5.50
0-30	160	1.07	2.30	5.12
0-30	180	1.02	2.20	4.80
0-30	200	0.99	2.11	4.52
0-30	300	0.88	1.83	3.54
0-30	400	0.83	1.66	2.96
0-30	500	0.80	1.55	2.59
0-30	600	0.78	1.47	2.33
0-30	700	0.77	1.41	2.15
0-30	800	0.76	1.36	2.00
0-30	900	0.75	1.33	1.89
0-30	1000	0.75	1.29	1.80
0-30	1100	0.74	1.27	1.73
0-30	1200	0.74	1.24	1.66
0-30	1300	0.73	1.22	1.61
0-30	1400	0.73	1.21	1.57
0-30	1500	0.73	1.19	1.53
0-30	1600	0.73	1.18	1.49
0-30	1700	0.73	1.17	1.46
0-30	1800	0.73	1.15	1.43
0-30	1900	0.73	1.14	1.41
0-30	2000	0.72	1.14	1.39
0-30	3000	0.72	1.08	1.25
0-30	4000	0.72	1.05	1.18
0-300	20	1.54	3.18	10.52
0-300	40	1.07	1.84	5.56
0-300	60	0.94	1.42	3.91
0-300	80	0.87	1.23	3.10
0-300	100	0.84	1.11	2.62
0-300	120	0.81	1.04	2.30
0-300	140	0.80	0.99	2.08
0-300	160	0.78	0.95	1.91
0-300	180	0.77	0.92	1.78
0-300	200	0.77	0.90	1.68
0-300	300	0.74	0.83	1.37
0-300	400	0.74	0.80	1.22
0-300	500	0.73	0.78	1.13
0-300	600	0.73	0.76	1.07
0-300	700	0.72	0.76	1.03
0-300	800	0.72	0.75	1.00
0-300	900	0.72	0.75	0.98
0-300	1000	0.72	0.74	0.96
0-300	1100	0.72	0.74	0.94
0-300	1200	0.72	0.74	0.93
0-300	1300	0.72	0.74	0.91
0-300	1400	0.72	0.73	0.90
0-300	1500	0.72	0.73	0.89
0-300	1600	0.72	0.73	0.89
0-300	1700	0.72	0.73	0.88
0-300	1800	0.72	0.73	0.87
0-300	1900	0.72	0.73	0.87

0-300	2000	0.72	0.73	0.86
0-300	3000	0.72	0.73	0.83
0-300	4000	0.72	0.72	0.82

Table S9: $^{87}Rb/^{86}Sr$ and Sr concentrations used in theoretical age resolution model

$^{87}Rb/^{86}Sr$ (0-1)	[Sr] (μg/g)	$^{87}Rb/^{86}Sr$ (0-3)	[Sr] (μg/g)	$^{87}Rb/^{86}Sr$ (0-30)	[Sr] (μg/g)	$^{87}Rb/^{86}Sr$ (0-300)	[Sr] (μg/g)
0.001	500	0.001	500	0.001	500	0.001	500
0.1	50	0.3	50	3	50	30	5
0.2	50	0.6	50	6	50	60	5
0.3	50	0.9	50	9	50	90	5
0.4	50	1.2	50	12	50	120	5
0.5	50	1.5	50	15	50	150	5
0.6	50	1.8	50	18	50	180	5
0.7	50	2.1	50	21	50	210	5
0.8	50	2.4	50	24	50	240	5
0.9	50	2.7	50	27	50	270	5
1.0	50	3.0	50	30	50	300	5

Table S10: Data plotted in Figure 12

Sample: SG-1 (Single K-feldspar)

Instrument	Dataset	Analysis Session	$^{87}Rb/^{86}Sr$	2SE	$^{87}Sr/^{86}Sr$	2SE	$^{87}Sr/^{86}Sr$ standard	$^{87}Rb/^{86}Sr$ correction factor	Spot number	Spot diameter μm	Repetiton rate (Hz)	Fluence (J/cm^2)	Laser pulse count	Torch Depth (mm)	Sample gas (l/min)
Proteus	Dataset 1	Session 4	0.02871	0.00550	0.70746	0.00027	Te-1	0.975	1	110	10	6	1000	15	0.96
Proteus	Dataset 1	Session 4	0.07044	0.00626	0.70759	0.00027	Te-1	0.975	2	110	10	6	1000	15	0.96
Proteus	Dataset 1	Session 4	1.76785	0.04877	0.71744	0.00044	Te-1	0.975	3	110	10	6	1000	15	0.96
Proteus	Dataset 1	Session 4	1.34318	0.02837	0.71502	0.00027	Te-1	0.975	4	110	10	6	1000	15	0.96
Proteus	Dataset 1	Session 4	2.25158	0.07591	0.71991	0.00029	Te-1	0.975	5	110	10	6	1000	15	0.96
Proteus	Dataset 1	Session 4	2.04773	0.01768	0.71900	0.00030	Te-1	0.975	6	110	10	6	1000	15	0.96
Proteus	Dataset 1	Session 4	2.74740	0.24095	0.72264	0.00144	Te-1	0.975	7	110	10	6	1000	15	0.96
Proteus	Dataset 1	Session 4	2.31938	0.03766	0.71989	0.00033	Te-1	0.975	8	110	10	6	1000	15	0.96
Proteus	Dataset 1	Session 4	2.27023	0.15827	0.71976	0.00034	Te-1	0.975	9	110	10	6	1000	15	0.96
Proteus	Dataset 1	Session 4	2.27715	0.05809	0.71998	0.00042	Te-1	0.975	10	110	10	6	1000	15	0.96
Proteus	Dataset 1	Session 4	2.16379	0.08985	0.71951	0.00063	Te-1	0.975	11	110	10	6	1000	15	0.96
Proteus	Dataset 1	Session 4	1.47772	0.01955	0.71572	0.00032	Te-1	0.975	12	110	10	6	1000	15	0.96
Proteus	Dataset 1	Session 4	2.18168	0.08321	0.71920	0.00066	Te-1	0.975	13	110	10	6	1000	15	0.96
Proteus	Dataset 1	Session 4	1.67326	0.03224	0.71670	0.00032	Te-1	0.975	14	110	10	6	1000	15	0.96
Proteus	Dataset 1	Session 4	2.62185	0.18741	0.72200	0.00030	Te-1	0.975	15	110	10	6	1000	15	0.96
Proteus	Dataset 1	Session 4	1.27674	0.01760	0.71464	0.00021	Te-1	0.975	16	110	10	6	1000	15	0.96
Proteus	Dataset 1	Session 4	1.80464	0.03216	0.71755	0.00025	Te-1	0.975	17	110	10	6	1000	15	0.96
Proteus	Dataset 1	Session 4	2.33983	0.05687	0.72028	0.00035	Te-1	0.975	18	110	10	6	1000	15	0.96
Proteus	Dataset 1	Session 4	1.80669	0.11292	0.71742	0.00030	Te-1	0.975	19	110	10	6	1000	15	0.96
Proteus	Dataset 1	Session 4	2.07649	0.08256	0.71934	0.00043	Te-1	0.975	20	110	10	6	1000	15	0.96
Proteus	Dataset 1	Session 4	2.17952	0.12909	0.71945	0.00050	Te-1	0.975	21	110	10	6	1000	15	0.96
Proteus	Dataset 1	Session 4	2.12129	0.13577	0.71957	0.00082	Te-1	0.975	22	110	10	6	1000	15	0.96
Proteus	Dataset 1	Session 4	2.44123	0.09530	0.72069	0.00049	Te-1	0.975	23	110	10	6	1000	15	0.96
Proteus	Dataset 1	Session 4	4.39818	0.13376	0.73131	0.00095	Te-1	0.975	24	110	10	6	1000	15	0.96
Proteus	Dataset 1	Session 4	4.70285	0.14042	0.73336	0.00089	Te-1	0.975	25	110	10	6	1000	15	0.96
Proteus	Dataset 1	Session 4	4.02226	0.05838	0.72950	0.00041	Te-1	0.975	26	110	10	6	1000	15	0.96
Proteus	Dataset 1	Session 4	1.19154	0.15343	0.71381	0.00054	Te-1	0.975	27	110	10	6	1000	15	0.96
Proteus	Dataset 1	Session 4	2.90751	0.13242	0.72340	0.00068	Te-1	0.975	28	110	10	6	1000	15	0.96
Proteus	Dataset 1	Session 4	2.20928	0.04172	0.71998	0.00039	Te-1	0.975	29	110	10	6	1000	15	0.96
Proteus	Dataset 1	Session 4	1.99519	0.04374	0.71860	0.00035	Te-1	0.975	30	110	10	6	1000	15	0.96
Proteus	Dataset 1	Session 4	2.24122	0.04268	0.71995	0.00033	Te-1	0.975	31	110	10	6	1000	15	0.96
Proteus	Dataset 1	Session 4	3.61710	0.09764	0.72734	0.00062	Te-1	0.975	32	110	10	6	1000	15	0.96
Proteus	Dataset 1	Session 4	1.77040	0.04321	0.71725	0.00030	Te-1	0.975	33	110	10	6	1000	15	0.96
Proteus	Dataset 1	Session 4	2.19415	0.04389	0.72001	0.00033	Te-1	0.975	34	110	10	6	1000	15	0.96
Proteus	Dataset 2	Session 8	0.01071	0.00071	0.70767	0.00026	NIST SRM610	1.004	35	110	10	6	1000	15	0.88
Proteus	Dataset 2	Session 8	1.36559	0.01271	0.71515	0.00028	NIST SRM610	1.004	36	110	10	6	1000	15	0.88
Proteus	Dataset 2	Session 8	1.44176	0.02159	0.71524	0.00033	NIST SRM610	1.004	37	110	10	6	1000	15	0.88
Proteus	Dataset 2	Session 8	1.53333	0.07056	0.71636	0.00047	NIST SRM610	1.004	38	110	10	6	1000	15	0.88
Proteus	Dataset 2	Session 8	3.08282	0.07061	0.72450	0.00055	NIST SRM610	1.004	39	110	10	6	1000	15	0.88
Proteus	Dataset 2	Session 8	2.54154	0.09266	0.72149	0.00080	NIST SRM610	1.004	40	110	10	6	1000	15	0.88
Proteus	Dataset 2	Session 8	0.07824	0.00396	0.70820	0.00024	NIST SRM610	1.004	41	110	10	6	1000	15	0.88
Proteus	Dataset 2	Session 8	4.13015	0.27175	0.72846	0.00130	NIST SRM610	1.004	42	110	10	6	1000	15	0.88
Proteus	Dataset 2	Session 8	2.08437	0.03316	0.71948	0.00035	NIST SRM610	1.004	43	110	10	6	1000	15	0.88
Proteus	Dataset 2	Session 8	2.03770	0.01953	0.71907	0.00042	NIST SRM610	1.004	44	110	10	6	1000	15	0.88
Proteus	Dataset 2	Session 8	1.81135	0.12981	0.71751	0.00072	NIST SRM610	1.004	45	110	10	6	1000	15	0.88
Proteus	Dataset 2	Session 8	2.23395	0.05986	0.71995	0.00049	NIST SRM610	1.004	46	110	10	6	1000	15	0.88
Proteus	Dataset 2	Session 8	1.62988	0.03198	0.71657	0.00042	NIST SRM610	1.004	47	110	10	6	1000	15	0.88
Proteus	Dataset 2	Session 8	2.44966	0.02062	0.72161	0.00047	NIST SRM610	1.004	48	110	10	6	1000	15	0.88
Proteus	Dataset 2	Session 8	2.09227	0.06097	0.71924	0.00037	NIST SRM610	1.004	49	110	10	6	1000	15	0.88
Proteus	Dataset 2	Session 8	0.66745	0.03070	0.71148	0.00037	NIST SRM610	1.004	50	110	10	6	1000	15	0.88
Proteus	Dataset 2	Session 8	3.47617	0.35374	0.72656	0.00196	NIST SRM610	1.004	51	110	10	6	1000	15	0.88
Proteus	Dataset 2	Session 8	2.11306	0.02200	0.71938	0.00041	NIST SRM610	1.004	52	110	10	6	1000	15	0.88
Proteus	Dataset 2	Session 8	2.48047	0.05208	0.72127	0.00041	NIST SRM610	1.004	53	110	10	6	1000	15	0.88
Proteus	Dataset 2	Session 8	1.54229	0.01841	0.71664	0.00034	NIST SRM610	1.004	54	110	10	6	1000	15	0.88
Proteus	Dataset 2	Session 8	1.32541	0.17834	0.71505	0.00102	NIST SRM610	1.004	55	110	10	6	1000	15	0.88
Proteus	Dataset 2	Session 8	2.09024	0.03759	0.71922	0.00041	NIST SRM610	1.004	56	110	10	6	1000	15	0.88
Proteus	Dataset 2	Session 8	0.93589	0.17972	0.71320	0.00097	NIST SRM610	1.004	57	110	10	6	1000	15	0.88
Proteus	Dataset 2	Session 8	2.37060	0.07377	0.72113	0.00051	NIST SRM610	1.004	58	110	10	6	1000	15	0.88
Proteus	Dataset 2	Session 8	2.12413	0.07771	0.71965	0.00045	NIST SRM610	1.004	59	110	10	6	1000	15	0.88
Proteus	Dataset 2	Session 8	1.55361	0.04023	0.71646	0.00042	NIST SRM610	1.004	60	110	10	6	1000	15	0.88
Proteus	Dataset 2	Session 8	2.25665	0.03665	0.72035	0.00048	NIST SRM610	1.004	61	110	10	6	1000	15	0.88
Proteus	Dataset 2	Session 8	3.68965	0.08034	0.72874	0.00075	NIST SRM610	1.004	62	110	10	6	1000	15	0.88
Proteus	Dataset 2	Session 8	4.06941	0.10791	0.73024	0.00113	NIST SRM610	1.004	63	110	10	6	1000	15	0.88
Proteus	Dataset 2	Session 8	1.72676	0.03787	0.71746	0.00034	NIST SRM610	1.004	64	110	10	6	1000	15	0.88
Proteus	Dataset 2	Session 8	2.51708	0.05425	0.72271	0.00630	NIST SRM610	1.004	65	110	10	6	1000	15	0.88
Proteus	Dataset 2	Session 8	3.18718	0.05162	0.72535	0.00069	NIST SRM610	1.004	66	110	10	6	1000	15	0.88
Proteus	Dataset 2	Session 8	2.16186	0.03601	0.71974	0.00047	NIST SRM610	1.004	67	110	10	6	1000	15	0.88
Proteus	Dataset 2	Session 8	3.04238	0.08421	0.72482	0.00064	NIST SRM610	1.004	68	110	10	6	1000	15	0.88
Proteus	Dataset 2	Session 8	1.02190	0.09502	0.71332	0.00073	NIST SRM610	1.004	69	110	10	6	1000	15	0.88
Proteus	Dataset 2	Session 8	3.20805	0.28652	0.72568	0.00173	NIST SRM610	1.004	70	110	10	6	1000	15	0.88
